# Protocol for targeted analysis of oxylipins in rat plasma and mouse macrophage samples by high-resolution UHPLC-MS/MS

**DOI:** 10.1016/j.xpro.2026.104834

**Published:** 2026-09-18

**Authors:** Larissa R. Diniz, Adriano B. Chaves-Filho, Pedro H.F. Jordão, Rosangela S. Santos, Marina T. Botana, Johan Kolmert, Ana C.A. Zucão, Marteinn T. Snaebjornsson, Eduardo M. Reis, Almut Schulze, Britta Brügger, Craig E. Wheelock, Sayuri Miyamoto

**Affiliations:** 1 Departamento de Bioquímica, Instituto de Química, Universidade de São Paulo, São Paulo, SP 05508-000, Brazil; 2 Centro de Metabolismo (CoMETA), Hospital Universitário, Universidade de São Paulo, São Paulo, SP 05508-000, Brazil; 3 Division of Tumor Metabolism and Microenvironment, German Cancer Research Center (DKFZ), Im Neuenheimer Feld 581, 69120 Heidelberg, Germany; 4 Heidelberg University Biochemistry Center (BZH), Heidelberg University, Im Neuenheimer Feld 328, 69120 Heidelberg, Germany; 5 Unit of Integrative Metabolomics, Institute of Environmental Medicine, Karolinska Institute, 17177 Stockholm, Sweden; 6 Department of Respiratory Medicine and Allergy, Karolinska University Hospital, 17176 Stockholm, Sweden

**Keywords:** Mass Spectrometry, Metabolomics, Protocols in Metabolomics and Lipidomics

## Abstract

Oxylipins are bioactive oxygenated fatty acid derivatives that act as signaling mediators in diverse physiological and pathological processes. Here, we present a protocol for the simultaneous analysis of 125 oxylipins in rat plasma and mouse macrophage samples. We describe steps for preparing standards and calibration curves, extracting oxylipins, and performing ultra-high-performance liquid chromatography coupled to high-resolution tandem mass spectrometry (UHPLC-MS/MS). We also detail procedures for using *staRoxy*, an original R package for oxylipin abundance data analysis.

For complete details on the use and execution of this protocol, please refer to Chaves-Filho et al.,^1^ Kolmert et al.^2^ and Yapici et al.^3^

## Highlights

• Setup of a targeted LC-MS workflow and preparation of analytical standards
• Oxylipin extraction from rat plasma and macrophage cell culture samples
• Broad detection and quantification of targeted oxylipin species
• Bioinformatic analysis of oxylipin datasets using the *staRoxy* R package

## Abstract (editor highlights)

Publisher’s note: Undertaking any experimental protocol requires adherence to local institutional guidelines for laboratory safety and ethics.

## Graphical abstract

**Figure:**
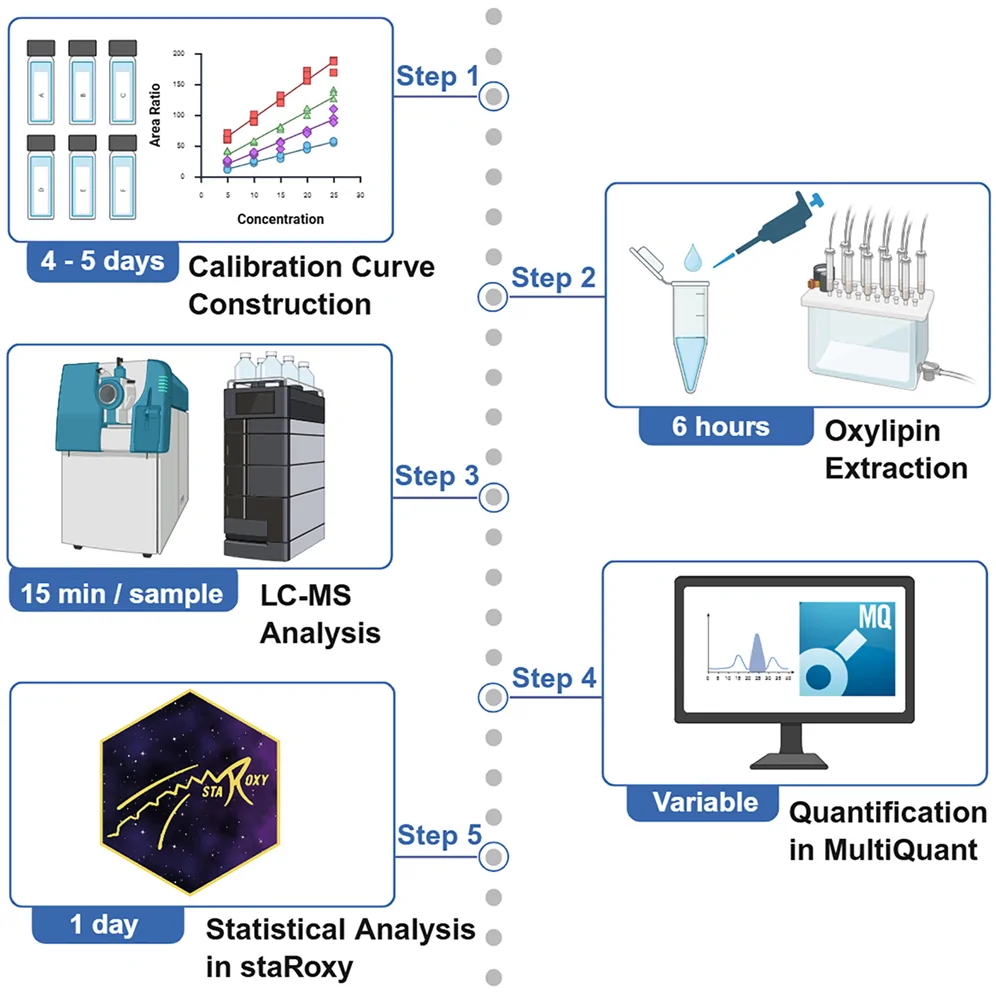

## Before you begin

The following protocol provides a comprehensive, step-by-step workflow for establishing a high-resolution *tandem* UHPLC-MS/MS method for the analysis of 125 oxylipin species in biological samples (blood plasma and cell culture). Monitored species include 41 mono-hydroxy compounds, 6 di-hydroxy compounds, 6 ketones, 13 epoxides, 29 prostaglandins, 7 leukotrienes, 4 lipoxins, 4 thromboxanes, 5 resolvins, 1 protectin, 2 maresins, 5 isoprostanes and 2 nitrated fatty acids.

This protocol is organized into sequential sections designed to achieve the most efficient workflow, starting with the preparation of standard solutions and analytical method constructions, followed by sample processing and instrumental analysis, and concluding with data processing and statistical analysis.

### General considerations

Before starting this protocol, verify the cleanliness of the mass spectrometer, with particular attention to the ion source and ion transfer optics (e.g., Q-jet, S-lens, or ion funnel), as high levels of contamination can adversely affect MS data quality. Perform mass calibration using appropriate calibration solutions according to the manufacturer’s and laboratory guidelines.

Verify the cleanliness of the LC system and column to prevent retention time shifts and system clogging. Wash all glassware thoroughly using MS-grade solvents to minimize contamination, or, ideally, use glassware dedicated exclusively to MS preparations. Use high-quality MS-grade solvents throughout the protocol.

### Oxylipin nomenclature

Although recent efforts have aimed to standardize oxylipin nomenclature, naming of the same oxylipin species may vary across the literature and vendor catalogs. For consistency, tables and supplementary materials in this protocol retained the reagent names as listed in vendor catalogs, to avoid discrepancies that could lead to acquisition of incorrect standards.

Nevertheless, *staRoxy* is configured to report oxylipins using the systematic nomenclature and LIPID MAPS-style abbreviations.^4^ Because the LC-MS/MS workflow described here does not determine stereochemistry, analyte names are reported only at the level of structural assignment supported by the method, and stereochemical descriptors are omitted. For unresolved isomeric species, combined annotations are used when appropriate.

Accordingly, Table S1 provides a harmonized reference linking vendor catalog names, accepted synonyms, *staRoxy* abbreviations, and precursor fatty acids. We strongly recommend the use of standardized oxylipin nomenclature, even when *staRoxy* is not used.

### Innovation

The method described in this protocol enables the simultaneous analysis of 125 different species of oxylipin derived from 9 fatty acid precursors, including arachidonic (ARA), adrenic (AdA), linoleic (LA), linolenic (ALA), γ-linolenic (GLA), dihomo-γ-linolenic (DGLA), oleic (OL), eicosapentaenoic (EPA) and docosahexaenoic (DHA) acids. This comprehensive approach allows the integrated characterization of inflammatory and pro-resolving lipid mediators, capturing the complex and dynamic lipid dynamic signaling across different stages of the inflammatory response. In addition, we introduce *staRoxy*, an original R package specially developed to provide statistical tools tailored for oxylipin data analysis.

### Institutional permissions

The protocol performed by Chaves-Filho et al.^1^ uses plasma samples collected from rats overexpressing human *SOD1*-G93A. Animal studies were conducted according to the ethical principles of animal experimentation and performed according to the guidelines of the National Council for Animal Experimentation Control (Conselho Nacional de Controle de Experimentação Animal – CONCEA, Ministry of Science, Technology, Innovation and Communications, Brazil) under the protocol number 14/2013.

### Sample preparation

**Timing: 30 min**

This section describes the procedures during sample collection, handling, and storage that are critical to ensure high-quality oxylipin analysis.

1. Plasma samples.
  a. Collect blood from rats by cardiac puncture into blood collecting tubes.
    **CRITICAL:** Blood should be collected into tubes containing an anticoagulant. Ethylenediaminetetraacetic acid (EDTA) plasma is generally preferred over serum and heparin plasma to minimize ex vivo cell activation and lipase activity.^5^^,^^6^
  b. Invert the tubes 3–4 times to ensure proper mixing of blood and anticoagulant.
  c. Centrifuge the tubes at 2,000 × *g* for 10 min at 4°C.
  d. Transfer the plasma fraction to a cryogenic tube.
  e. Store plasma samples at −80°C within 1 h after collection.
    **CRITICAL:** Blood and plasma samples should be maintained at low temperatures at all times during handling and processed as quickly as possible to avoid enzyme activity and artificial oxylipin formation.
2. Cell culture samples.
  a. Culture medium.
    i. Collect the culture medium from the culture vessel into 15 mL plastic tubes.
    ii. Centrifuge at 2,000 × *g* for 10 min at 4°C.
    iii. Transfer the supernatant to labeled tubes.
    iv. For no-adherent culture, retain the cell pellet for PBS washing step (see section b. Cell pellets).
    v. Store at −80°C until oxylipin extraction.
  b. Cell pellets.
    i. For adherent cultures, detach the cells using trypsin.
    ii. Centrifuge at 2,000 × *g* for 10 min at 4°C.
    iii. Discard the supernatant.
    iv. Wash the cell pellet with 2 mL of PBS.
    v. Centrifuge at 2,000 × *g* for 10 min at 4°C.
    vi. Discard the supernatant.
    vii. Add 500 μL of PBS and homogenize by pipetting up and down.
    viii. Transfer the cell homogenates to labeled tubes.
    ix. Remove 50 μL for protein quantification.
    x. Store at −80°C within 1 h after collection.

**CRITICAL:** Oxylipin levels in cell pellets may be normalized by cell number or by total protein content. Accurate cell counts should be recorded for each sample when normalization by cell number is intended. Alternatively, for protein normalization, a separate aliquot of approximately 50 μL of the cell homogenate should be reserved for protein quantification.

**CRITICAL:** To minimize degradation, all types of samples should be stored at −80°C and ideally extracted within 1 year of collection.^7^

**CRITICAL:** Oxylipins exist at low concentrations in biological samples, therefore, a relatively high amount of starting material is required for reliable detection. The ideal amount may vary depending on the sample type. However, general minimum input amounts are as follows: plasma (500 μL); cells (10^6^ cells); culture medium (1 mL from a total volume of 4mL from a culture of 10^6^ cells).

***Note:*** Oxylipins may be detected in both cell pellet and culture medium. Thus, analysis of both fractions is recommended to obtain a more comprehensive oxylipin profile. Note that fetal bovine serum (FBS), commonly used as the main nutrient source in most cell culture protocols, naturally contains oxylipins and their fatty acid precursors. Therefore, complete medium samples (+FBS/ - cells) should be included as controls to account for background oxylipin levels present in the medium.

***Note:*** The use of phenol red-free media is strongly recommended, as phenol red may contaminate HPLC columns and interfere with mass spectrometer analysis.

## Key resources table

**Table:** 

REAGENT or RESOURCE	SOURCE	IDENTIFIER
**Chemicals, peptides, and recombinant proteins**		

Water, LC-MS grade	Fisher	W6-212
Isopropanol, LC-MS grade	Fisher	A461-212
Acetonitrile, LC-MS grade	Supelco	1.00029
Methanol, LC-MS grade	Supelco	1.06035
Formic acid	Sigma-Aldrich	5330020050
2,6-Di-tert-butyl-4-methylphenol (BHT)	Sigma-Aldrich	B1378
Phosphate-buffered saline (PBS)	Thermo Fisher Scientific	10010023
Trypsin-EDTA (0.25%)	Thermo Fisher Scientific	25200072
DMEM	Thermo Fisher Scientific	11965092
Lipopolysaccharide (LPS, *E. coli* O111:B4)	Merck	LPS25

**Experimental models: Cell lines**		

Mouse: RAW 264.7	ATCC	TIB-71

**Experimental models: Organisms/strains**		

Rat: Human *SOD1*-G93A transgenic rat, male, 70 and 120 days old	Taconic	N/A

**Software and algorithms**		

Analyst software (v1.7.1)	SCIEX	N/A
PeakView software (v2.2)	SCIEX	N/A
MultiQuant software (v3.0.3)	SCIEX	N/A
R	R Foundation for Statistical Computing, Vienna, Austria	https://www.R-project.org/
RStudio	Posit	https://posit.co/products/open-source/rstudio/
*staRoxy* (v0.2.0)	This paper	https://doi.org/10.5281/zenodo.20126956

**Other**		

Mass spectrometer	SCIEX	TripleTOF 6600
UHPLC system	Shimadzu	NexeraX2
BEH UPLC C18 column, 1.7 μm, 2.1 × 100 mm	Waters	176000864
Thermomixer	Eppendorf	Thermomixer C
Centrifuge	Thermo Fisher Scientific	Sorvall Legend X1R
Sample concentrator	Techne	FSC400D
SPE vacuum manifold	Supelco	11-100-3048
Vacuum pump	KNF Neuberger	N/A
Strata-X reversed-phase SPE columns	Phenomenex	8B–S100-UBJ
Disposable liners for Visiprep DL manifolds	Supelco	570590
BD Vacutainer EDTA tubes (16 x 100 mm, 10 mL)	Becton Dickinson	366643
5 mL microcentrifuge tube	Axygen	MCT-500-C
2 mL microcentrifuge tube	Axygen	MCT-200-C-BRA
2 mL amber screw vial	Agilent	5190-9063
Pre-slit screw cap septum	Waters	186000305
Screw cap	Waters	186000274
250 μL glass insert	Agilent	5183-2085
0.2–10 μL pipette tip	Axygen	T-300-C-BRA
200 μL pipette tip	Axygen	T-200-Y
100-1000 μL pipette tip	Axygen	T-1000-B-BRA

## Materials and equipment

The instrumentation used in this protocol for targeted analysis of oxylipins consists of a TripleTOF 6600 mass spectrometer (SCIEX, Concord, ON, Canada) coupled to a Nexera UHPLC system (Shimadzu, Kyoto, Japan). The TripleTOF 6600 is a quadrupole time-of-flight (QTOF) instrument equipped with an electrospray ionization (ESI) source, capable of high-resolution data acquisition. Chromatographic separation of oxylipin isomers was achieved using a Waters BEH UPLC C18 column (1.7 μm, 2.1 mm × 100 mm).

This protocol is optimized for this specific instrumental setup. Changing the instruments will require parameter adjustments. Specific adaptations for a Q-Exactive Plus (Thermo) or an Orbitrap Exploris 240 (Thermo) mass spectrometer coupled to a Vanquish Flex ultra-high-performance liquid chromatograph (Thermo) are also described in this protocol.

## Step-by-step method details

### Preparation of the internal standard mix

**Timing: 30 min**

This section describes the preparation of the internal standard (IS) mix from commercially available deuterated oxylipin standards.

1. Transfer the contents of the 12 deuterated oxylipin standards according to Table 1 into a clean 50 mL glass tube.

**Table 1:** Internal standards mix

No.	Reagent or Resource	Source	Catalog number	Transferred Mass (μg)	IS mix conc. (ng/100 μL)
1	13(*S*)-HODE-d4	Cayman	338610	25	50
2	(±)12(13)-DiHOME-d4	Cayman	10009994	25	50
3	15-deoxy-Δ^12,14^-Prostaglandin J_2_-d4	Cayman	318570	25	50
4	5-OxoETE-d7	Cayman	334250	25	50
5	5(*S*)-HETE-d8	Cayman	334230	25	50
6	(±)11(12)-EET-d11	Cayman	10006413	25	50
7	Prostaglandin A_2_-d4	Cayman	310210	50	100
8	9-Nitrooleate-d17	Cayman	10557	50	100
9	Prostaglandin E_2_-d4	Cayman	314010	50	100
10	Lipoxin A_4_-d5	Cayman	10007737	25	50
11	Thromboxane B_2_-d4	Cayman	319030	25	50
12	Resolvin D_1_-d5	Cayman	11182	25	50
2. Add methanol to the tube to a final volume of 50 mL. Mix well.
3. Divide the internal standard mixture into 5 mL aliquots using clean glass tubes.

***Note:*** Preparing aliquots minimizes degradation caused by repeated freeze-thaw cycles and reduces the risk of contamination of the internal standard mix.

4. Store the IS Mix at −20°C.

### Preparation of oxylipin stock solutions

**Timing: 1 day**

This section describes the preparation of the oxylipin stock solutions derived from both commercially available and in-house synthesized compounds. These stock solutions are further diluted to perform fragmentation studies to identify species-specific fragmentation patterns and to construct the calibration curves for quantitative analysis.

5. Prepare the individual stock solutions of each oxylipin in methanol according to the specifications in Table S2.

**CRITICAL:** Oxylipins supplied in methyl acetate may evaporate during transport or long-term storage, leading to concentration changes and potential degradation. After opening, evaporate the methyl acetate and reconstitute the compound in equal volume of methanol to ensure stability and the correct concentration.

6. Prepare a 1 μg/mL solution of each oxylipin for MS parameter optimization according to the dilutions specified in Table S2.
7. Store both the oxylipin stock solutions and the 1 μg/mL solutions at −80°C.

### Preparation of oxylipin calibration curve standards

**Timing: 3 h**

This section describes the preparation of the oxylipin calibration curve using the previously prepared oxylipin stock solutions.

8. Transfer the indicated volumes from each oxylipin stock solution, according to the specifications in Table S3, into a clean 5 mL glass tube for the preparation of the oxylipin mix solution.
9. Completely dry the mixture under a stream of nitrogen gas.
10. Reconstitute the dried residue in 1 mL of methanol.
11. Vortex for 1 min.
12. To prepare calibration standards A to N, first transfer the appropriate volume of oxylipin mix solution into clean 2 mL glass vials, as indicated in Table 2 (Step 1).

**Table 2:** Oxylipin calibration curve

Calibration standard	Step 1	Step 2	Step 3	Final volume (μL)
	Transferred from oxylipin mix solution (μL)	Action	Added IS mix (μL)	
A	200	Dry under N_2_ gas	200	200
B	150	Dry under N_2_ gas	200	200
C	100	Dry under N_2_ gas	200	200
D	50	Dry under N_2_ gas	200	200
E	20	Dry under N_2_ gas	200	200
F	15	Dry under N_2_ gas	200	200
G	10	Dry under N_2_ gas	200	200
H	5	Dry under N_2_ gas	200	200
I★	10	Dry under N_2_ gas	1000	1000

★ **Use this solution for the preparation of calibration standards J-N**				

**Calibration Standard**	**Transferred from Calibration Standard I** ★ **(μL)**	**Action**	**Added IS Mix (μL)**	**Final Volume (μL)**

J	150	Dry under N_2_ gas	200	200
K	100	Dry under N_2_ gas	200	200
L	50	Dry under N_2_ gas	200	200
M	20	Dry under N_2_ gas	200	200
N	10	Dry under N_2_ gas	200	200
13. Dry all solutions under nitrogen gas, as indicated in Table 2 (Step 2).
14. Reconstitute each dried solution by adding the indicated volume of IS Mix, as indicated in Table 2 (Step 3).

***Note:*** Oxylipins are quantified using a calibration curve based on the ratio between the peak area of each oxylipin, which varies according to the concentration of the calibration standard, and the peak area of its corresponding deuterated internal standard, which is present at the same concentration in all calibration standard solutions. Drying the solvent in **Step 13** followed by its reconstitution with the same volume of IS Mix minimizes pipetting errors and ensures identical concentrations of deuterated compounds across all calibration standard solutions.

15. Vortex each calibration standard for 30 s.
16. Transfer the solution into a 250 μL glass insert.
17. Store the Calibration Curve at −20°C.

***Note:***Table S3 provides the final concentration of each oxylipin in Calibration Standards A to N, as well as their corresponding mass contained in 5 μL (volume injected in the mass spectrometer during oxylipin analysis).

**CRITICAL:** For injection into the MS system, use Waters pre-slit screw caps (186000305). These caps prevent polymer contamination that may occur when standard caps are pierced during analysis. If LC-MS analysis is not performed immediately, store the samples using Waters screw cap with bonded PTFE/Silicone septa (186000274).

### Oxylipin extraction from biological samples

**Timing: 6 h**

The following section outlines the oxylipin extraction procedure, which consists of an initial liquid-liquid extraction step followed by a solid-phase extraction (SPE).

18. Perform liquid-liquid extraction.
  a. Prepare triplicate blank samples.
    **CRITICAL:** Blank samples are required to monitor background contaminants that may arise during sample processing or analysis (e.g., solvents, plastics, SPE cartridges, handling) rather than from the biological matrix, and to prevent false positives during MS analysis. The blank must be processed through the entire workflow exactly as the regular samples. Prepare the blank by replacing the sample with the same volume of methanol used for protein precipitation in **Step 18.c**.
  b. Transfer 500 μL of sample into a 5 mL tube.
  c. Add 2 mL of cold methanol containing 10 μM 2,6-di-tert-butyl-4-methylphenol (BHT) to each sample.
  d. Add 100 μL of the IS Mix to each sample.
  e. Vortex samples for 30 s.
  f. Incubate the samples in an ice-cold bath for 10 min.
  g. Centrifuge samples at 2,000 × *g* for 10 min at 4°C.
  h. Collect the supernatant by carefully pouring the liquid phase into another tube. Take care not to disturb the cell pellet.
  i. Concentrate the samples under a stream of nitrogen until the volume is reduced to approximately 400–500 μL.
    **CRITICAL:** Simultaneous concentration of multiple samples under nitrogen can be difficult to control, as some samples may concentrate faster than others. Nevertheless, two factors are critical at this stage: (1) Even if the samples do not reach exactly the same final volume, the remaining volume should not exceed 500 μL. Because methanol evaporates more rapidly than water, concentrating the samples to this volume range substantially reduces the methanol content and is therefore not expected to compromise SPE retention under the conditions described in **Step 19**. (2) Complete drying of the samples should be avoided, as this may increase the risk of oxylipin degradation.
19. Perform solid phase extraction (SPE).
  a. Insert a valve liner into each port of the SPE vacuum manifold. Be careful not to bend the valve liner, as this may slow down or even interrupt the flow of the solvent out of the SPE cartridge.
  b. Lock the Strata-X SPE cartridges into the cartridge ports of the vacuum manifold.
  c. Condition each Strata-X SPE cartridge with 3 mL of methanol.
  d. Equilibrate each SPE cartridge with 3 mL of ultrapure water, such as Milli-Q water.
  e. Apply samples to the SPE cartridges.
  f. Wash each SPE cartridge with 1 mL of 5% methanol in water.
  g. Elute the oxylipins with 1 mL of methanol and collect the eluate in a tube or glass vial.
    ***Note:*** A single washing and elution cycle is sufficient for oxylipin extraction.
    ***Note:*** Immediately after adding methanol, residual water may remain in the cartridge outlet. Discard approximately the first 5 drops of eluate before collecting the methanol fraction containing the oxylipins. The presence of residual water in the collected eluate may slow solvent evaporation in **Step 19.h**. If residual water is collected, dry the samples through multiple evaporation cycles, adding methanol between each round.
    ***Note:*** Alternatively, methanol can be evaporated using a low-temperature vacuum concentrator (e.g. SpeedVac), provided that the temperature is maintained at or below 15°C and the concentration time does not exceed 12 h, to minimize the risk of oxylipin degradation.
  h. Dry the samples under a stream of nitrogen.
  i. Reconstitute the dried samples in 100 μL of methanol.
  j. From each sample, take 10 μL and combine them to prepare a pooled quality control (QC) sample.
    **CRITICAL:** The pooled QC sample must be representative of all samples in the batch in terms of lipid composition and concentration range. Therefore, prepare the QC only after **Step** 19.i**,** after all individual samples have undergone the same processing steps. Any subsequent modification applied to the individual samples must also be applied to the QC sample.
  k. If necessary, transfer each sample to a glass insert.
  l. Store the samples at −20°C until LC-MS analysis.
    **CRITICAL:** For injection into the MS system, use Waters pre-slit screw caps (186000305). These caps prevent polymer contamination that may occur when standard caps are pierced during analysis. If LC-MS analysis is not performed immediately after extraction, store the samples using Waters screw cap with bonded PTFE/Silicone septa (186000274).

### Solid-phase extraction using positive pressure processor (Extrahera automated system)

**Timing: 30 min**

This section describes an alternative step-by-step protocol for the extraction of oxylipins from plasma samples using the Extrahera Automated System (Biotage, Uppsala, Sweden), as published in Kolmert et al.,^2^ adapted to 96-well format. To perform oxylipin extraction using this alternative method, follow the liquid-liquid extraction procedure up to **Step 18.i**, then proceed with this protocol (**Steps 20–28**).

20. Set-up the Extrahera system according to the following parameters by Creating or Editing the desired method to match the following:
  a. Under the category of “Sample”, set the sample to match the total volume in the sample plate and select 1000 μL Biotage pipet tips.
  b. Under “Conditioning”, set the pressure to 1.5 bar for 60 s (1 cycle) and set the volume to 1000 μL of methanol (MeOH).
  c. Under “Equilibration”, set the pressure to 2 bar for 60 s (1 cycle) and set the volume to 1000 μL of Milli-Q water.
  d. Under “Load”, set the sample volume to match the volume of the samples. Set the pressure to 1.5 bar for 170 s.
  e. Under “Wash”, set the pressure to 2 bar for 120 s and set the volume to 1800 μL of 10% methanol in water. Use Plate Dry 80 s.
  f. Under “Elution”, set the volume to 1800 μL of methanol and place the collection plate in carousel position B. Set the pressure gradient to use 1.2 bar for 120 s, then 2.0 bar for 60 s. Use collection plate position B.
  g. For steps 1–5, set the collection position to rack D (waste).

***Note:*** Different SPE methods can be set up using this instrument depending on the extraction matrix and analytical goals. The system is also compatible with extraction in a 3 mL cartridge format. Calibration is required for each setting to ensure alignment of sample rows/columns and pipette tips, as well as to confirm that pipette tips aspirate the entire sample volume before transferring it to the SPE plate (see troubleshooting 8 for further details).

21. Turn on the Extrahera system and fill Lines 1–3 with fresh solvents as follows. Line 1: 100% methanol; Line 2: water; Line 3: methanol:water (10:90 v/v).
22. Prime the lines 3 x 15 mL, discarding the volume each time.
23. Place the waste plate in bottom position D of the bottom rack.
24. Place the SPE plate (Express Evolute ABN, 30 mg, Biotage, 600-0030-PX01, or Oasis HLB, 30 mg, Waters, SKU: WAT058951) in position 3 of the upper rack.
25. Place the sample collection plate (2 mL round-bottom Collection plate, Biotage) in position B of the lower rack.
26. Transfer the study samples into the sample preparation plate (96-well, 2 mL, Waters SKU: 186002482).
27. Place the sample preparation plate (Waters, 96-well, 2 mL, SKU: WAT058958) in position 4 of the upper rack.
28. Turn on the vacuum valve and start the method. Wait a few minutes to confirm that the method is running properly. If a problem occurs, the method will not initiate.

**CRITICAL:** Ensure that collection plates are placed in the correct orientation. The Extrahera system places the collection plates into a rotating carousel that allows for multiple fraction collections. Incorrect positioning can result in samples being collected in the wrong wells, leading to mislabeling.

### Preparation of the solvents

**Timing: 1 h**

This section describes the preparation of the mobile phase for UHPLC-MS analysis.

29. Prepare mobile phase A and B by mixing the solvents and additives in the proportions shown in Table 3 using clean amber glass bottles.

**Table 3:** Mobile phase compositions

Reagent	Volume
**Solvent A: water/acetonitrile/acetic acid (70:30:0.02, v/v/v)**	

Water	700 mL
Acetonitrile	300 mL
Acetic acid	200 μL

**Solvent B: Acetonitrile/isopropanol/acetic acid (50:50:0.02, v/v/v)**	

Acetonitrile	500 mL
Isopropanol	500 mL
Acetic acid	200 μL

**Needle Wash: Methanol/isopropanol (50:50, v/v)**	

Methanol	500 mL
Isopropanol	500 mL

***Note:*** Wash all glassware thoroughly with methanol to remove any residues or contaminants originating from cleaning detergents. For both solvents and additives, avoid pipetting directly from the original recipient to avoid contamination. Instead, withdraw the required volume from an aliquot. In particular, for additives that may degrade plastic pipette tips (e.g.; acetic acid), transfer the solution using a clean Hamilton syringe to minimize polymer contamination from plastic materials.

**CRITICAL:** Although Table 3 shows the standard compositions for preparation of 1 L of each solution, it is recommended to prepare only the volume required for the analysis. Storing mobile phases for extended periods (longer than 15 days) can lead to solvent deterioration, resulting in elevated baseline noise and polymer contamination. To minimize degradation, store the solvents in a refrigerated environment, inside tightly sealed amber glass bottles to protect from light.

30. Degas the solutions for 20 min before use.

### UHPLC-MS parameter optimization and setup

**Timing: Variable**

This section describes the procedures for determining the fragmentation pattern of each oxylipin and the optimization of MS parameters to maximize signal intensity and support selective and confident compound identification.

***Note:****Targeted* LC-MS methods, such as the one described in this protocol, rely on the acquisition of MS/MS spectra from predefined precursor ions. In this approach, each oxylipin is identified by its precursor ion, retention time, and species-specific product ions observed in the acquired MS/MS spectrum. Unlike Multiple Reaction Monitoring (MRM) in triple quadrupole instruments, Q-TOF-based analysis does not monitor predefined precursor-to-product ion transitions during acquisition; instead, diagnostic product ions are selected during data processing.

31. Perform theoretical fragmentation of individual oxylipins using predictive MS/MS tools or databases (e.g., PeakView, LIPID MAPS, MassBank Europe, MassBank of North America - MoNA) and compare the generated fragment ions across species to select specific fragments for identification.

***Note:*** Although *in silico* fragmentation can serve as a guide for specific fragment selection, any theoretical prediction must always be confirmed by experimental data.

32. Optimize MS parameters by acquiring CE and DP ramps through direct infusion of the 1 μg/mL oxylipin solutions prepared in **Step 6.** The optimal values are those that produce the specific fragments with the highest intensity.

***Note:*** For the SCIEX TripleTOF 6600, the optimized CE and DP values for all 125 oxylipins monitored in this method are listed in Table S4. The corresponding parameters for the deuterated species included in the IS Mix are provided in Table S5. Both tables also report the specific fragment ion used for identification of each compound.

33. Adjust the source parameter of the SCIEX TripleTOF 6600 according to Table 4;

**Table 4:** MS parameters (TripleTOF 6600, SCIEX)

Source	Value
Mode	Negative electrospray ionization
IonSpray voltage	−4.5 kV
Declustering potential (DP)	−80 V
Curtain gas (CUR)	25 psi
Nebulizer gas (GS1)	50 psi
Heater gas (GS2)	50 psi
Interface heater	500°C
Collision energy (CE)	Individually optimized

**Acquisition**	**Value**

Scan range (m/z)	200-1000 Da
Acquisition cycle time	560 ms
Acquisition time (MS1)	100 ms
Acquisition time (MS2)	10 ms
34. Set the Nexera UHPLC system using the gradient program according to Table 5.

**Table 5:** LC parameters (Nexera, Shimadzu)

Gradient program	
**Time (min)**	**%B**
0.0	0.1
3.5	40
6.0	75
6.5	99
10.5	99
11	0.1
15	0.1

**Chromatographic parameters**	

Column	Waters BEH UPLC C18 column, 1.7 μm, 2.1 × 100 mm
Flow rate	0.5 mL/min
Column temperature	35°C
Injection volume	5 μL

***Note:***Tables S4 and S5 provide expected retention times (RT) for monitored oxylipins and deuterated species under the chromatographic conditions described in Table 5.

### Parameter optimization and setup: Q Exactive Plus mass spectrometer coupled to a Vanquish Flex UHPLC system

**Timing: Variable**

The following steps describe an alternative analytical setup using a Q-Exactive Plus mass spectrometer (Thermo) interfaced with a Vanquish Flex ultra-high-performance liquid chromatograph (UHPLC) system (Thermo). For further information on this alternative setup, please refer to Yapici et al.^3^

35. Set up the resolving power of the Q-Exactive Plus instrument (Thermo) to 17,500 at *m/z* 200.

***Note:*** This configuration will allow the collection of more spectra and monitoring of a higher number of precursor ions in a single run.

36. Adjust fragmentation parameters according to Table S6.

***Note:*** Q-Exactive Plus instruments employ higher-energy collisional dissociation (HCD) for fragmentation analysis, whereas TripleTOF 6600 uses beam-type collision-induced dissociation (CID). Therefore, the fragmentation spectra of some oxylipins in the Q-Exactive Plus do not match those observed in TripleTOF 6600.

***Note:*** MS data acquisition in the Q-Exactive Plus is performed using parallel reaction monitoring (PRM), which works similarly to the “product ion” acquisition method used in the TripleTOF 6600 instrument. Importantly, PRM not only improves selectivity, which refers to the ability of the method to distinguish the target analytes from interfering matrix components, but also provides information about all the fragments derived from the precursor ions, which can be used for further clarification of the chemical structure of oxylipins.

37. Set up MS parameters according to Table 6.

**Table 6:** MS parameters (Q Exactive Plus, Thermo)

Source	Value
Mode	Negative electrospray ionization
Sheath gas	30 au
Auxiliary gas	10 au
Spray voltage	2.5 kV
Ion transfer temperature	320°C
Auxiliary gas heater temperature	120°C
S-lens RF level	55%
Collision energy (CE)	Individually optimized

**Acquisition**	**Value**

Data acquisition method	Parallel reaction monitoring (PRM)
Resolution	17,500 at m/z 200
Automatic gain control target	5e4 counts
Maximum injection time	50 ms
Isolation window	1.5 *m/z*
38. Set up chromatographic conditions of the Vanquish Flex UHPLC system (Thermo) according to Table 7.

**Table 7:** LC parameters (Vanquish Flex, Thermo)

Gradient program	
**Time (min)**	**%B**
0.0	0.1
3.5	40
6.0	75
6.5	99
10.5	99
11	0.1
15	0.1

**Chromatographic parameters**	

Column	Waters BEH Shield RP18 2.1 × 100 mm; 1.7 μm
Flow rate	0.4 mL/min
Oven temperature	40°C
Mobile phase A	water/acetonitrile/formic acid (70:30:0.02, v/v/v)
Mobile phase B	acetonitrile/isopropanol (70:30, v/v)
Injection volume	4 μL

***Note:*** The Vanquish Flex UHPLC system has a lower maximum operating pressure than the Nexera X2 system (18,850 psi). Therefore, the flow rate and injection volume were adjusted to maintain the system pressure within the recommended operating range. This alternative setup also uses a Waters BEH Shield RP18 column instead of the BEH C18 column. The BEH Shield RP18 stationary phase contains an embedded polar group that provides complementary selectivity to conventional C18 phases and was used to achieve suitable chromatographic separation under the alternative LC conditions.

### Parameter optimization and setup: Orbitrap Exploris 240 mass spectrometer coupled to a Vanquish Flex UHPLC system

**Timing: Variable**

The following steps describe an alternative analytical setup using a reduced oxylipin panel adapted for an Orbitrap Exploris 240 (Thermo) coupled to a Vanquish Flex UHPLC system (Thermo).

39. Set up chromatographic conditions of the Vanquish Flex UHPLC system (Thermo) according to Table 7.
40. Set the ionization and acquisition parameters for the Orbitrap Exploris 240 system using the optimized conditions summarized in Table 8.

**Table 8:** MS parameters (Orbitrap Exploris 240, Thermo)

Source	Value
Mode	Negative electrospray ionization (H-ESI)
Sheath gas	50 au
Auxiliary gas	10 au
Spray voltage	2.5 kV
Ion transfer temperature	325°C
Auxiliary gas heater temperature	325°C
S-lens RF level	70%
Collision energy (CE)	Individually optimized

**Acquisition**	**Value**

Data acquisition method	Data-dependent MS^2^ acquisition
Resolution	17,500 at *m/z* 200
Scan range	70–400 *m/z*
Automatic gain control target	1e4 counts
Maximum injection time	50 ms
Isolation window	1 *m/z*
41. Adjust optimal fragmentation values according to Table S7.

**CRITICAL:** For this system configuration, perform mass calibration online at the beginning of each run using the EASY-IC internal calibration system in run-start mode to ensure high mass accuracy.

### UHPLC-MS analysis

**Timing: Variable (15 min per sample)**

This section describes the detailed procedure to perform the analysis of both the calibration curve and the extracted samples by UHPLC-MS.

42. Prepare the LC-MS system by purging the solvents through the LC system and equilibrate the column.
43. Verify that the mass spectrometer is operating in the correct ionization mode (negative) and is properly calibrated.
44. Perform the analysis of the calibration curve.
  a. Inject 5 μL of each calibration standard in quintuplicate (5x) onto the LC-MS system.

**CRITICAL:** Injection of the calibration curve should proceed from the lowest to the highest concentration. Add randomized quintuplicates of blank, quality control (QC), a calibration standard, and IS Mix throughout the sequence to check for carryover and reproducibility. Injection of a calibration standard facilitates oxylipin identification by enabling comparison of retention times, which may vary slightly, particularly for more polar oxylipins. The use of low-concentration calibration standards (I - N), selected from within the detection limits of the instrument, allows qualitative evaluation of the consistency of oxylipin identification performance across analytical batches.

45. Perform the analysis of the extracted samples.
  a. Inject 5 μL of each sample onto the LC-MS system.

**CRITICAL:** Randomize the injection sequence for study samples. Include blank and QC injections throughout the sequence to monitor carryover and reproducibility. Finish the injection sequence with a methanol blank injection to assess residual carryover and background contamination.

### Calibration curve construction

**Timing: 3–4 days**

This section describes the construction of a calibration curve for oxylipin quantification using MultiQuant software (v3.0.3).

46. Create a quantification method in MultiQuant (v3.0.3) by adding the precursor and product ions and retention times for each monitored oxylipin and its deuterated compound in IS Mix.
47. Import the MS data files corresponding to the calibration curve into MultiQuant (v3.0.3) and select the previously created quantification method.
48. In the *Sample Type* column, change the designation from *Unknown* to *Standard* for all samples belonging to the calibration curve.
49. In the *Actual Concentration* column, enter the appropriate concentration (pg/5 μL) for each calibration standard as listed in Table S3.
50. Integrate the chromatographic peaks corresponding to each monitored oxylipin and deuterated internal standard across all samples.

***Note:*** Some oxylipin standards can display more than one chromatographic peak due to isomerism or intrinsic properties. For easier identification, consult Data S1 for peak integration references for each oxylipin. Note that retention times may vary slightly between batches as a result of minor changes in the mobile phase or column cleaning between runs. However, as long as the compound is not degraded, the relative peak pattern will remain consistent. Use these characteristics to select the correct peak for integration.

51. For the curve fitting, apply a 1/x or 1/x^2^ weighting factor.

**CRITICAL:** This weighting approach assigns greater importance to lower-concentration calibration points. Because oxylipins are typically present at low abundance, most biological samples fall within the lower concentration range of the calibration curve, making this weighting scheme more appropriate for accurate quantification.

52. Adjust each oxylipin calibration curve by discarding outliers according to the criteria summarized in Table 9, adapted from Schebb et al.^8^

**Table 9:** Calibration curve parameters

Parameter	Definition	Determination/Acceptance criteria
Signal-to-noise ratio (S/N)	Measure of how clearly an analyte peak rises above baseline noise	Calculated from unsmoothed chromatograms as the height of the analyte peak above the mean baseline divided by the peak-to-valley baseline noise
Accuracy %^a^	Closeness of the calculated concentration to the actual concentration, as calculated using the regression function generated from the calibration standards	Calculated as: $\frac{Measured\;concentration}{Actual\;concentration}\times 100\%$ Acceptable values are 100 ± 20% at LLOQ and 100 ± 15% for other calibration points
Coefficient of variation (CV %)	Measure of the precision of replicate injections	Calculated as: $\frac{Standard\;deviation}{Mean}\times 100\%$ Acceptable if ≤ 20%
Limit of Detection (LOD)	Lowest injected amount at which the analyte can be reliably detected	Defined as the lowest injected amount for which at least three repeated injections of primary standards yield S/N ≥ 3.0
Lower Limit of Quantification (LLOQ)	Lowest injected amount at which the analyte can be quantified with acceptable accuracy and precision	Defined as the lowest injected amount for which S/N ≥ 5; accuracy of 100% ±20%; and CV ≤ 20% in repeated injections of primary standards

a Various software programs, including MultiQuant (v3.0.3), use the term “accuracy” as a synonym for “calibrator recovery” or “relative mean error (RME)”, which has led to its widespread use in the field. However, it should not be confused with “analyte recovery”, which refers to the efficiency of analyte extraction and ranges from 0 to 100%.
53. Save the calibration curve as a.*qsession* file.

***Note:*** Schebb et al. 2025^8^ provides a complete set of technical recommendations for the analysis of oxylipins by LC-MS. For detailed guidance on the determination of each criterion, as well as other good practices for reporting oxylipins in biological samples, please refer to these guidelines.

### Oxylipin quantification in biological samples

**Timing: Variable (depending on the number of samples and operator’s experience)**

This section describes the quantification of oxylipin in plasma samples using MultiQuant (v3.0.3).

54. Import the MS data files corresponding to the samples into the.*qsession* file containing the calibration curve prepared in the previous section.
55. In the *Sample Type* column, verify that the designation *Unknown* is applied to all samples.
56. Integrate the chromatographic peaks corresponding to each monitored oxylipin and deuterated internal standard across all samples.
57. The *Calculated Concentration* column displays the concentration determined from the calibration curves for the injected sample volume (pg/5 μL).
58. Perform the appropriate calculations to determine the amount of oxylipin per mL of sample.

***Note:*** Take into account that 500 μL of sample was initially used. For plasma and culture medium, final concentration should be expressed as concentration of oxylipin per volume of sample (e.g., mL, μL).

59. For cell pellet samples, perform the appropriate normalization calculations.

***Note:*** Final concentration should be expressed as the concentration of oxylipin per number of cells or per amount of protein (e.g., mg, μg, ng).

60. Perform the data analysis using *staRoxy* (see below).

### Data analysis using *staRoxy*

**Timing: 1 day**

*staRoxy* is a dedicated open-source R^9^ package designed to streamline oxylipidomics quantitative data analysis, providing tools for data preprocessing, statistical modeling, annotation, and integrated visualization. This section describes how to use *staRoxy* version 0.2.0 to perform standard oxylipin abundance analysis.

***Note:*** Updated versions of the *staRoxy* package, along with a detailed vignette describing installation, the recommended workflow, data formatting guidelines, as well as in-depth explanation of the statistical analysis performed in this step-by-step guide are available in the package repository (see data and code availability).

***Note:*** Alternatively, users can perform statistical analyses using MetaboAnalyst,^10^ an open-source, web-based platform for metabolomics data analysis, or other widely used software and packages (see *staRoxy* vignette).

61. First, ensure that R^9^ and Rstudio^11^ are installed on your computer. Then, install *staRoxy* :

>if (!require(“BiocManager”, quietly = TRUE))

>   install.packages(“BiocManager”)

>BiocManager::install(“pefjordao/staRoxy”)

62. The *staRoxy* package requires two input files: (1) an oxylipid abundance data frame, and (2) a metadata data frame containing sample annotations. Format your data and metadata files as described below.
  a. Abundance data frame file:
    i. The first column should be named “oxylipin”, and contain the names of all identified oxylipin species.
    ii. The remaining columns should correspond to individual samples. The first row should contain sample names, and the subsequent rows should contain the quantitative abundance values for each oxylipin.
      ***Note:*** Missing values (*NA*), non-detected values (below the limit of detection, LOD), or non-quantified values (below the limit of quantification, LOQ) should be left empty. Zero values are automatically converted to *NA*.
  b. Metadata data frame file:
    i. The first column should be named “sample” and contain the name of each sample. Sample names must match exactly those provided in the abundance data frame file.
    ii. The second column should be named “group” and contain the experimental group associated with each sample (e.g., WT, Treatment).
    iii. Additional optional columns may be added to specify covariates (e.g., batch, sex, age).
      **CRITICAL:** All column names should be written exactly as specified, without additional spaces of formatting inconsistencies. The current version of *staRoxy* (v0.2.0) is case-sensitive and cannot automatically detect formatting discrepancies.
      **CRITICAL:** Throughout this protocol, we retained the oxylipin nomenclature as adopted by vendor catalog. However, these names may not reflect the most widely accepted standardized nomenclature (IUPAC and LIPID MAPS^4^). *staRoxy* uses standardized nomenclature to generate publication-ready outputs. However, this conversion is not performed automatically. Therefore, users must manually harmonize nomenclature in their datasets prior to analysis. Table S1 provides a harmonized nomenclature reference to facilitate accurate data reporting for the version of *staRoxy* (v0.2.0) used in this protocol. Alternatively, run the *show_database_oxy* function to display the nomenclature for all 125 species currently supported by *staRoxy* (see *staRoxy* vignette).
63. Save the files in any of the supported formats: xlsx, .xls, .csv (comma- or semicolon-separated), .tsv, .txt, or.dat.
64. Open R using an integrated development environment (IDE) such as RStudio,^11^ and load the *staRoxy* package.

>library(staRoxy)

65. Create a *staRoxy* object using the *read_oxy* function. This object will be used in all downstream analyses.

>staRoxy_object <- read_oxy(

> data_path, # path to the abundance data

> meta_path) # path to the metadata

66. Next, filter and transform the abundance data using the *filter_oxy* and *transform_oxy* functions.

>staRoxy_object <- staRoxy_object %>%

> filter_oxy() %>%

> transform_oxy()

67. Retrieve oxylipins detected exclusively in one of the analyzed groups using the *get_exclusives_oxy* function.

>get_exclusives_oxy(staRoxy_object)

68. Perform principal component analysis (PCA) using the *plot_pca_oxy* function.

># You can define group-specific colors for visualization

>my_colors <- c(“WT 70 d” = “#458B00”, “WT 120 d” = “#0D7E88”,

> “ALS 70 d” = “#8B008B”, “ALS 120 d” = “#F65F72”)

>

>plot_pca_oxy(staRoxy_object,

> colors = my_colors)

69. Generate a heatmap with hierarchical clustering using the *plot_heatmap_oxy* function.

>plot_heatmap_oxy(staRoxy_object,

> colors = my_colors)

70. Oxylipin profile analysis can be performed using a PERMANOVA test, with visualization by principal coordinates analysis (PCoA). For this purpose, use the *profile_test* function.

>profile_test(staRoxy_object,

> colors = my_colors)

71. To perform differential abundance analysis and identify differentially abundant oxylipins (DAOs) use the limma^12^ (Linear Models for Microarray Data) framework via the *limma_oxy* function.

>stats <- limma_oxy(staRoxy_object)

72. To inspect the comparisons performed between experimental groups (referred to as “contrasts”), use the *list_contrasts* function.

>list_contrasts(stats)

73. Use the *plot_rank_oxy* function to calculate and visualize log_2_ fold change (log2FC), standard error of the mean (SEM), and adjusted *P*-values using the Benjamini-Hochberg correction.

>plot_rank_oxy(stats,

> contrast = 1, # contrast selected using list_contrasts()

> top_n = 15) # number of oxylipins to display

74. Use the *plot_violin_oxy* function to simultaneously visualize different oxylipin abundance across experimental groups using violin and box plots.

>plot_violin_oxy(staRoxy_object,

> stats,

> oxylipin = “9,10-DiHOME”,

> colors = my_colors,

> show_sig = TRUE,

> sig_y_nudge = 0.4) # adjust the position of the P-value

75. Perform correlation analyses using the *cor_within_oxy* function, which computes correlations between two oxylipins within the same group, or the *cor_between_oxy* function, which compares the abundance of a single oxylipin between two experimental groups.

>cor_between_oxy(staRoxy_object,

> oxylipin = “9,10-DiHOME”,

> group1 = “ALS 120 d”,

> group2 = “ALS 70 d”)

76. Use the *loading_dissimilarity* function to compare oxylipin contributions to the principal component structure between two conditions. This analysis evaluates changes in the contribution of individual oxylipins to the global profile.

>loading_dissimilarity(staRoxy_object,

> group1 = “ALS 120 d”,

> group2 = “ALS 70 d”,

> pc_index = 1)

77. Perform precursor identification using the *classify_oxy* function:

>classify_oxy(staRoxy_object)

***Note:*** Oxylipin names must match exactly those returned by the *show_database_oxy* function. The complete set of all 125 oxylipins described in this protocol must be included in the analysis. Adaptations using expanded or reduced oxylipin panels are also possible (see *staRoxy* vignette).

78. Use the *oxy_ora* function to perform enrichment analysis through over-representation analysis (ORA) based on a hypergeometric test (one-vs-all).

>oxy_ora(staRoxy_object)

## Expected outcomes

This protocol describes how to establish an LC-MS–based methodology for the targeted analysis of oxylipins, encompassing the complete workflow from lipid extraction and instrument analysis to basic data processing. By following this method, users can confidently identify and quantify up to 125 distinct oxylipin species.

This section demonstrates representative results obtained using the protocol described in this paper and analyzed with the *staRoxy* package (v0.2.0). We present data from a previously published blood plasma dataset (Chaves-Filho et al.^1^), as well as original datasets comprising LPS-stimulated macrophage cells and experiments evaluating different harvesting methods.

### Blood plasma data analysis

The dataset from Chaves-Filho et al.^1^ was obtained from blood plasma samples collected from an amyotrophic lateral sclerosis (ALS) rat model (*SOD1*-G93A) at asymptomatic (ALS 70 d, n = 6) and symptomatic stages (ALS 120 d, n = 6), along with their respective age-matched wild type controls (WT 70 d, n = 6, and WT 120 d, n = 6), where d denotates days of age. Blood plasma was collected by cardiac puncture, lipid extraction was conducted following the protocol presented here, and data analysis using *staRoxy* was conducted according to the package vignette.

**Figure 1:**
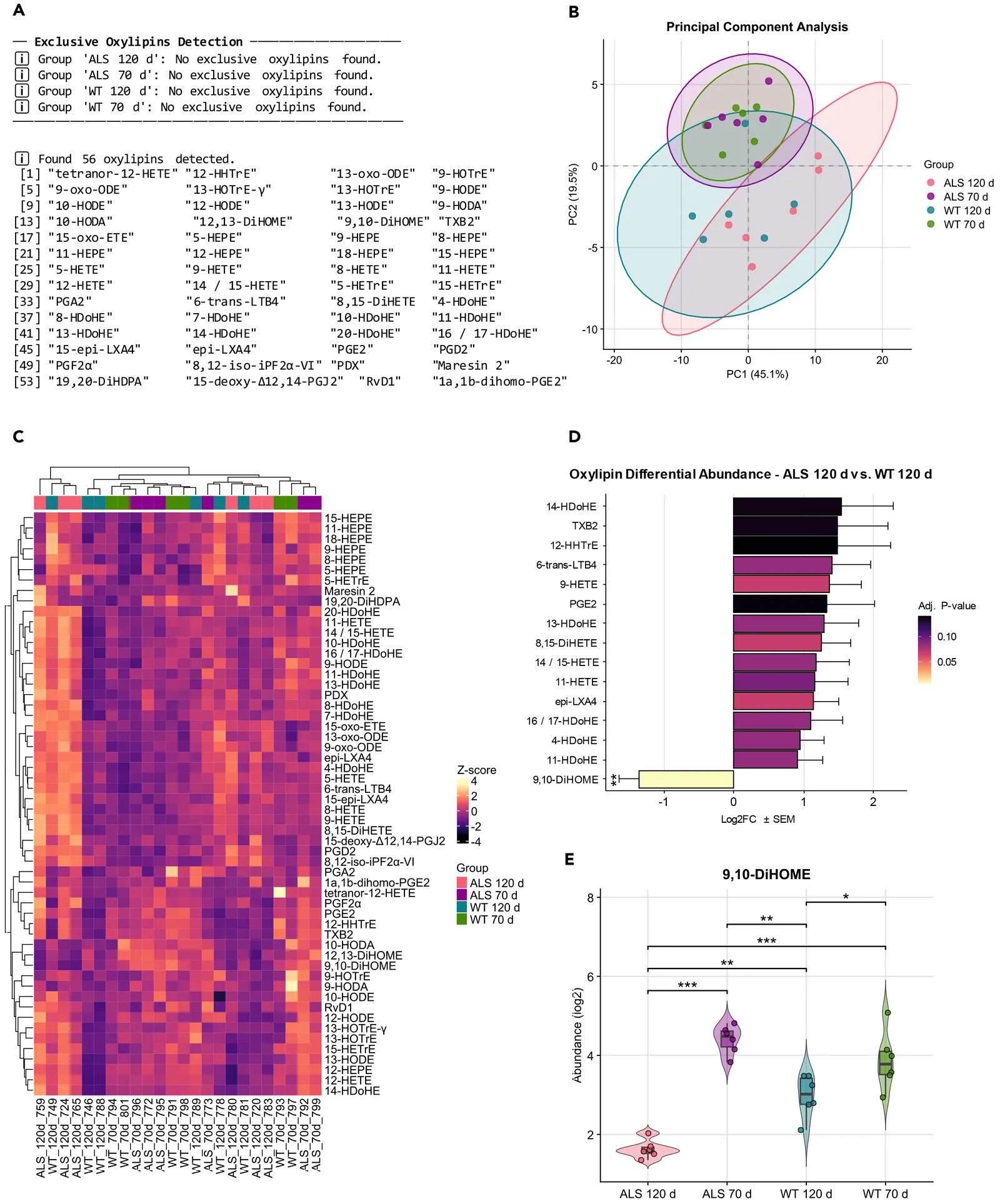
Data analysis using *staRoxy* in blood plasma from *SOD1*-G93A rats (A) *staRoxy* (v0.2.0) output showing detected oxylipins retained after filtering and transformation. (B) Principal component analysis (PCA) plot of amyotrophic lateral sclerosis (ALS) and wild type (WT) groups. The 70 d and 120 d time points correspond to 70-day-old and 120-day-old rats, respectively. (C) Heatmap with hierarchical clustering of Z-score–transformed oxylipin abundance across groups. (D) Rank plot of the top 15 differentially abundant oxylipins based on log_2_ fold change (Log2FC) between ALS 120 d and WT 120 d. Error bars represent SEM. (E) Box-and-violin plot showing the abundance of 9,10-DiHOME across groups. Asterisks denote *P*-values (∗ = *P* < 0.05, ∗∗ = *P* < 0.01, ∗∗∗ = *P* < 0.001).

A total of 56 oxylipins were identified in blood plasma samples, none of which was exclusive to a specific experimental group (Figure 1A). Principal component analysis (PCA) showed substantial overlap between ALS 70 d and WT 70 d, as well as ALS 120 d and WT 120 d, with PC1 and PC2 explaining 45.1% and 19.5% of the variance, respectively (Figure 1B). Consistently, hierarchical clustering revealed limited group separation (Figure 1C).

Differential abundance analysis between ALS 120 d and WT 120 d identified 9,10-DiHOME (Figure 1D) as the only significantly altered oxylipin, being reduced in ALS 120 d (*P*-value < 0.01). Box and violin plot analyses show differences in 9,10-DiHOME abundance across all groups and confirmed that ALS 120 d exhibited the lowest abundance of this oxylipin (Figure 1E).

**Figure 2:**
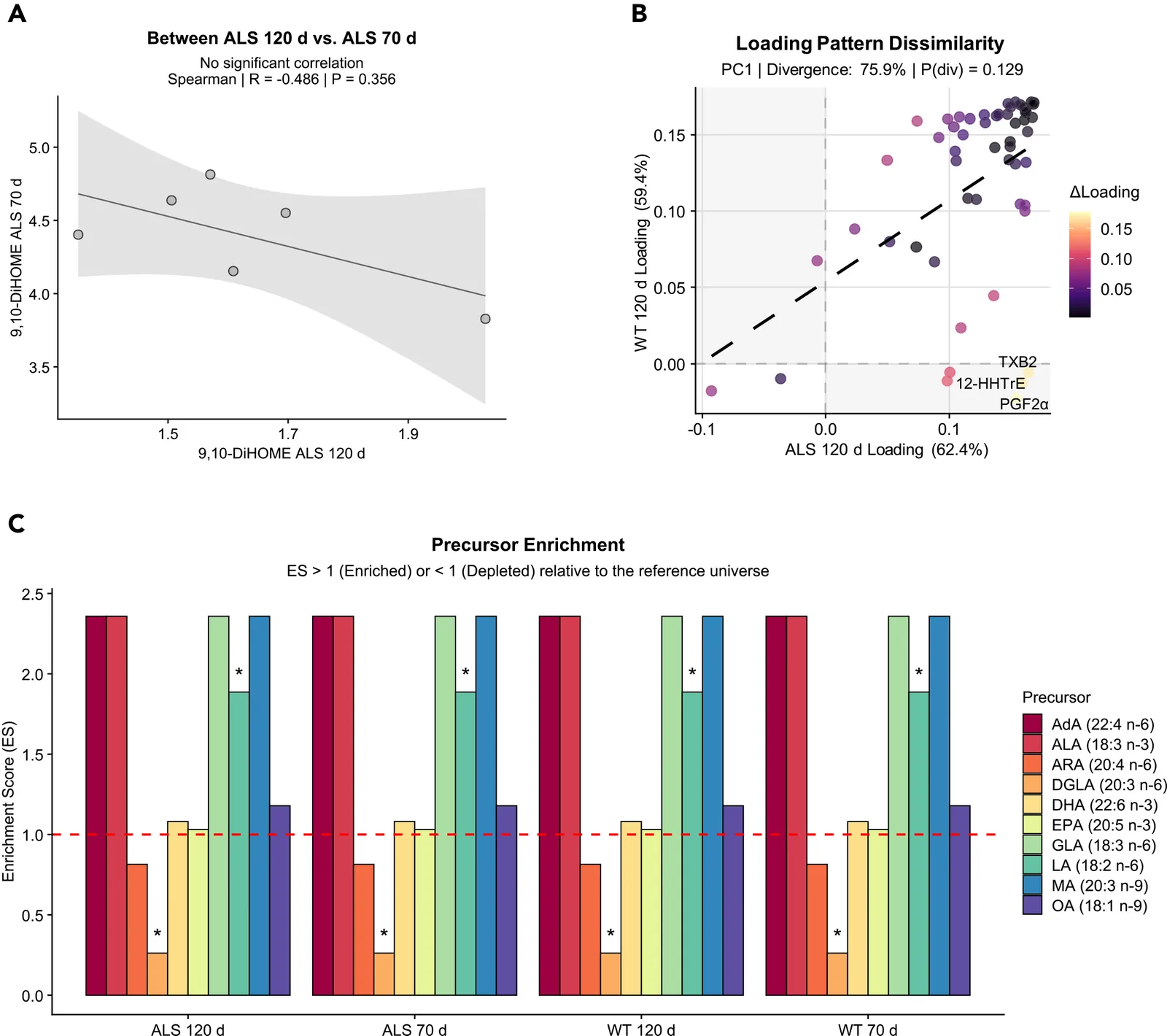
Exploratory data analysis using *staRoxy* in blood plasma from *SOD1*-G93A rats (A) Spearman correlation analysis of 9,10-DiHOME abundance between the ALS 120 d and ALS 70 d groups. (B) Loading pattern dissimilarity analysis plot between oxylipins detected in ALS 120 d and WT 120 d groups. (C) Enrichment plot of oxylipin precursor classes across sample groups. Asterisks denote *P*-values (∗ = *P* < 0.05, ∗∗ = *P* < 0.01).

Correlation analysis of 9,10-DiHOME abundance measured in ALS 120 d and ALS 70 d samples showed a moderate negative correlation (R = −0.486, *P*-value = 0.356). Although not statistically significant, this trend suggests an inverse relationship, whereby higher 9,10-DiHOME levels in ALS 120 d samples are associated with lower levels in ALS 70 d samples (Figure 2A).

Loading pattern dissimilarity analysis between oxylipins detected in ALS 120 d and WT 120 d did not reveal significant differences in global architecture (1 – R^2^ = 75.9%, *P*-value = 0.129) (Figure 2B). However, a qualitative reassessment of individual contributions revealed that key pro-inflammatory mediators, including TXB_2_, 12-HHTrE, and PGF_2α_ shifted from negligible or negative influence (loading <0) to positive contributions. This shift suggests an increased contribution of these oxylipins to the ALS 120 d lipidomic profile, potentially influencing the direction of variance and contributing to the group’s metabolic signature.

Precursor enrichment analysis further indicated that LA (18:2 n-6) is enriched, with species derived from this precursor detected at more than 1.5-fold higher frequency than expected within the background of possible oxylipins (n = 125). In contrast, DGLA (20:3 n-6) was depleted across all groups, suggesting that differences in oxylipin profiles are driven more by changes in abundance than by the diversity of precursor classes in this context (Figure 2C).

### LPS-treated RAW 264.7

RAW 264.7 is a murine macrophage cell line derived from an Abelson leukemia virus–induced tumor in BALB/c mice.^13^ These adherent cells are widely used as a model for inflammation and pharmacological studies.^14^^,^^15^ Its applicability stems from its sensitivity to classical stimuli such as lipopolysaccharide (LPS) and Kdo2-Lipid A (KLA). Activation via Toll-like receptor 4 (TLR4) triggers a signaling cascade that culminates in the expression of pro-inflammatory genes and activation of phospholipases.^16^^,^^17^ This process promotes the release of polyunsaturated fatty acids (PUFAs) from cellular membranes, which serve as precursors for oxylipin synthesis.

Using the protocol described in this paper, we evaluated the oxylipin profile of LPS-treated RAW 264.7 macrophages. Cells were seeded at 1 × 10^6^ cells per well in 6-well plates in DMEM supplemented with GlutaMAX. After 24 h, cells received either fresh medium (control) or LPS (100 ng/mL). After an additional 24 h, supernatant and trypsinized cell pellets were collected and analyzed. Lipid concentrations were normalized to total protein content for pellets (fmol/mg) and volume for supernatants (fmol/mL). These raw abundance values are provided in Tables S8 and S9.

**Figure 3:**
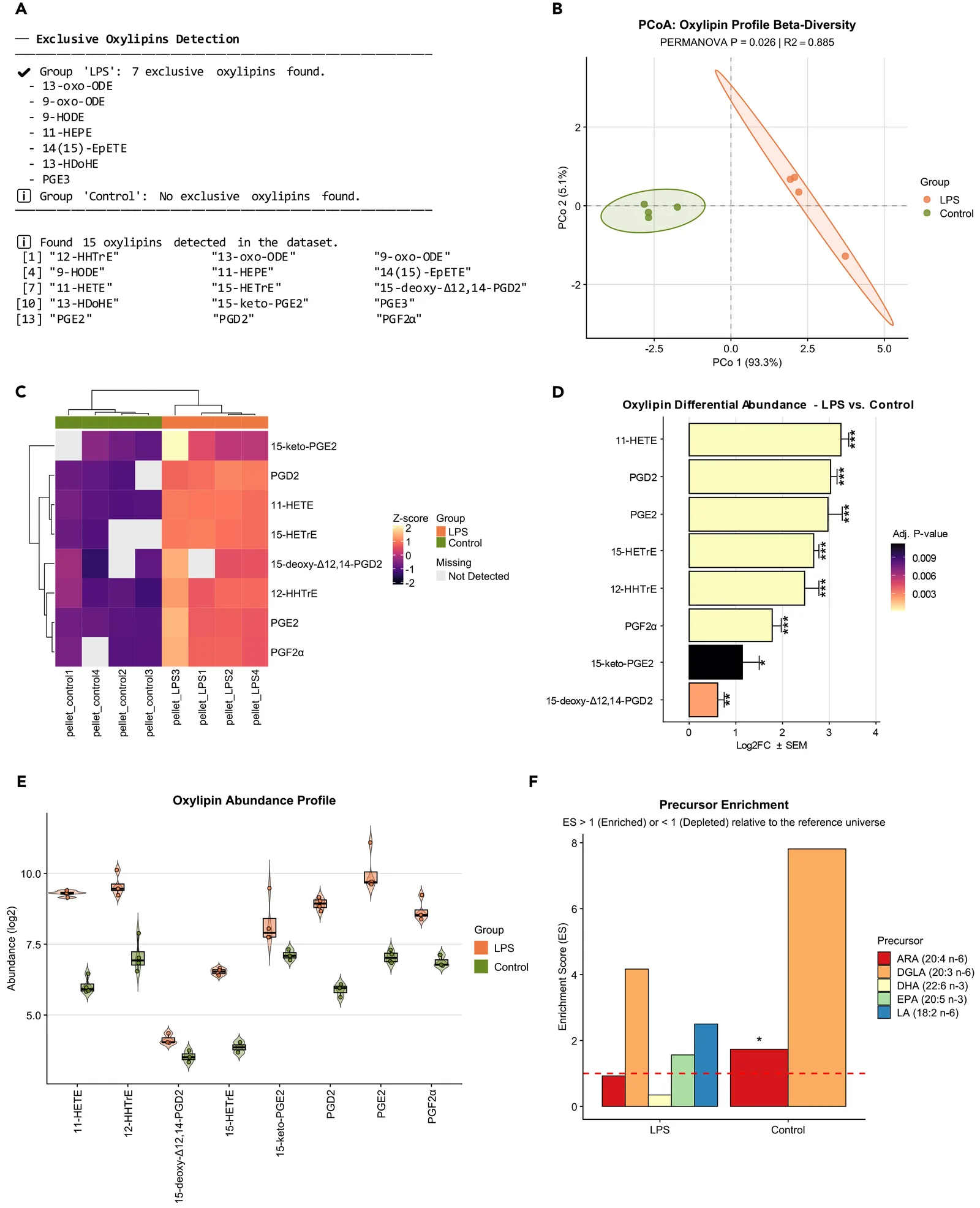
Data analysis using *staRoxy* in cell pellets from LPS-treated RAW 264.7 macrophages (A) *staRoxy* (v0.2.0) output showing detected oxylipins retained after filtering and transformation. (B) Principal coordinate analysis (PCoA) plot of oxylipins detected in LPS (n = 4) and Control (n = 4) groups. (C) Heatmap with hierarchical clustering of Z-score–transformed oxylipin abundance across groups. Gray cells indicate oxylipins not detected. (D) Rank plot of differentially abundant oxylipins based on log_2_ fold change (Log2FC) between LPS and Control groups. Error bars represent SEM. (E) Box-and-violin plots showing differentially abundant oxylipins across groups. (F) Enrichment plot of oxylipin precursor classes across groups. Asterisks denote *P*-values (∗ = *P* < 0.05, ∗∗ = *P* < 0.01, ∗∗∗ = *P* < 0.001).

After filtering and transformation using *staRoxy*, 15 oxylipins were retained in the analysis of cell pellets, 7 of which were exclusive to the LPS group (Figure 3A): 13-oxo-ODE, 9-oxo-ODE, 9-HODE, 11-HEPE, 14(15)-EpETE, 13-HDoHE and PGE_3_. Because statistical analysis in *staRoxy* requires species to be present in both groups, these 7 oxylipins were excluded from downstream analyses.

PCoA revealed a clear separation between the LPS and Control groups, with approximately 90% of the variation in oxylipin profiles explained by group differences (Figure 3B; R^2^ = 0.885, *P*-value = 0.026; PERMANOVA with 10,000 permutations). The heatmap showed strong clustering of samples by treatment, indicating that the LPS group exhibited higher abundance levels for all oxylipins compared to the Control group (Figure 3C). Differential abundance analysis confirmed that all oxylipins were significantly more abundant in the LPS group (Figures 3D and 3E). Finally, enrichment analysis revealed enrichment of arachidonic acid (ARA, 20:4 n-6)-derived oxylipins in the Control group (Figure 3F), with a 1.74-fold higher representation than expected based on the reference background of 125 oxylipins.

**Figure 4:**
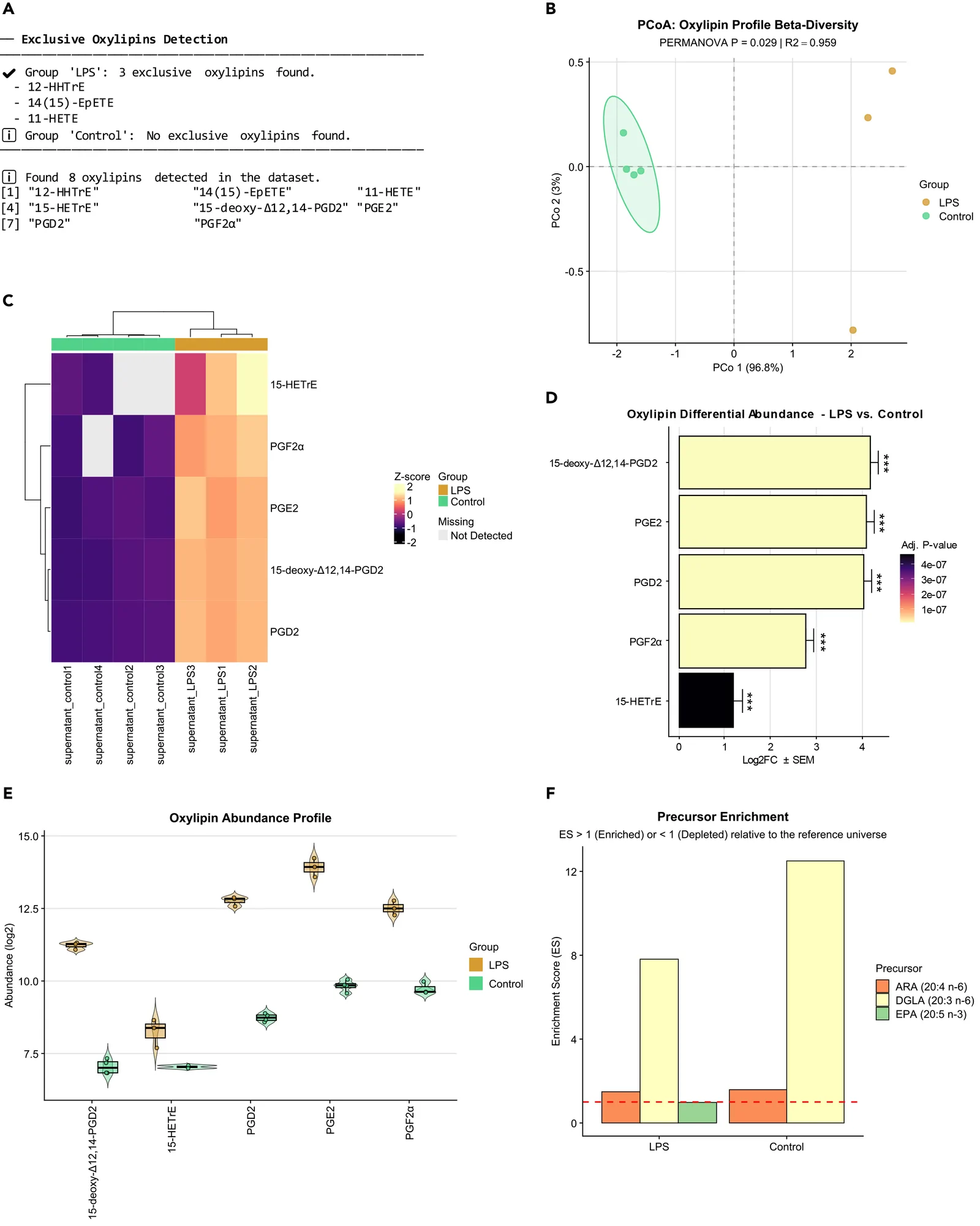
Data analysis using *staRoxy* in supernatants from LPS-treated RAW 264.7 macrophages (A) *staRoxy* (v0.2.0) output showing detected oxylipins retained after filtering and transformation. (B) Principal coordinate analysis (PCoA) plot of oxylipins detected in LPS (n = 3) and Control (n = 4) groups. In *staRoxy*, ellipses are generated only for groups with n ≥ 4; therefore, the LPS group is not represented by an ellipse. (C) Heatmap with hierarchical clustering of Z-score–transformed oxylipin abundance across groups. Gray cells indicate oxylipins not detected. (D) Rank plot of differentially abundant oxylipins based on log_2_ fold change (Log2FC) between LPS and Control groups. Error bars represent SEM. (E) Box and violin plots showing differentially abundant oxylipins across groups. (F) Enrichment plot of oxylipin precursor classes across groups. Asterisks denote *P*-values (∗ = *P* < 0.05, ∗∗ = *P* < 0.01, ∗∗∗ = *P* < 0.001).

The cell supernatant was also analyzed, yielding 8 oxylipins after filtering and transformation. Among these, 12-HHTrE, 14,15-EpETE, and 11-HETE were exclusive to the LPS group (Figure 4A) and were therefore removed from downstream analyses. PCoA showed tight clustering of the Control group, whereas the LPS group displayed one outlier sample (Figure 4B). PC1 accounted for 96.8% of the explained variance, indicating that the primary source of variation in oxylipin profiles is associated with LPS treatment. Consistently, the heatmap showed clear group clustering and indicated higher oxylipin abundance in the LPS group compared to the Control group (Figure 4C). All oxylipins showed significantly higher abundance in the LPS group (Figures 4D and 4E). However, no precursor class showed significant enrichment (Figure 4F).

These findings are consistent with previous reports showing that stimulation of RAW 264.7 macrophages with KLA^16^^,^^18^ or LPS^19^^,^^20^ induces an oxylipin profile primarily derived from arachidonic acid (AA) oxidation via cyclooxygenase (COX), lipoxygenase (LOX), and cytochrome P450 (CYP450) pathways. In particular, PGJ_2_, PGF_2α_, PGD_2_, and PGE_2_, as well as the monohydroxy derivative 11-HETE, have been consistently reported in both cellular and supernatant samples.^16^^,^^17^^,^^18^^,^^19^ Similar eicosanoid profiles have also been observed in other macrophage models, including bone marrow–derived macrophages (BMDMs) and peritoneal macrophages.^21^^,^^22^ The detection of these key mediators in our dataset supports the consistency of our results with established biological responses in this model.

In terms of coverage, our methodology detected 15 oxylipin species in cell pellets and 8 in supernatants. This coverage falls within the range reported in previous studies used for this comparison, including 25 in Norris et al.,^18^ 18 in Lee et al.,^20^ and 7 in Dennis et al.^16^ (see Table S10; Figure S1 for a detailed comparison). Differences in the species detected across methods, including method specific species, likely reflect the variation in methodological scope, as targeted methodologies are limited to predetermined species included in their analytical panels, whereas broader approaches may capture a higher number of species. Overall, these results demonstrate that our method reliably captures the key features of oxylipin production in LPS-stimulated RAW 264.7 macrophages, with performance comparable to previously established methodologies.

## Limitations

Oxylipins are present in very low concentrations in biological samples, which poses a significant analytical challenge. To address this, this protocol requires a relatively large amount of starting material, which may represent a limitation for certain experimental designs, particularly those involving small animals or limited sample volumes. In addition, the sample preparation workflow focuses exclusively on the isolation of free oxylipins. As a result, oxylipins esterified within more complex lipid species are not captured, and the profile obtained represents only a portion of the total oxylipin pool in the sample. When comprehensive profiling is required, hydrolysis-based protocols can be employed prior to analysis.^23^

Beyond limitations in sample preparation, the liquid chromatography conditions described in this protocol do not resolve certain oxylipin stereoisomers, such as the enantiomeric pair 9(S)-HODE and 9(R)-HODE, and the diastereoisomeric pair 6-trans-LTB_4_ and 6-trans-12-epi-LTB_4_. Therefore, when detected using this methodology, these compounds should be reported as unresolved isomeric pairs, as it is not possible to unambiguously differentiate between the individual stereoisomers. When stereoisomer-specific identification is required, complementary approaches based on chiral chromatography or supercritical fluid chromatography may be employed.^24^^,^^25^

Practical considerations related to sample processing should also be taken into account. Solid phase extraction (SPE), as performed in Chaves-Filho et al.,^1^ presents some limitations: (1) the number of samples that can be processed simultaneously is restricted by the number of cartridge ports available in the vacuum manifold (typically 12 to 24 in Supelco systems); (2) solvent flow may be slowed by its viscosity and often varies among cartridges, even when a vacuum pump is used; and (3) the valves for opening and closing each port can be difficult to access once all SPE cartridges are in place. Together, these factors may reduce the method’s throughput and reproducibility. An alternative to this setup is the use of positive pressure processors. These devices enable sample processing in a 96-well plate format, substantially increasing sample throughput. Positive pressure is applied using inert gas, which accelerates solvent flow, reduces processing time, and significantly improves reproducibility compared to vacuum-based manifolds. This protocol provides an alternative to SPE workflows intended to overcome these limitations (see solid extraction using positive pressure processor (Extrahera automated system) - Optional).

Finally, as with most targeted workflows, this protocol relies heavily on commercially available standards for each oxylipin included in the *targeted* method; therefore, the development of such a method can be costly and inherently limited to the available compounds. Although some oxylipins can be synthesized in-house to overcome this limitation, as is the case for some oxylipins in this protocol, this approach depends on specialized expertise.

## Troubleshooting

### Problem 1

Samples dry completely during concentration stage before SPE extraction (related to Step 18).

### Potential solution

If a sample dries completely, it is recommended to reconstitute it in approximately 300 μL of 5% methanol in water. The presence of methanol facilitates oxylipin solubilization, while at this concentration it should not adversely affect SPE extraction.

### Problem 2

Poor recovery of most oxylipins after SPE extraction (related to Step 19).

### Potential solution

Low recovery may be caused by drying of the SPE sorbent, which can compromise retention efficiency, or by an excessively high methanol content during sample loading. To avoid these issues, ensure that the SPE sorbent remains wet throughout the extraction procedure and that the concentration step performed before SPE results in a final sample volume of 500 μL or less, thereby maintaining the methanol content within the recommended range. In addition, when collecting the eluate, avoid discarding more than the 5 drops before collection, as this may result in the loss of oxylipins present in the elution fraction.

### Problem 3

Inconsistent volumes aspirated from pipette tips when transferring samples from the 96-well sample extraction plate to the SPE plate (SPE using Extrahera) (related to Step 20).

### Potential solution

In the Extrahera instrument, navigate to *‘Data administration’ >> ‘Edit sample plate/rack*’ and adjust plate/rack height and pipetting height to better match the dimensions of the sample extraction plate. When using Waters sample plates, default settings based on well volumes (e.g., 2mL, 1mL, 750μL) are available. If these adjustments are not possible, technical support is required.

### Problem 4

No MS signal is detected (related to Step 33).

### Potential solution

Verify instrument tuning, mass calibration, and overall instrument cleanliness. If the issue persists, request support from the instrument manufacturer.

### Problem 5

Pump pressure is abnormally high (related to Step 34).

### Potential solution

Elevated pressure is most commonly caused by clogging within the LC system or the analytical column. First, remove the column and systematically test the flow path of the LC system by pumping solvent through the system under controlled pressure to identify the source of the blockage. Inspect and test the solvent inlet lines, in-line filters, injector, and pump. Once the clogged component is identified, flush it with appropriate solvents: use aqueous (e.g., water) if clogging is suspected to be caused by polar compounds, or organic solvents (e.g., methanol or isopropanol) if apolar compounds are suspected. If no clogging is detected within the LC system, the blockage is likely located in the column. Reinstall the column and flush it with 100% water for at least 4 h. If clogging by apolar compounds is suspected, wash the column with a 50:50 (v/v) mixture of isopropanol and acetonitrile.

### Problem 6

Pump pressure is abnormally low (related to Step 34).

### Potential solution

Low pressure typically indicates leaks within the LC system. Inspect all fittings and connections for improper installation or damage. In addition, column deterioration can result in reduced pressure. If leaks are not detected, replace the column and reassess system pressure.

### Problem 7

Pump pressure is unstable or fluctuates significantly (related to Step 34).

### Potential solution

Pressure fluctuations are commonly caused by air bubbles within the LC system. Degas all solvents thoroughly, remove the column, and flush the LC system with isopropanol to remove trapped air. Air bubbles trapped in components such as pump valves can be difficult to eliminate. In such cases, remove the valve elements and sonicate them in isopropanol before reinstalling.

### Problem 8

Blank samples show unexpected or aberrant signals (related to Step 44).

### Potential solution

Verify carryover by injecting clean methanol blanks. If the signal persists, clean the injection needle using a 50:50 (v/v) mixture of isopropanol and methanol and clean the column as described in the solution for problem 5. If the issue remains, replace the column. Finally, verify the quality of blank samples and evaluate the possibility of cross-contamination between samples.

### Problem 9

Calibration standards or quality control (QC) samples show high variability in retention time and/or signal intensity (related to Step 44).

### Potential solution

Inspect the LC system for air bubbles. If present, resolve them as described in problem 7. Column deterioration can also lead to retention time shifts, in this case, replace the column. Variability in signal intensity may result from errors in MS tuning. Perform a new tuning. Persistent variability may indicate detector deterioration. In this case, request support from the instrument manufacturer.

### Problem 10

High background noise (related to Step 44).

### Potential solution

Aged solvents (older than 15 days) or low-quality solvents can lead to high background noise. Replace all solvents with freshly prepared, high-quality MS-grade solvents. Persistent high background noise may also indicate detector deterioration. In this case, request support from the instrument manufacturer.

### Problem 11

The LOQ of a specific oxylipin is abnormally high in the calibration curve (related to Step 46–52).

### Potential solution

First, verify if the chromatographic peak is correctly integrated. If the problem persists, assess whether the oxylipin has degraded by inspecting the corresponding MS1 and MS2 signals of the calibration curve solutions.

### Problem 12

The LOQ of all oxylipins is abnormally high in the calibration curve (related to Step 46–52).

### Potential solution

Verify the storage conditions of both the calibration curve and the individual oxylipin standards to exclude analyte degradation. Ensure that standards are stored at −80°C. If degradation is unlikely, ion suppression may be occurring due to contamination of the mobile phase or the ion source. In this case, prepare fresh mobile phases, verify solvent cleanliness, and clean the ion source before repeating the analysis.

### Problem 13

The concentration of an oxylipin standard is uncertain (related to Step 46–52).

### Potential solution

Uncertainty regarding the concentration of commercial oxylipin standards may result from solvent evaporation, degradation during shipping, or long-term storage. To assess possible degradation, inject a diluted solution of the compounds and inspect the full-scan mass spectrum for unexpected ions or signals corresponding to degradation products. The standard concentration can be verified by combining UHPLC-MS quantification against a certified standard of a regioisomer or an oxylipin from the same class and PUFA precursor with UV absorbance measurements, when the target oxylipin contains a UV-absorbing moiety.^23^

### Problem 14

Unexpected high levels of hydroxy-PUFA oxylipins (related to Step 54–60).

### Potential solution

Unexpectedly high concentrations of hydroxy-PUFA oxylipins may result from *ex vivo* oxidation during sample collection and handling. Verify that the appropriate concentration of antioxidants is used and that samples are maintained at low temperatures whenever possible throughout the extraction process. In addition, minimize sample handling time and avoid unnecessary exposure to air and light.

## Resource availability

### Lead contact

Further information and requests for resources and reagents should be directed to and will be fulfilled by the lead contact, Sayuri Miyamoto (miyamoto@iq.usp.br).

### Technical contact

Technical questions on executing this protocol should be directed to and will be answered by the technical contact, Sayuri Miyamoto (miyamoto@iq.usp.br).

### Materials availability

This study did not generate new unique reagents.

### Data and code availability

The *staRoxy* (v0.2.0) package used in this protocol is archived on Zenodo (https://doi.org/10.5281/zenodo.20126956). The latest development version, including a comprehensive workflow vignette and installation instructions, is available on its GitHub repository (https://github.com/pefjordao/staRoxy).

## Acknowledgments

S.M. acknowledges support from the 10.13039/501100001807São Paulo Research Foundation (FAPESP) (2024/16848-3, 2026/01376-4, and CEPID-Redoxoma 2013/07937-8), the 10.13039/501100003593National Council for Scientific and Technological Development (CNPq) (309083/2017-6, 313926/2021-2, 313162/2026-3, and INCT 408213/2024-8), and from NAP-Redoxoma and the Pró-Reitoria de Pesquisa e Inovação da Universidade de São Paulo (PRPI-USP). L.R.D. acknowledges a scholarship from the 10.13039/100031771Fundação de Apoio à Universidade de São Paulo (FUSP) and Coordenação de Aperfeiçoamento de Pessoal Superior (10.13039/501100002322CAPES) (#88882.332987/2019-01). R.S.S. (nos. 2021/13711-9 and 2023/08462-5), P.H.F.J. (no. 2025/00242-1), and A.C.A.Z. (no. 2024/17462-1) acknowledge scholarships from 10.13039/501100001807FAPESP. B.B. acknowledges support from the 10.13039/501100001659Deutsche Forschungsgemeinschaft (10.13039/501100001659DFG, German Research Foundation) under project nos. 511488495 SFB 1638/1 (Z02) and 538651361 FOR 5815 (P3/Z) and from the Federal Ministry of Education and Research (BMBF) under grant no. FKZ 01EJ2503B. C.E.W. acknowledges support from the 10.13039/501100003793Swedish Heart-Lung Foundation (HLF 20230463, HLF 20210519) and the 10.13039/501100001862Swedish Research Council (2022-00796). A.S., A.B.C.F., and M.T.S. acknowledge funding from the 10.13039/501100001659German Research Foundation as part of SFB 1550 (project A01, 464424253), FOR 5815 (project P02, 538651361), and SPP 2306 (project 461705295). The authors also acknowledge support from 10.13039/501100002322CAPES, Finance Code 001. The graphical abstract was created in BioRender. Miyamoto, S. (2026) https://BioRender.com/vawbcrd. The *staRoxy* logo was created and kindly provided by Cynthia Daiane Diniz.

## Author contributions

Methodology, A.B.C.F., L.R.D., R.S.S., M.T.S., M.T.B., and J.K.; package development, P.H.F.J.; formal analysis, A.B.C.F., L.R.D., R.S.S., A.C.A.Z., and P.H.F.J.; writing – original draft, A.B.C.F., L.R.D., R.S.S., M.T.S., P.H.F.J., and A.C.A.Z.; review and editing, all authors; supervision, S.M., A.S., B.B., C.E.W., and E.M.R.

## Declaration of interests

The authors declare no competing interests.

## Supplemental information

Document S1. Figure S1 and Data S1

Table S1. Harmonized nomenclature for oxylipins and related lipid mediators included in the analytical panel, related to Step 62

Table S2. Preparation of oxylipin standard stock solutions, related to Step 5

Table S3. Preparation of the oxylipin mix solution and calibration curve standards (A - N), related to Step 8

Table S4. LC-MS/MS characterization of oxylipin species using the SCIEX TripleTOF® 6600, related to Step 32

Table S5. LC-MS/MS characterization of the deuterated internal standards using the SCIEX TripleTOF® 6600, related to Step 32

Table S6. LC-MS/MS characterization of oxylipin species using the Thermo Q-Exactive Plus, related to Step 36

Table S7. LC-MS/MS characterization of oxylipin species using the Thermo Orbitrap Exploris 240, related to Step 41

Table S8. Oxylipin abundance data from LPS-stimulated RAW 264.7 macrophage cell pellets, related to Expected Outcomes

Table S9. Oxylipin abundance data from LPS-stimulated RAW 264.7 macrophage supernatants, related to Expected Outcomes

Table S10. Comparison of oxylipin detection in LPS- or KLA-stimulated RAW 264.7 macrophages across the literature, related to Expected Outcomes
