# Supplementary material for: Protocol for targeted analysis of oxylipins in rat plasma and mouse macrophage samples by high-resolution UHPLC-MS/MS
Source: STAR Protoc. 2026 Sep 18;7(4):104834. doi: 10.1016/j.xpro.2026.104834 (PMC13602319; doi:10.1016/j.xpro.2026.104834)
Supplement: Supplementary file 1 [file mmc1.pdf]

Data S1. Representative peak integration profiles, related to Step 50.

---

tetranor-12(S)-HETE

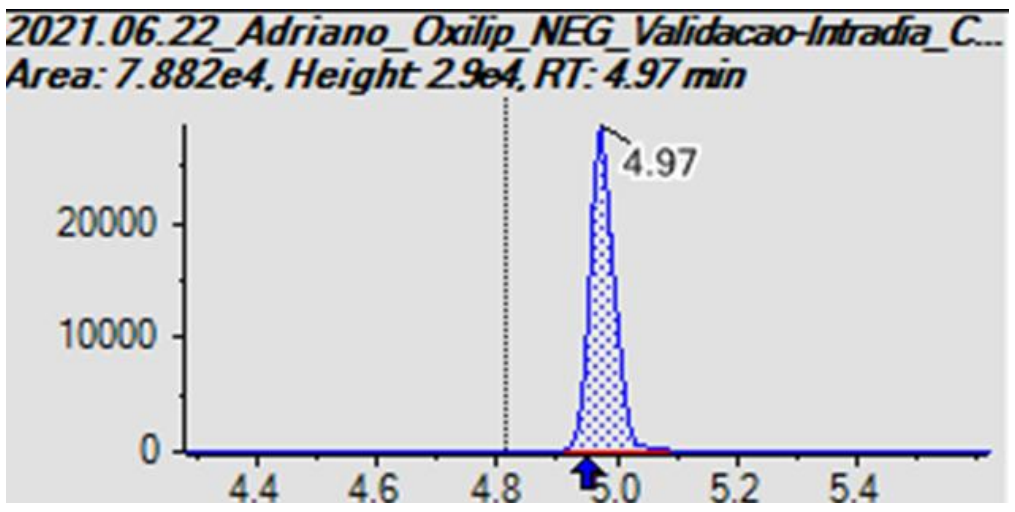

12(S)HHTrE

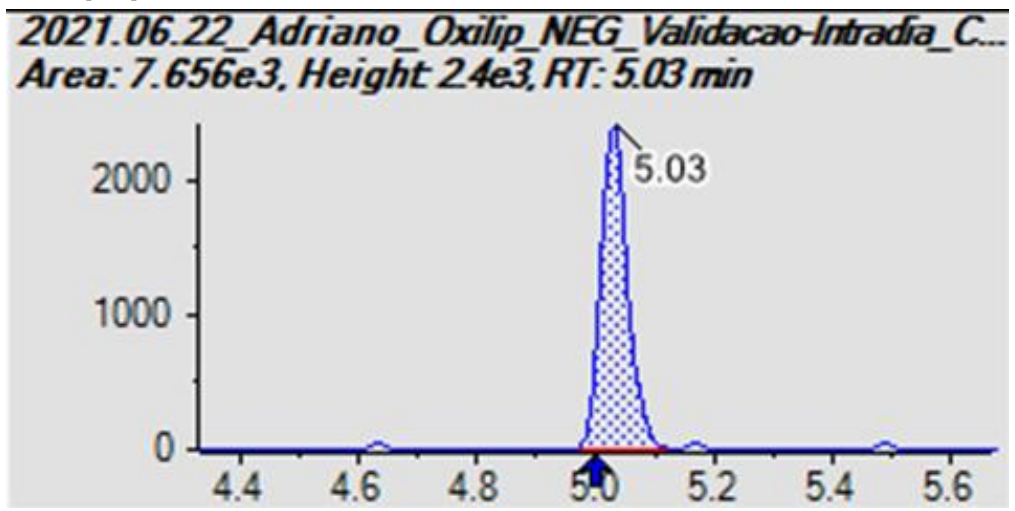

13-OxoODE

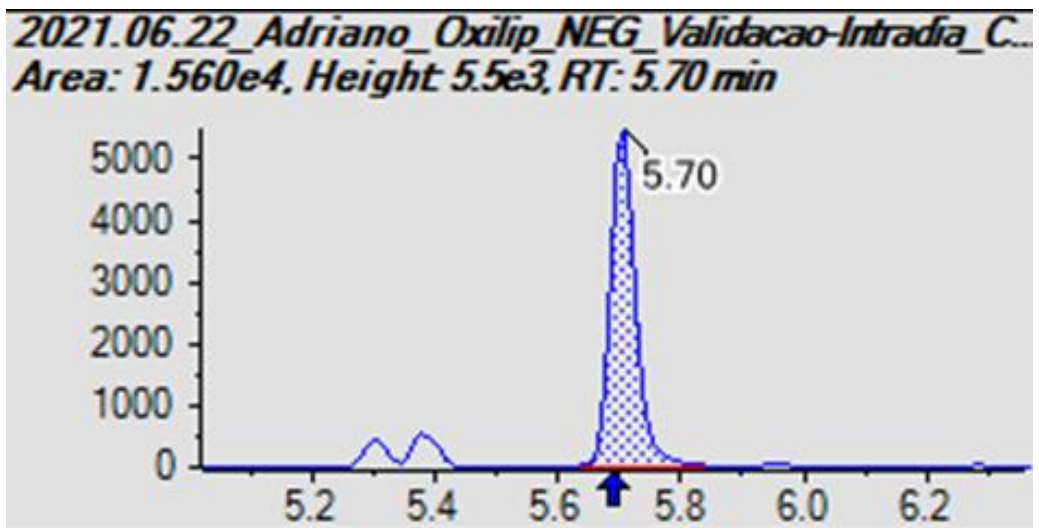

9(S)-HOTrE

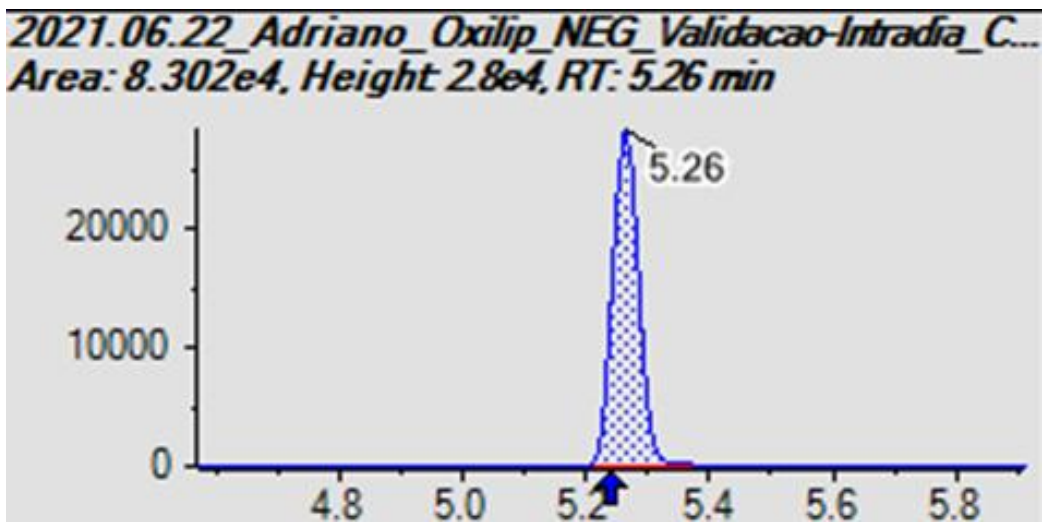

9-oxo-ODE

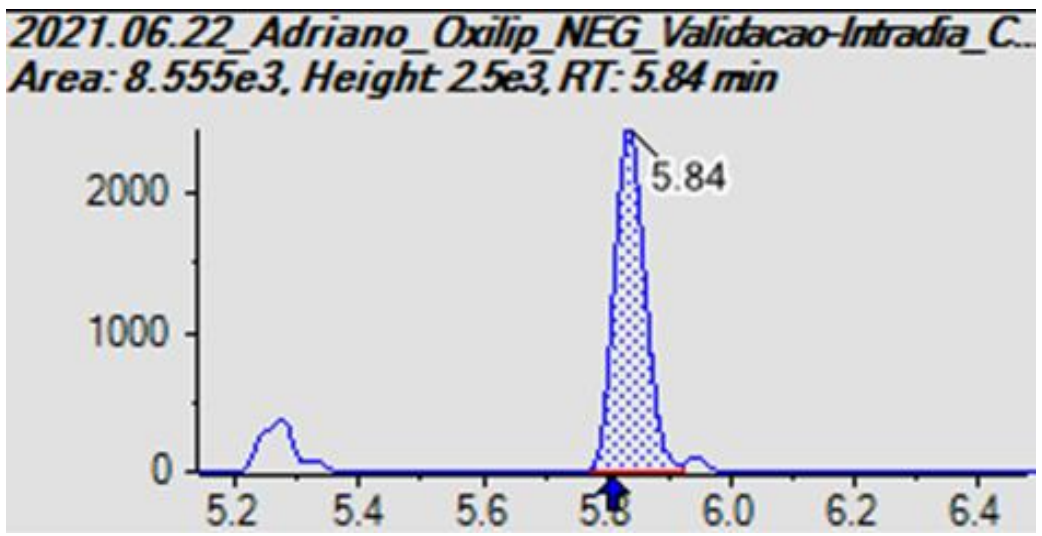

13(S)-HOTrE(γ)

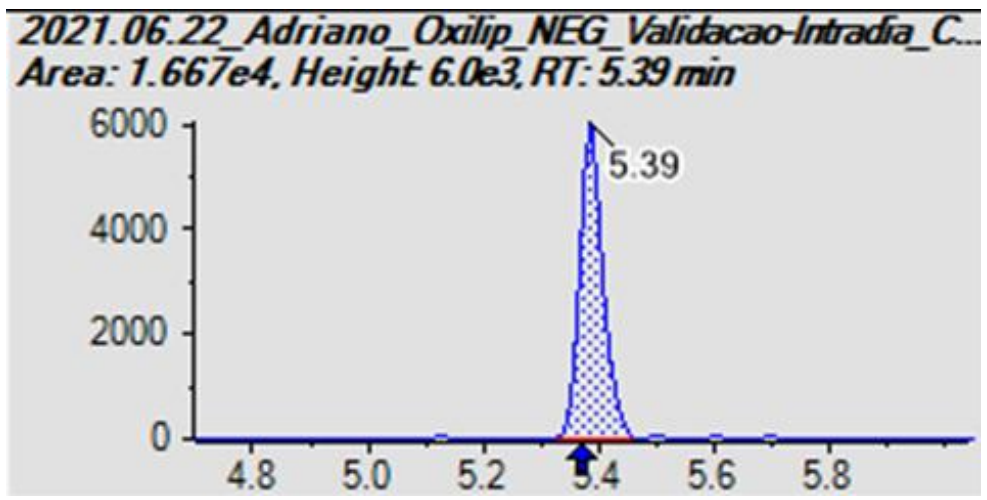

13(S)-HOTrE

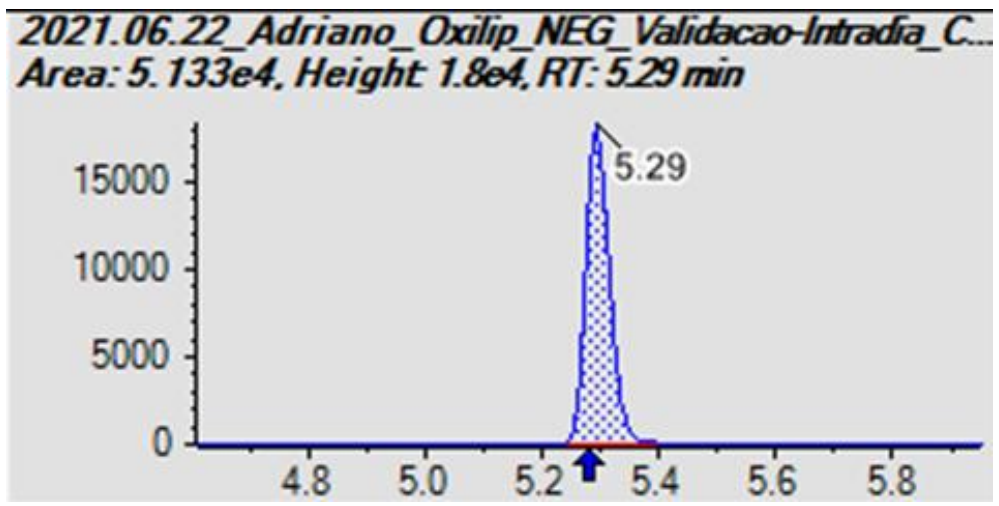

9(S)-HODE

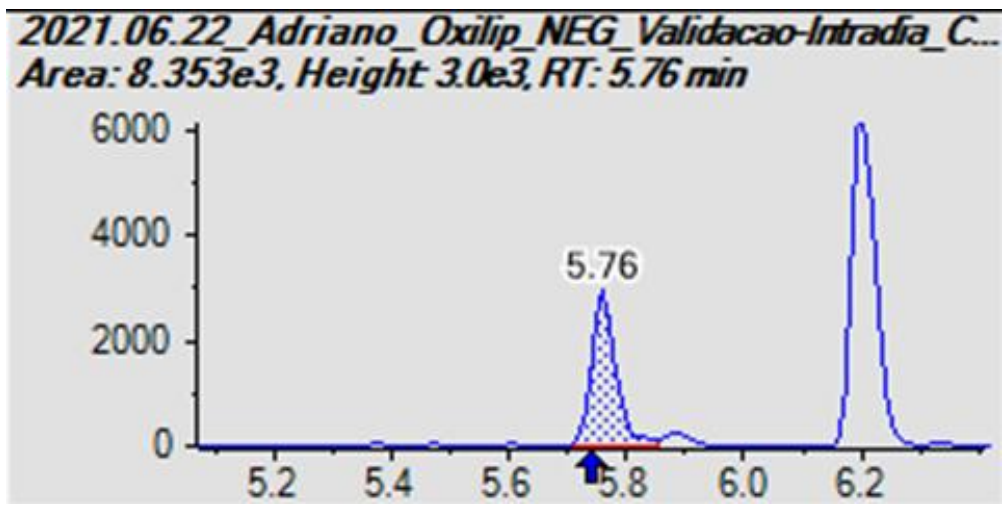

(±)9(10)-EpOME

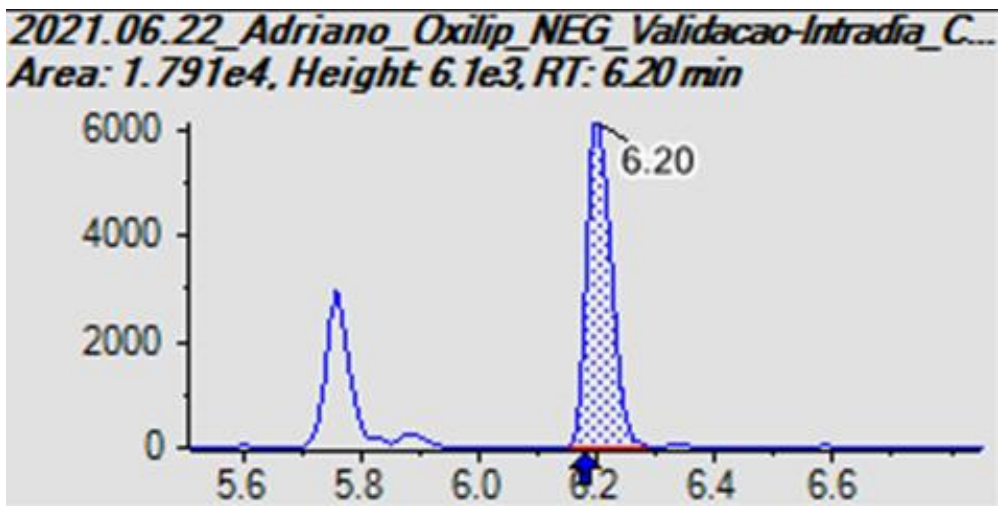

10-HODE

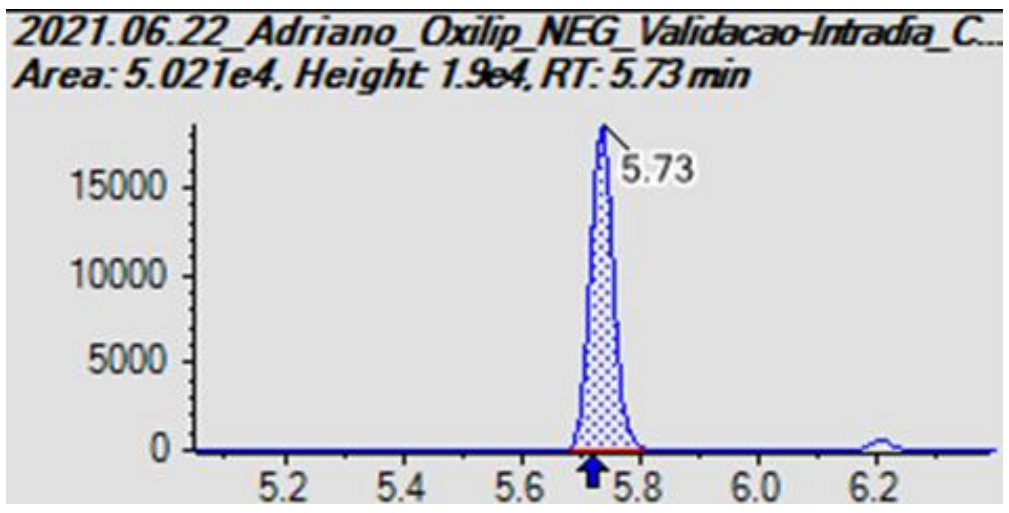

12-HODE

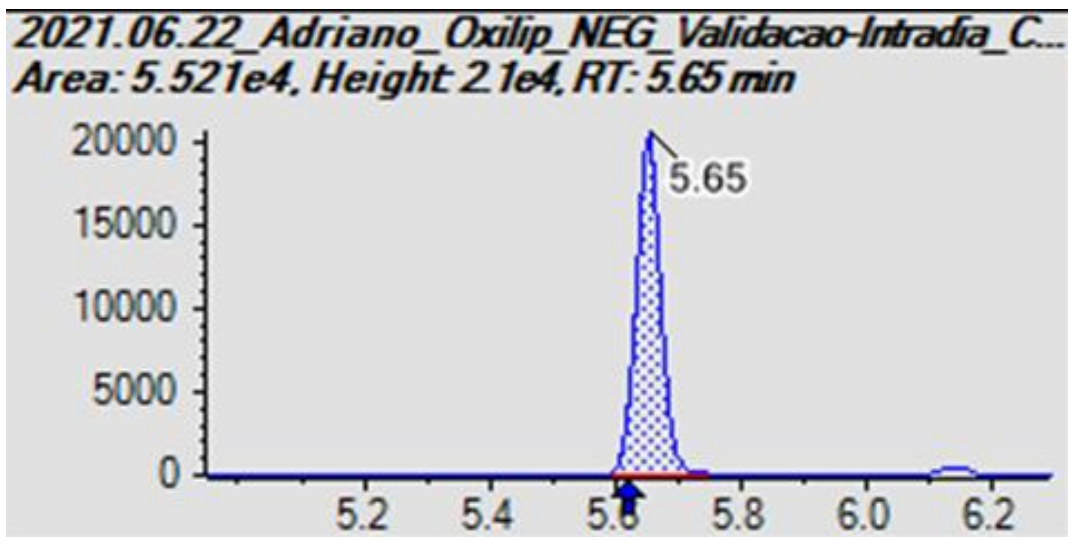

13(S)-HODE

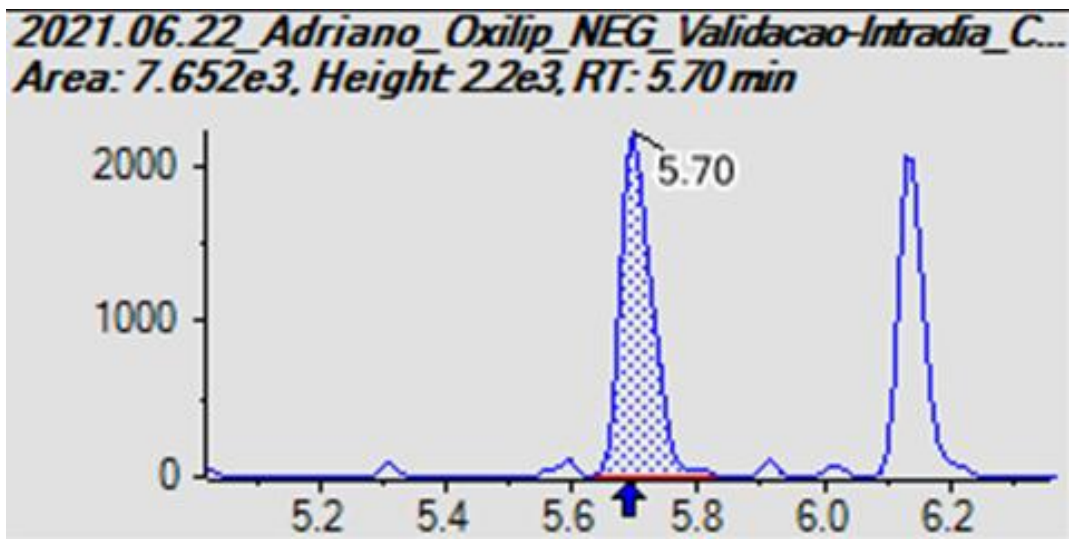

(±)12(13)-EpOME

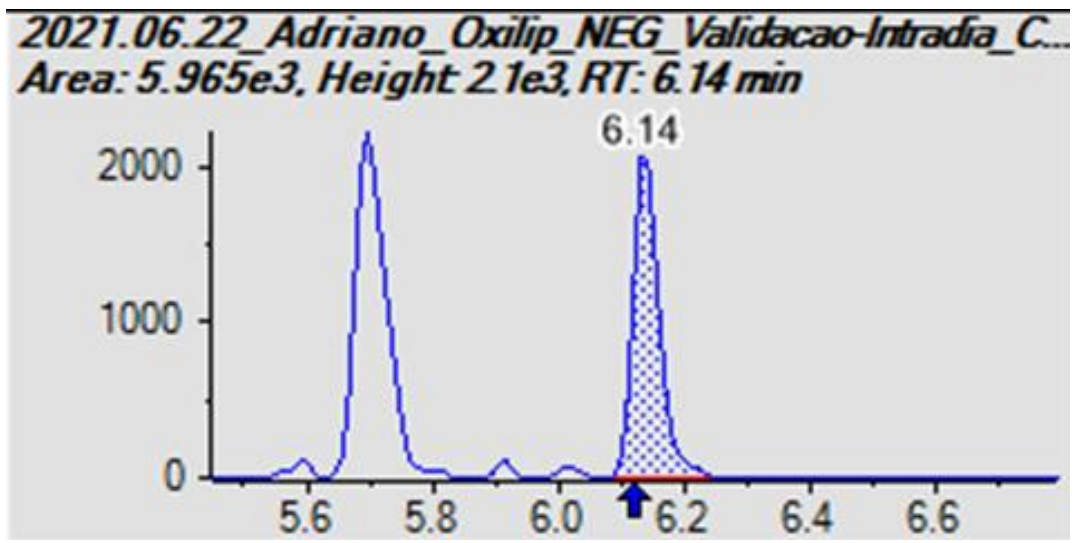

10-HOA

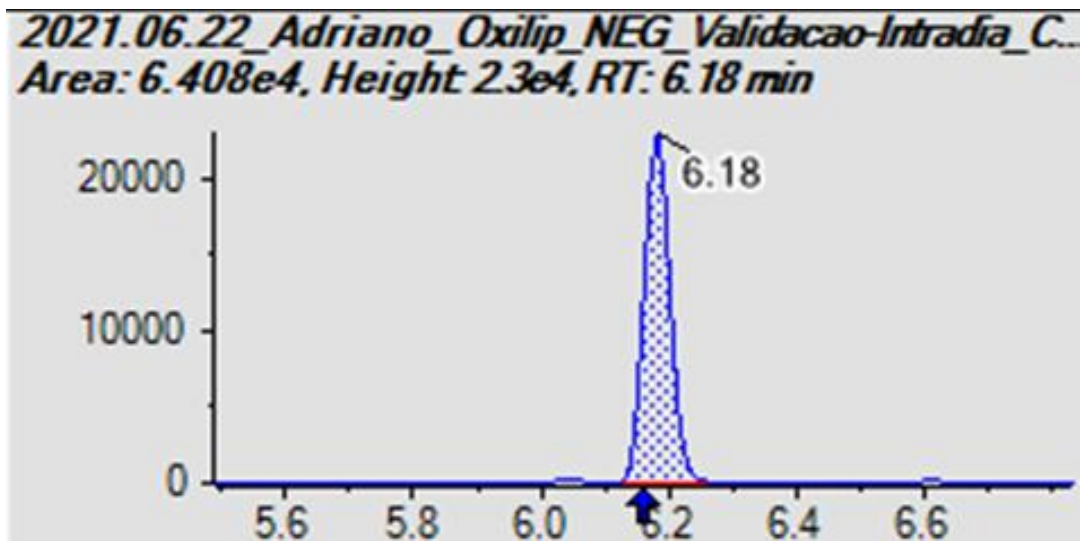

9-HOA

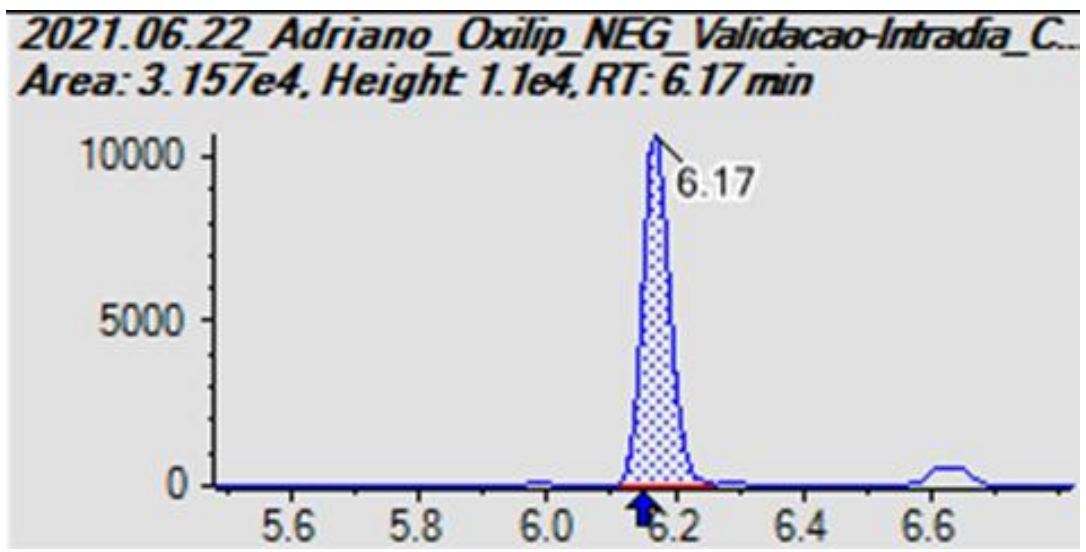

(±)12(13)-DiHOME

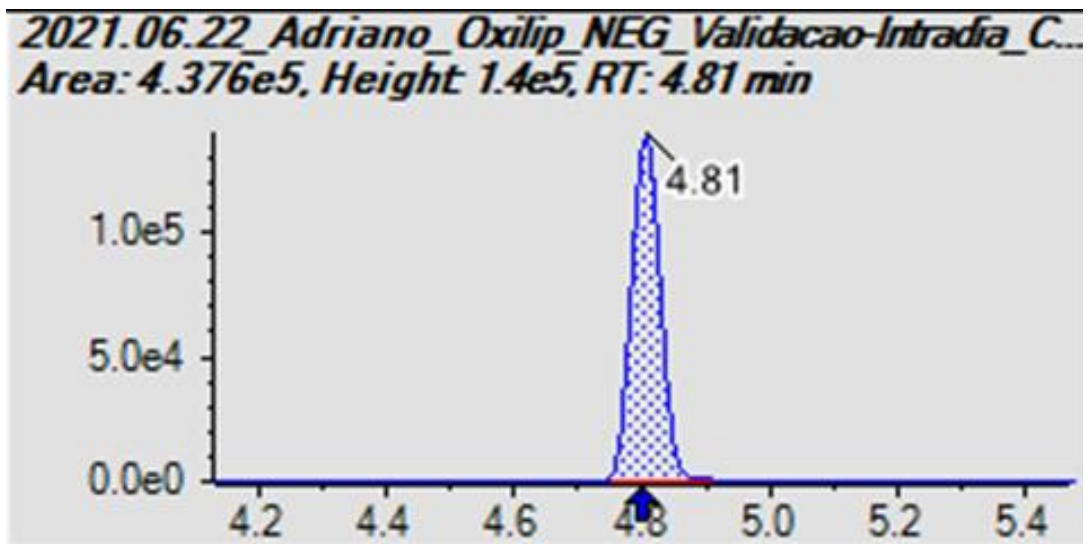

(±)12(13)-DiHOME

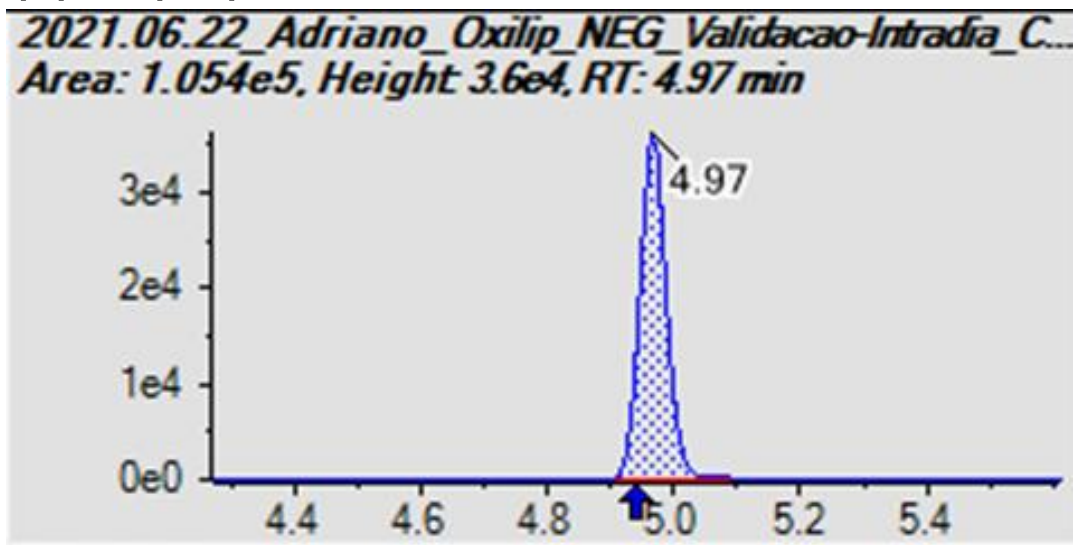

15-deoxy-Δ12,14-Prostaglandin J2

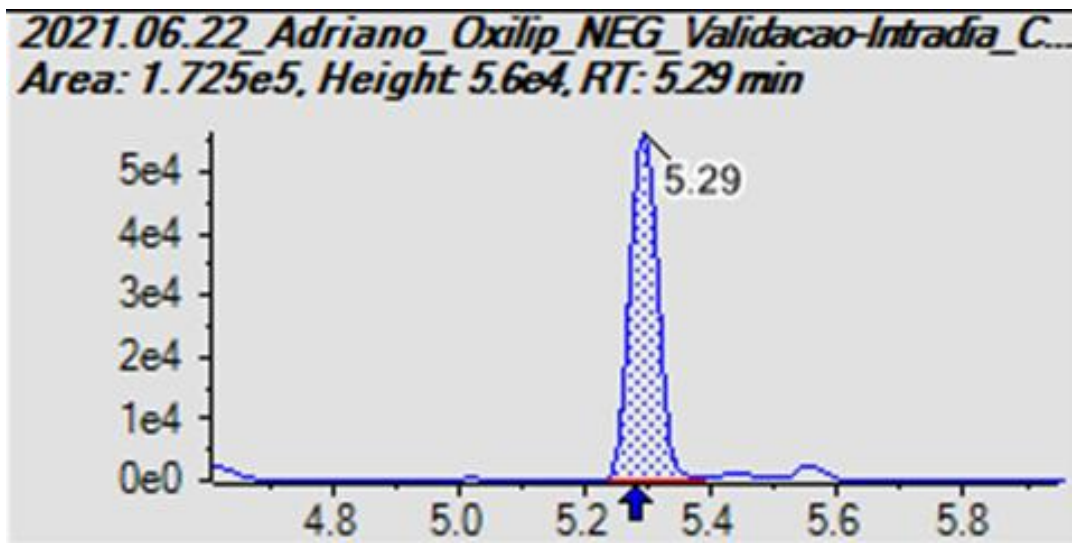

15-OxoETE

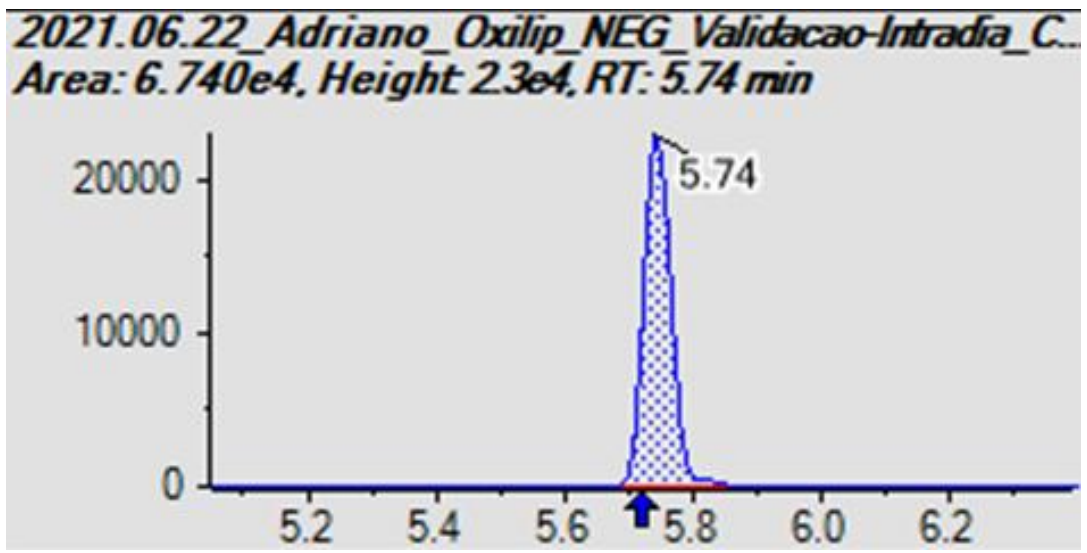

(±)5-HEPE

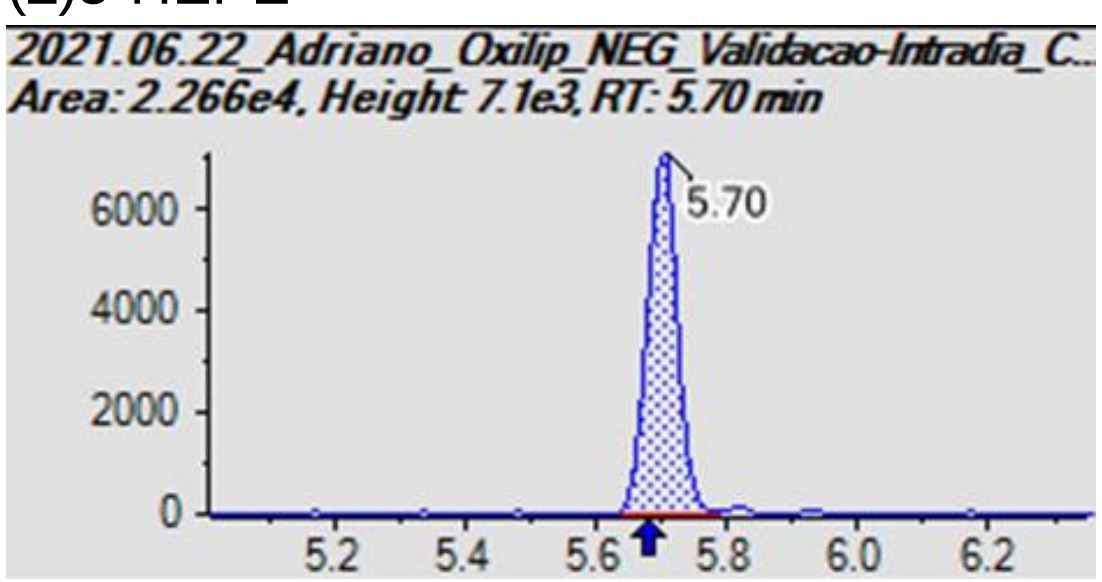

(±)9-HEPE

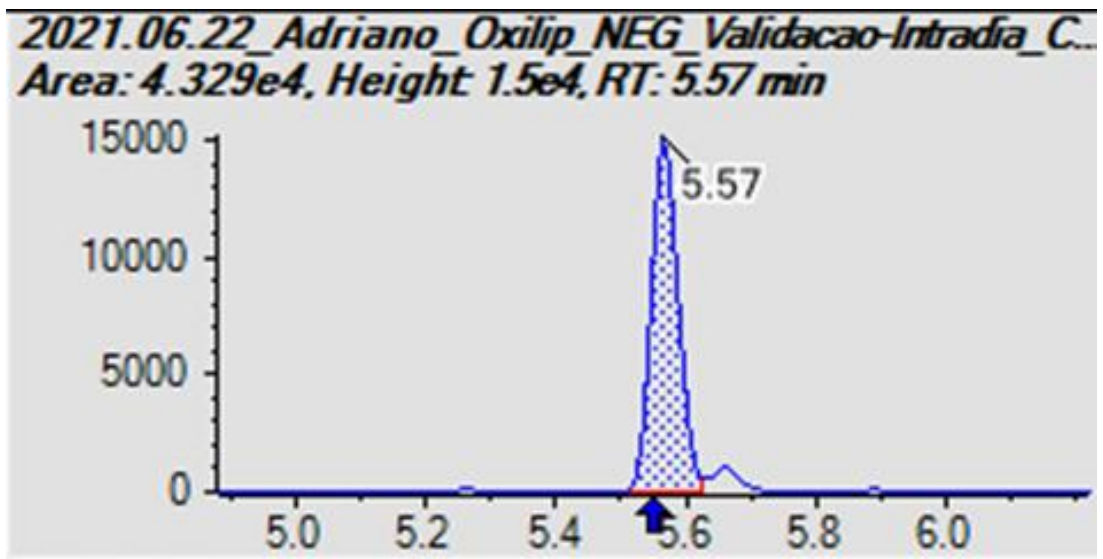

(±)8-HEPE

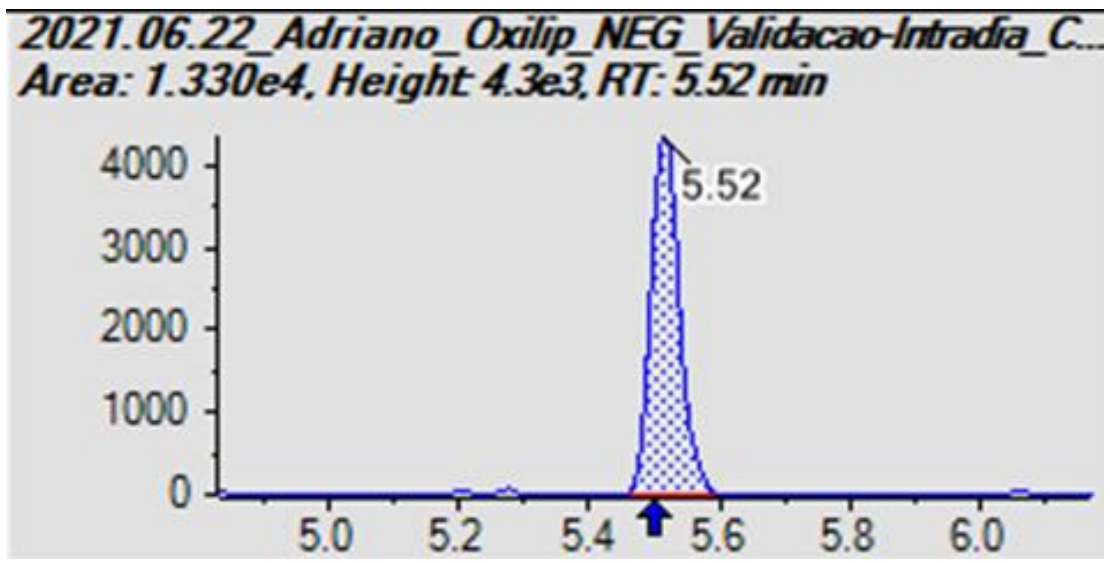

(±)11-HEPE

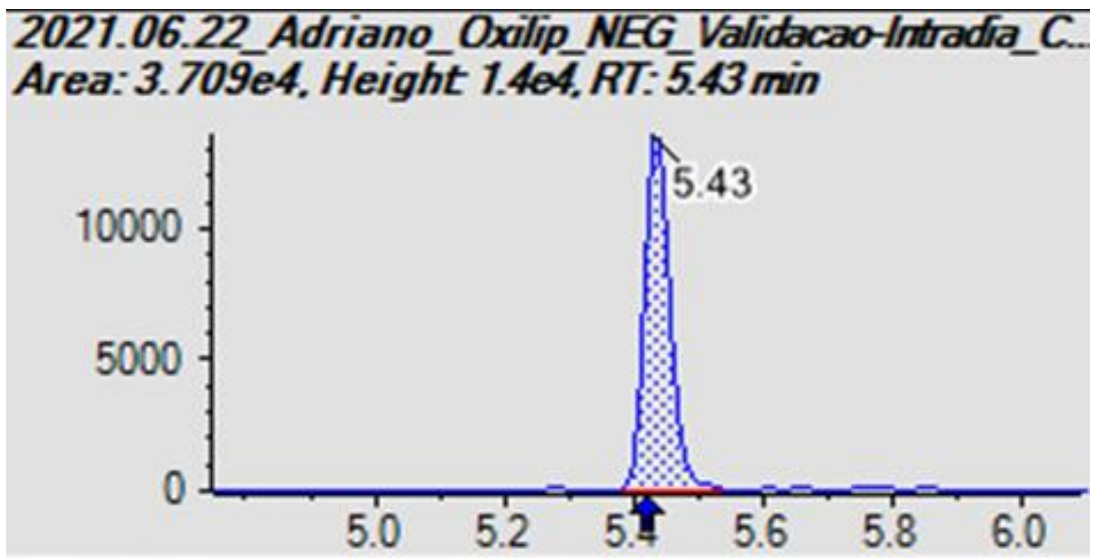

(±)12-HEPE

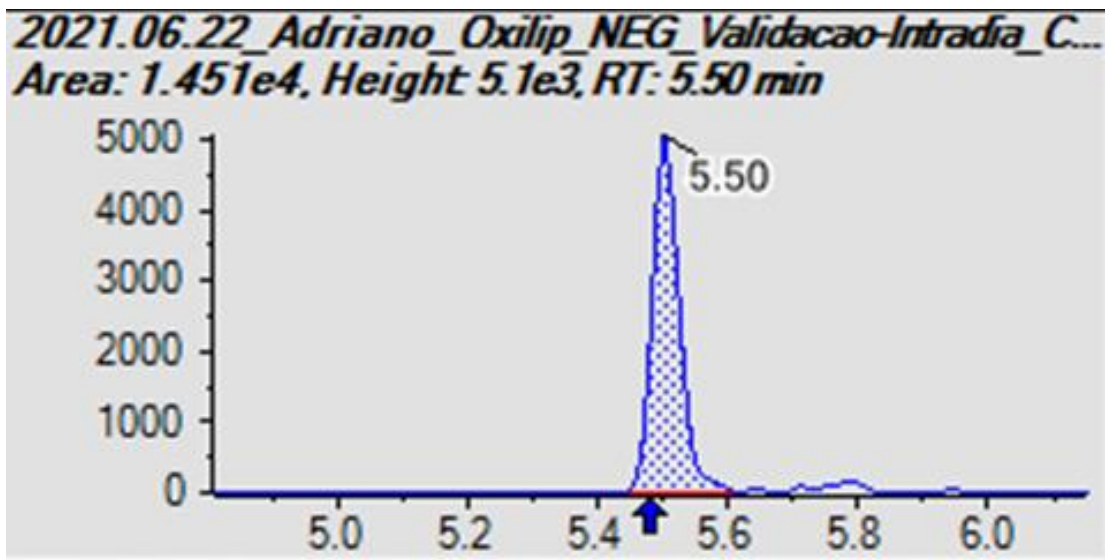

(±)14(15)-EpETE

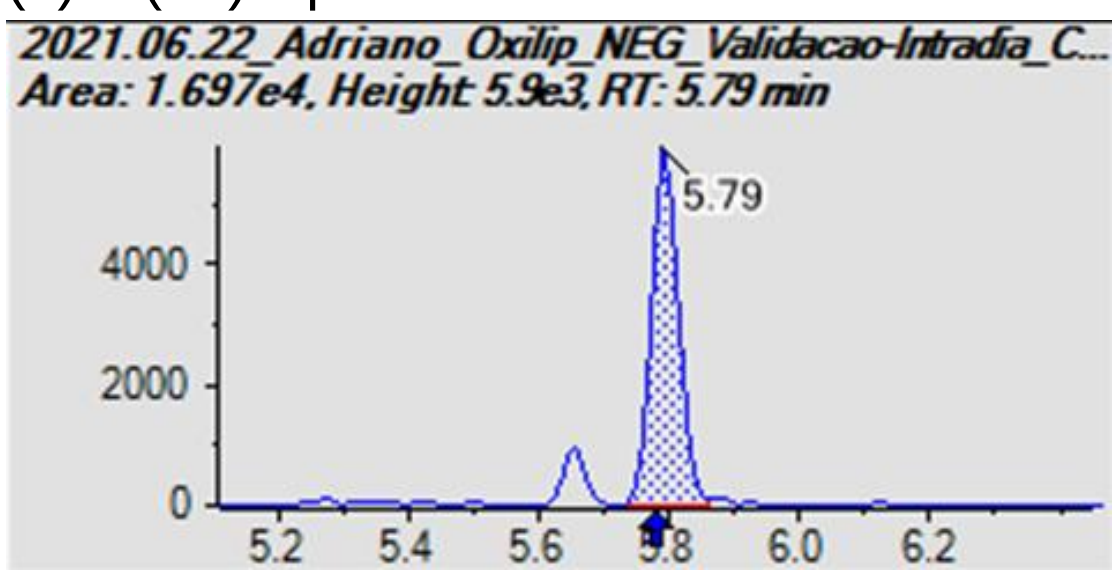

(±)18-HEPE

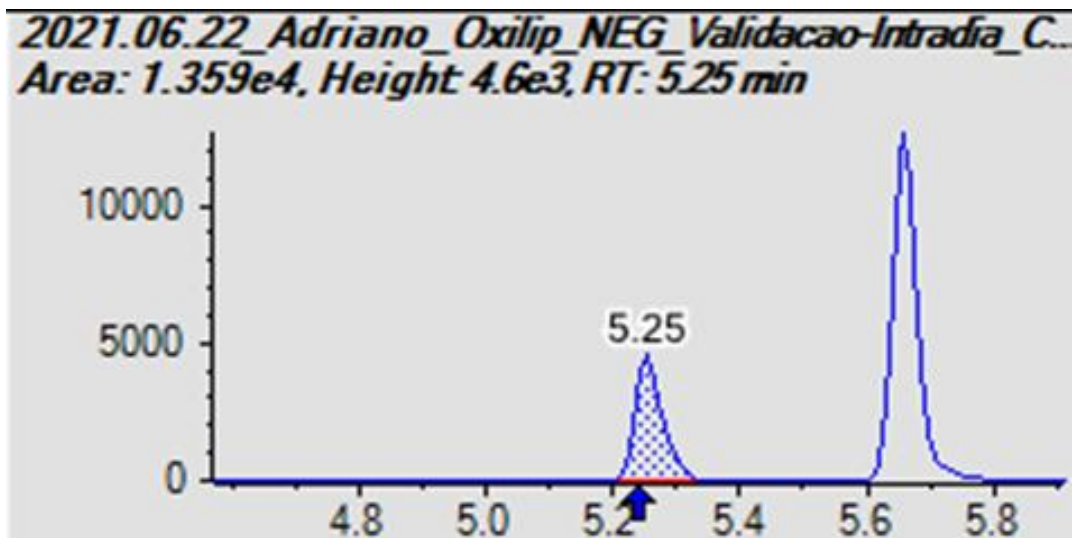

(±)17(18)-EpETE

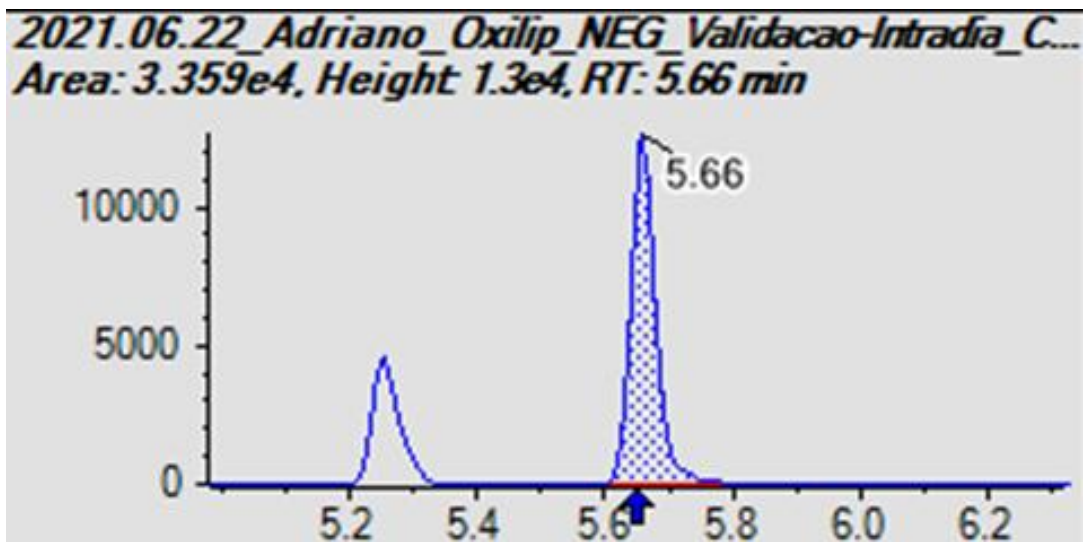

(±)15-HEPE

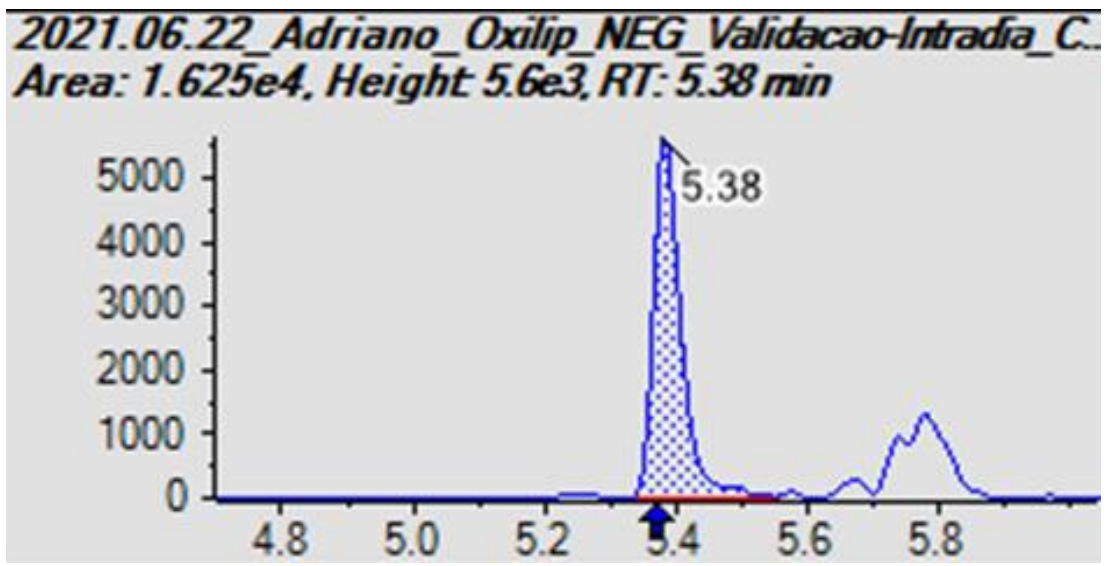

(±)5-HETE

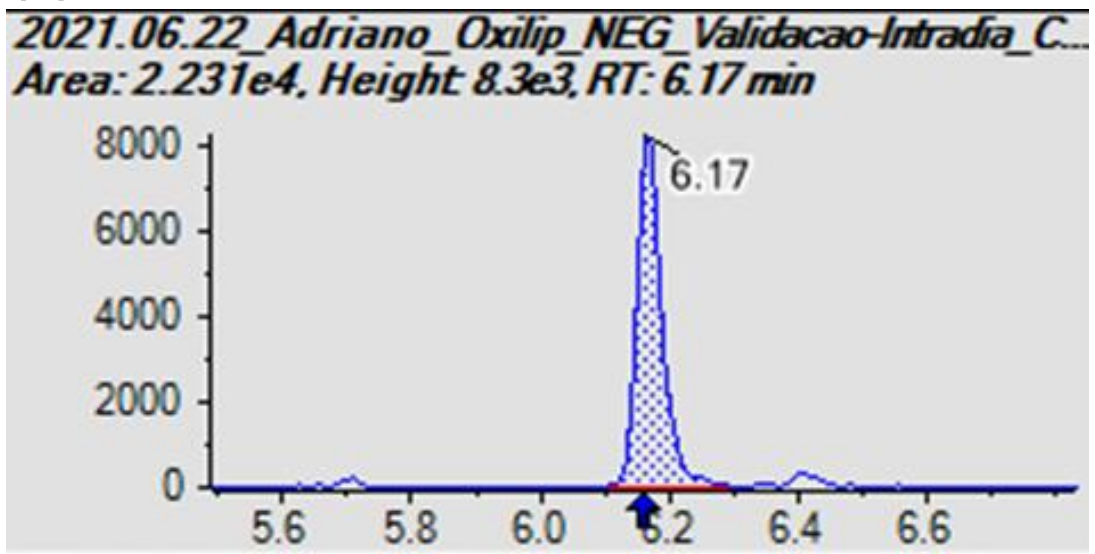

(±)8(9)-EET

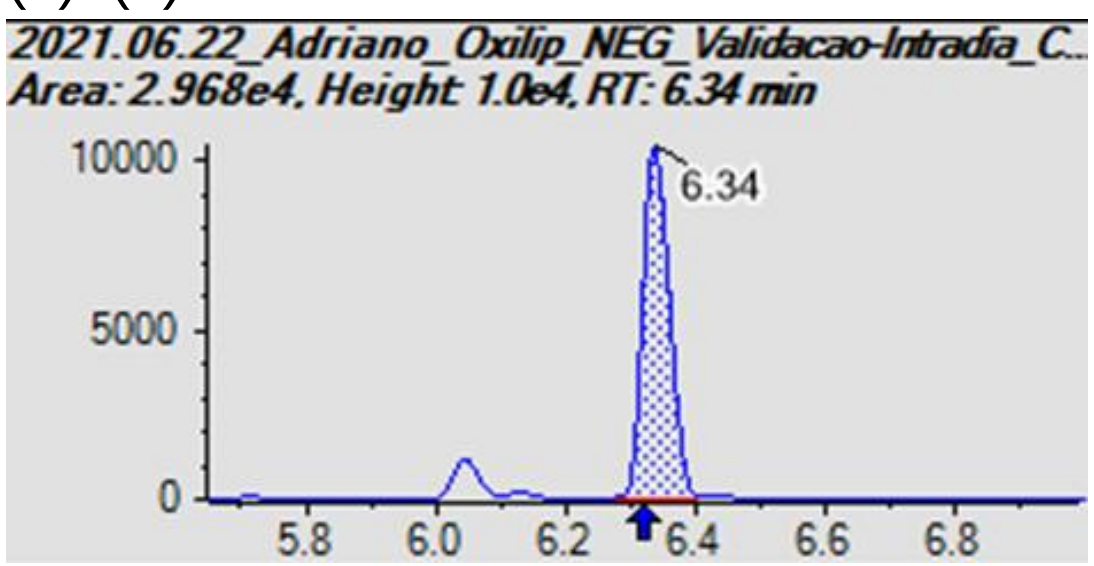

(±)9-HETE

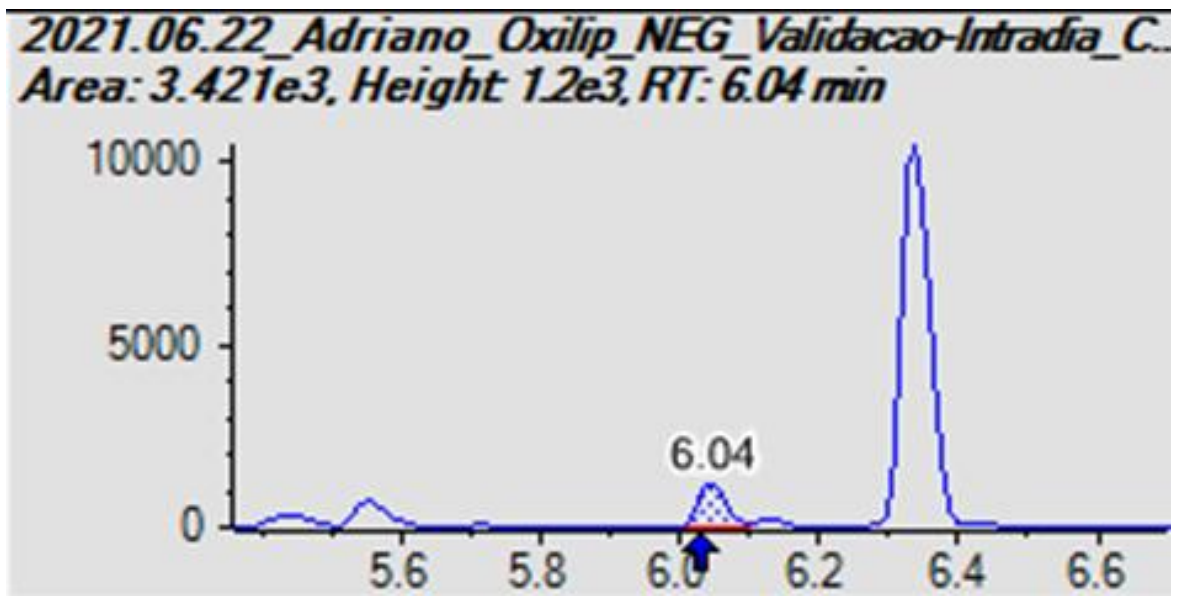

(±)8-HETE

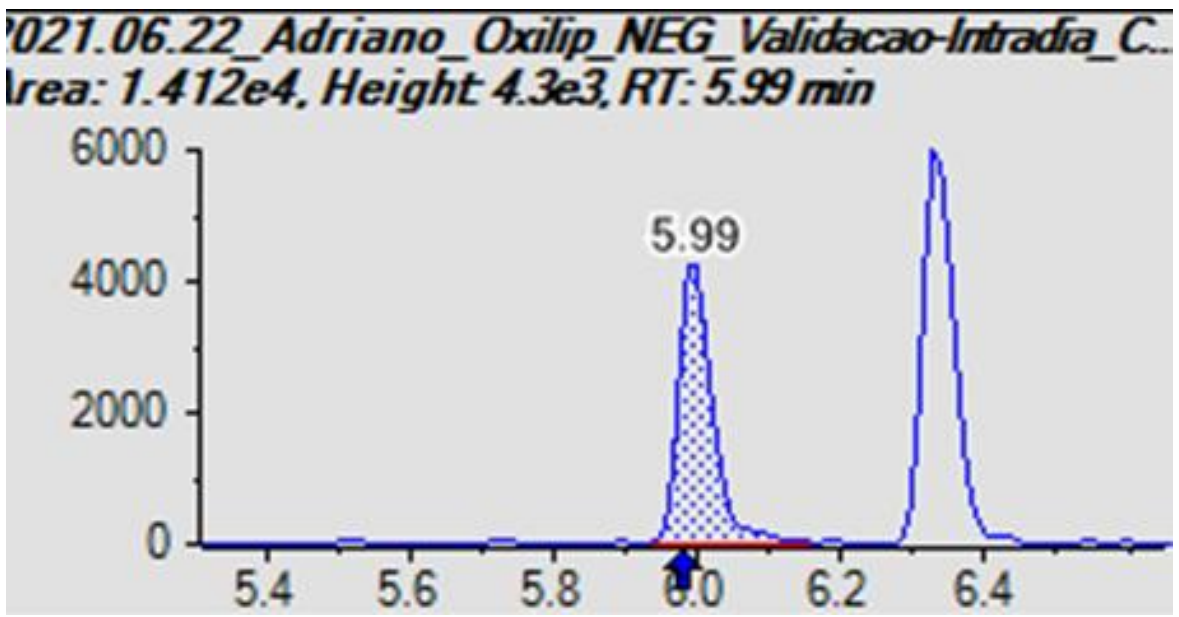

(±)11(12)-EET

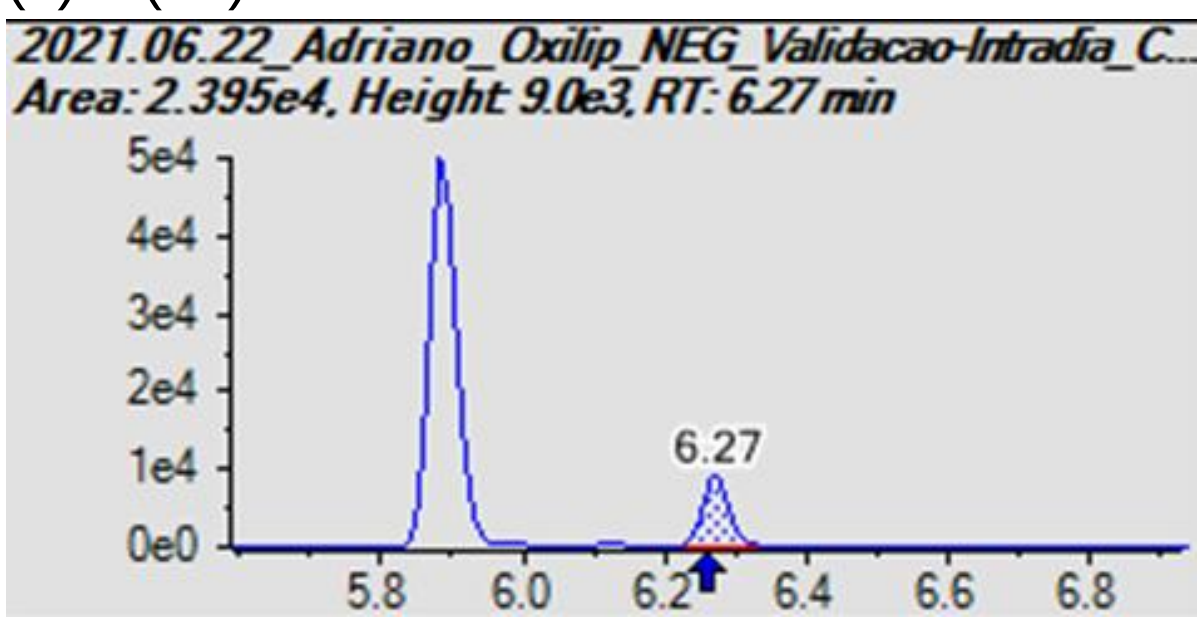

(±)11-HETE

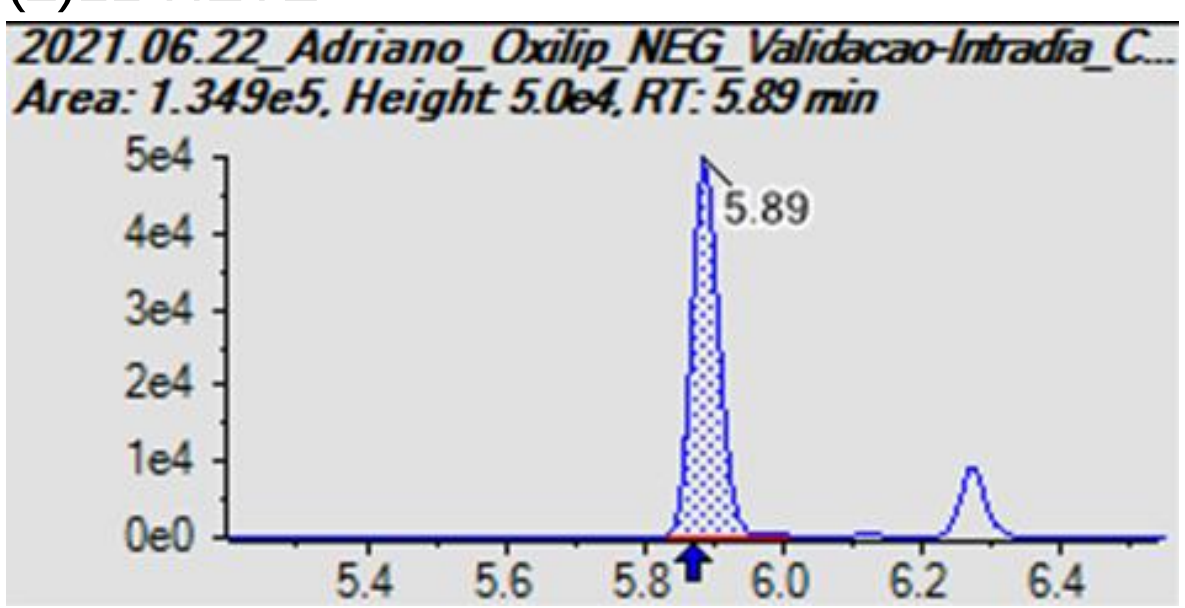

(±)12-HETE

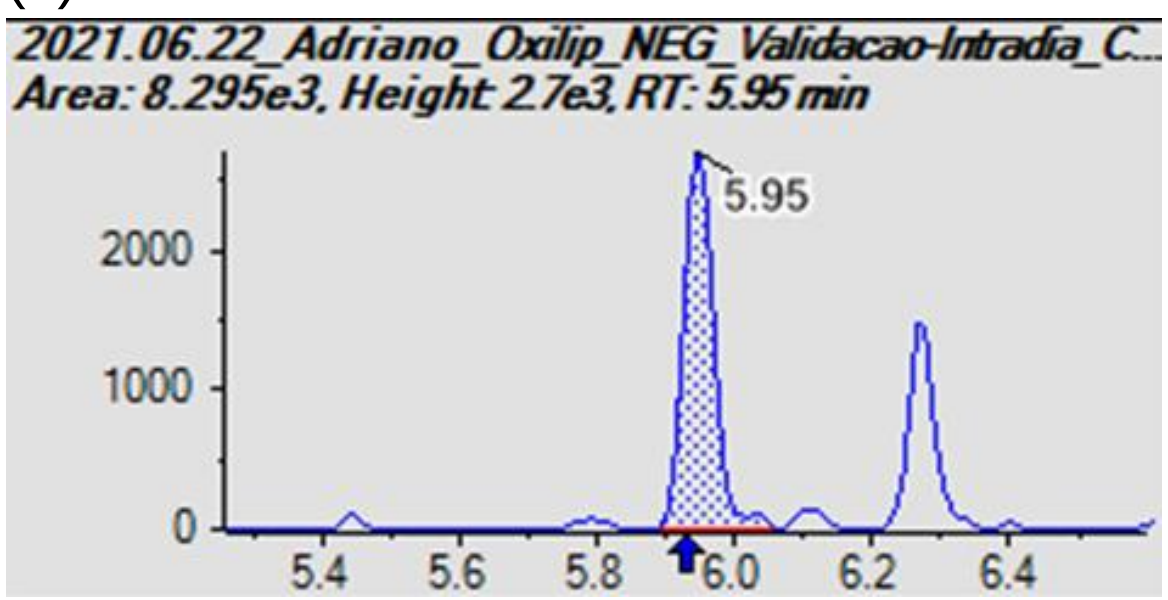

(±)5(6)-EET

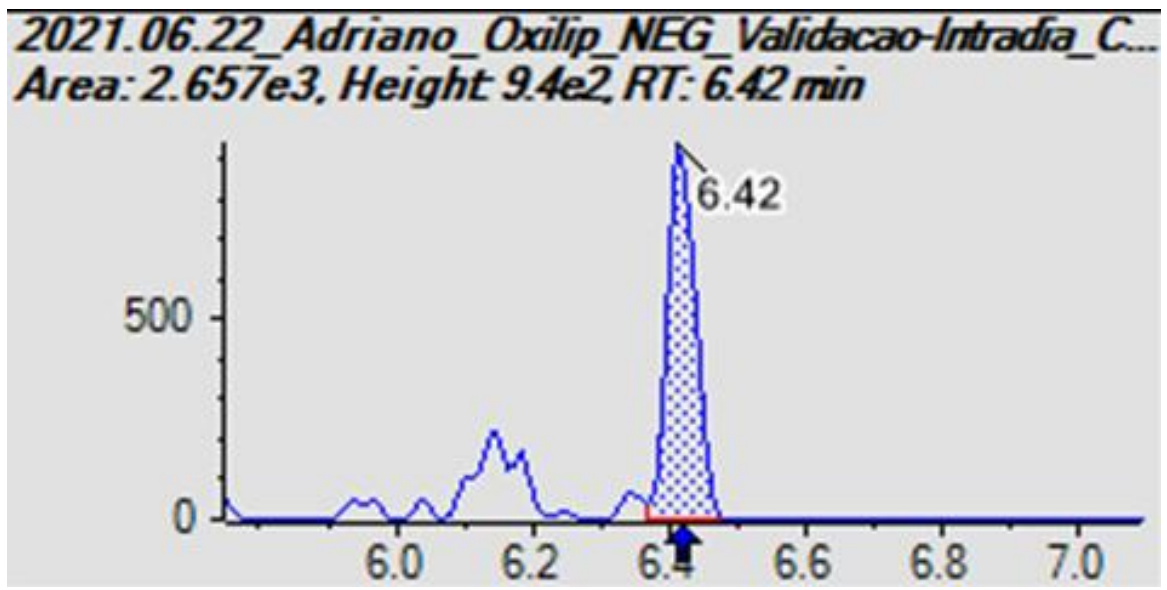

(±)14(15)-EET

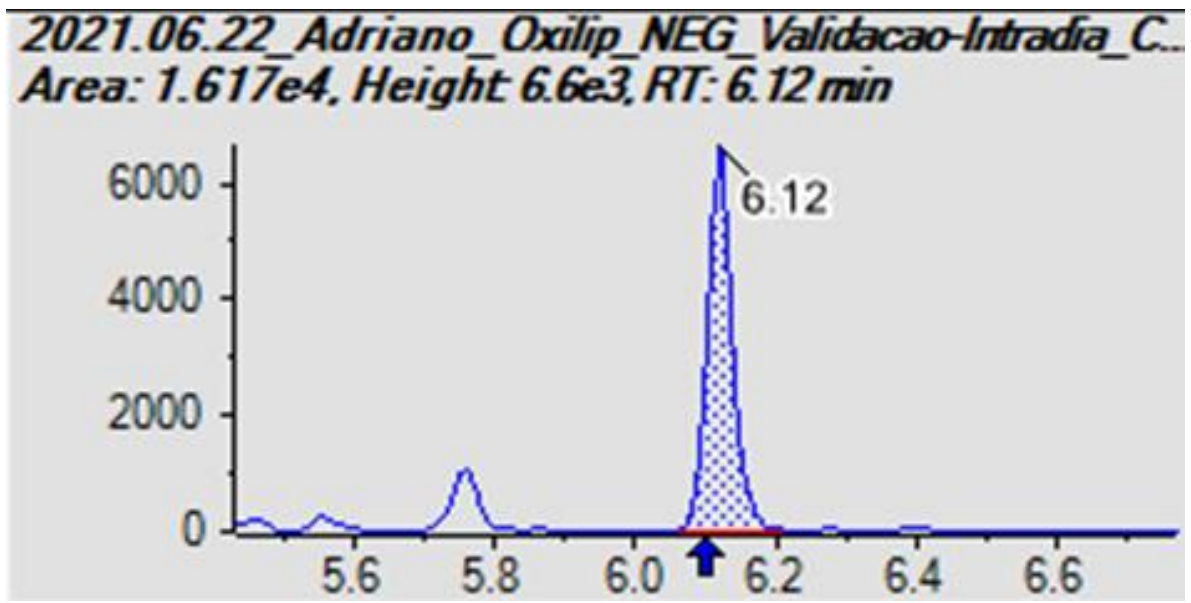

(±)16-HETE

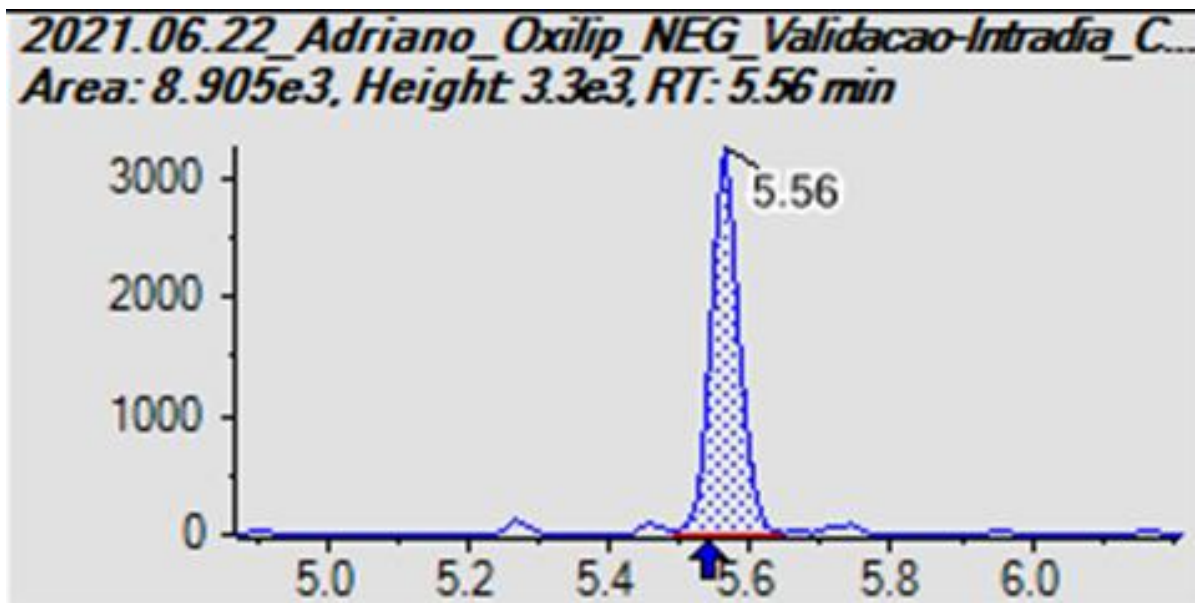

(±)17-HETE

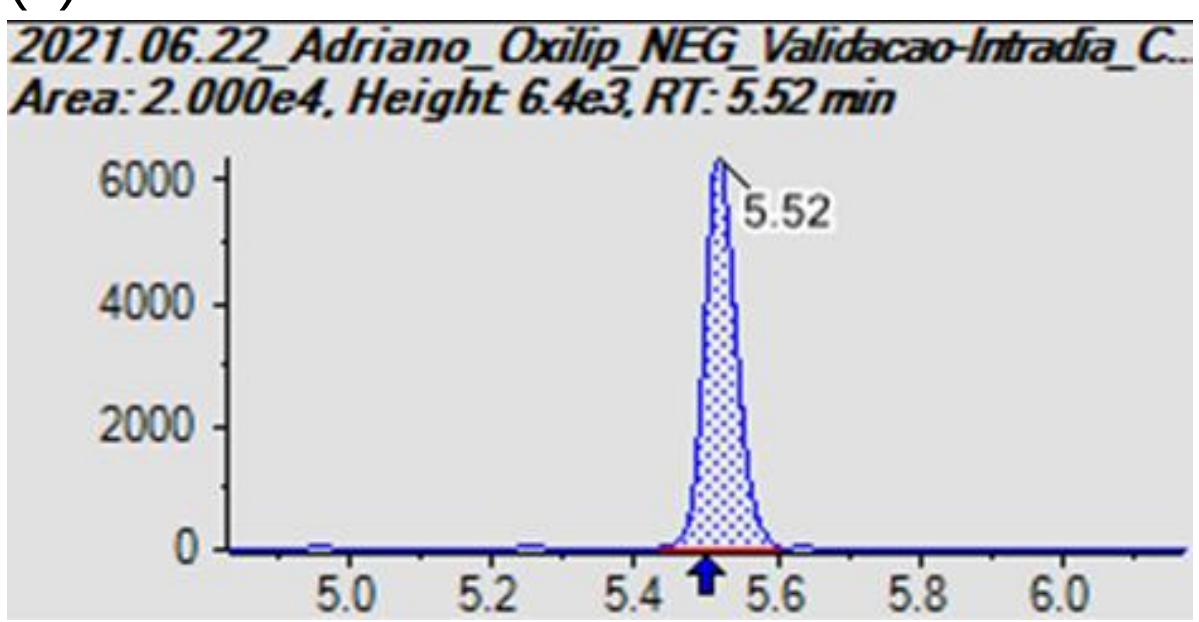

(±)18-HETE

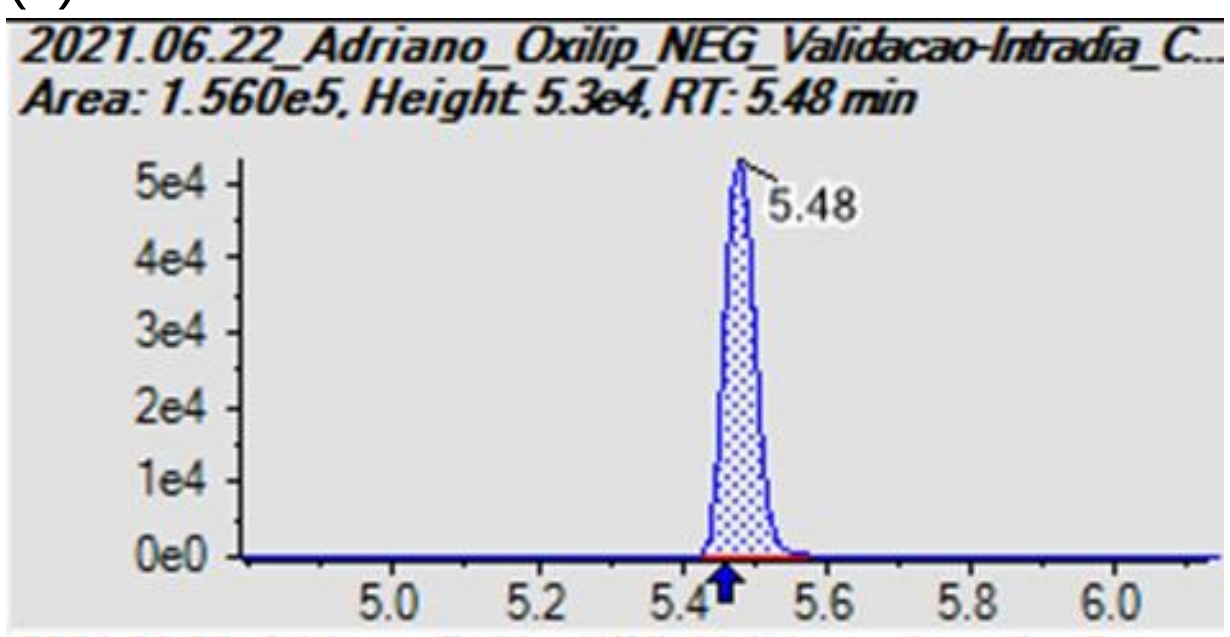

(±)19-HETE

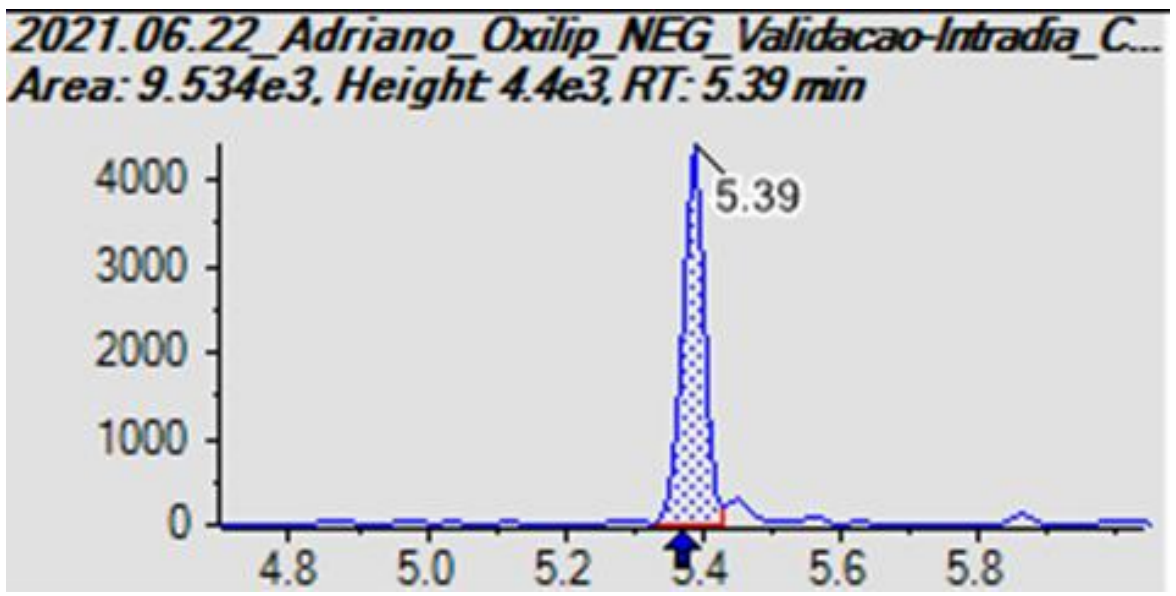

(±)20-HETE

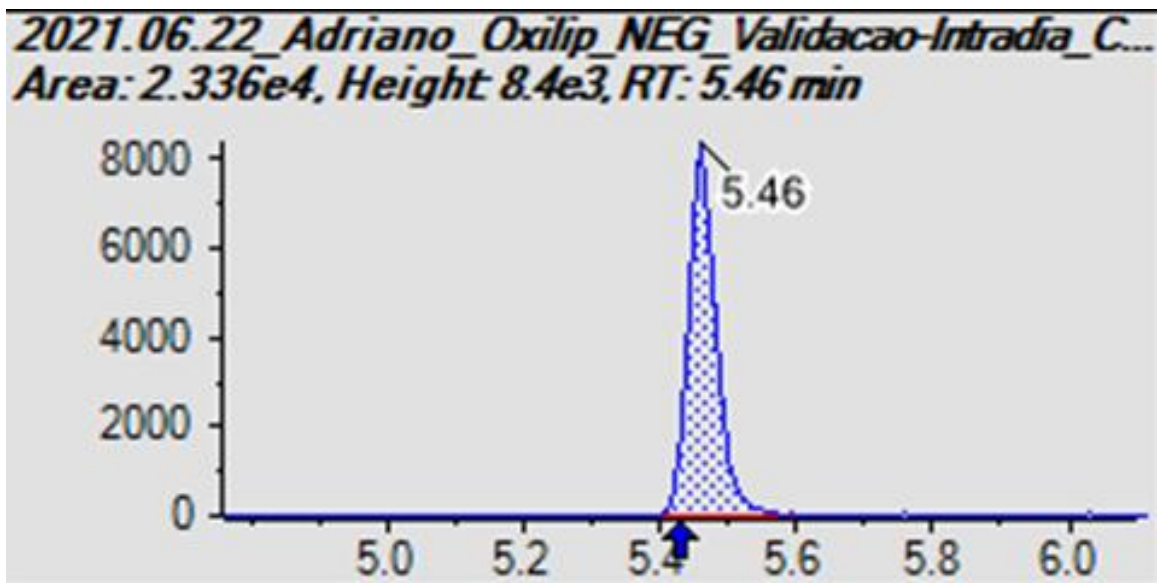

14 and 15-HETE

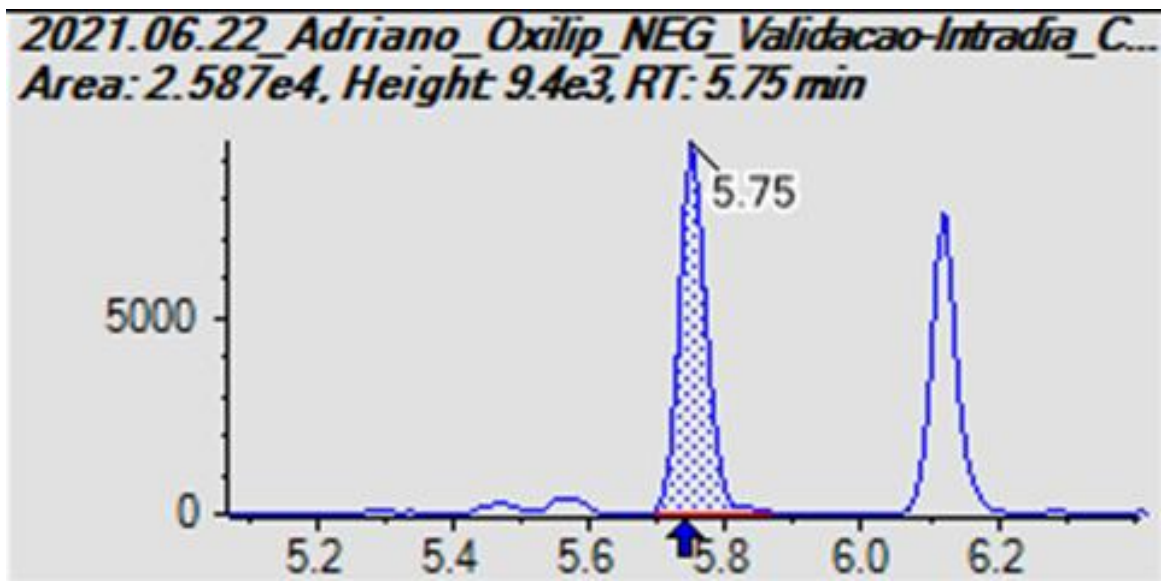

15-OxoEDE

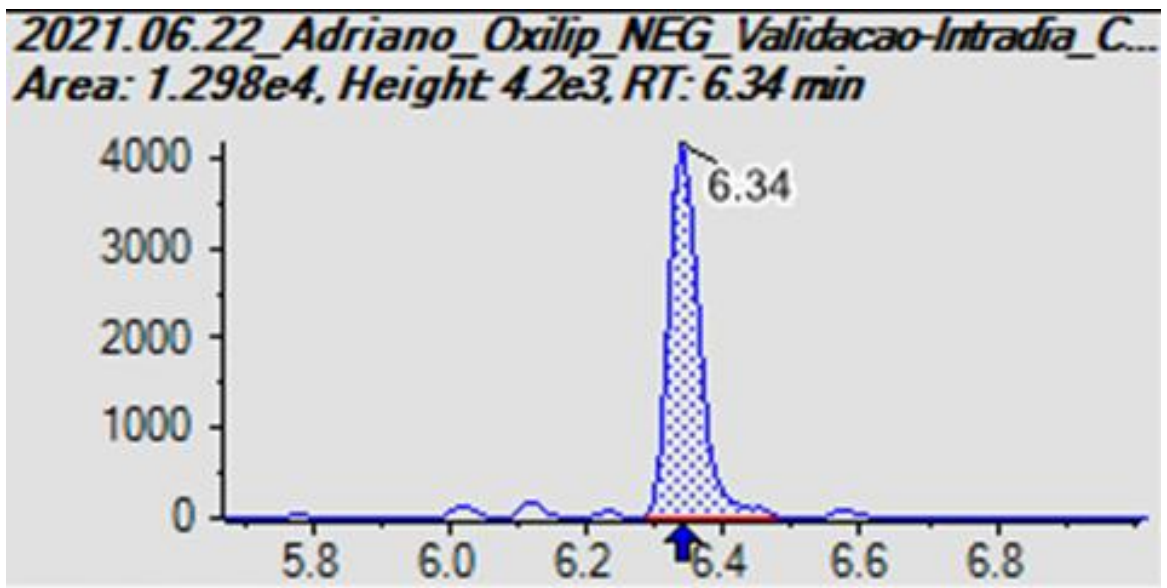

5(S)-HETrE

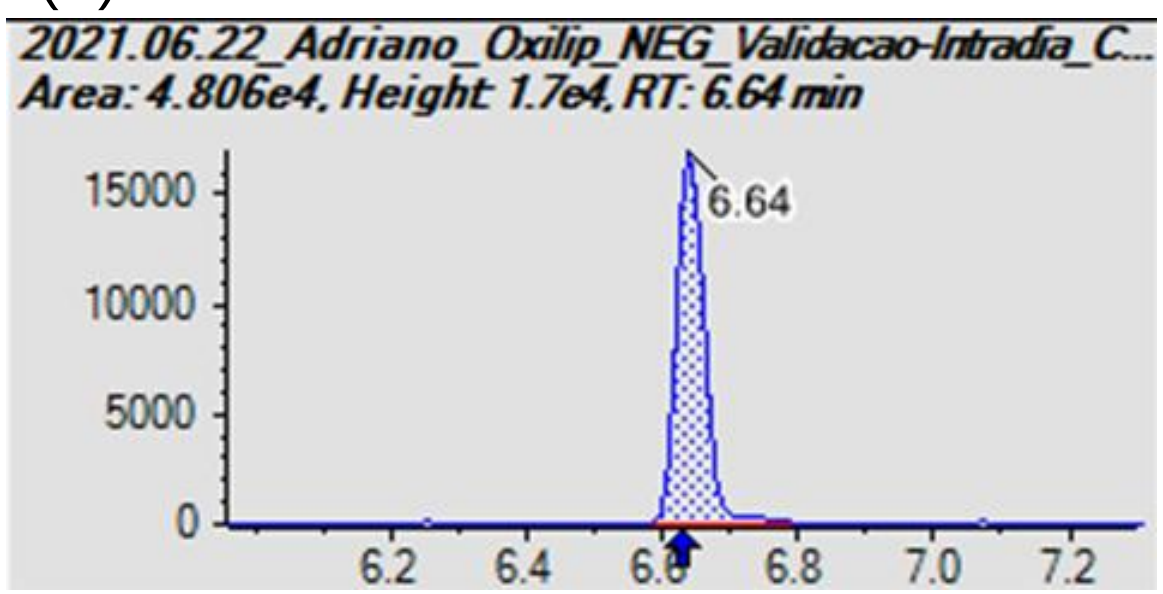

15(S)-HETrE

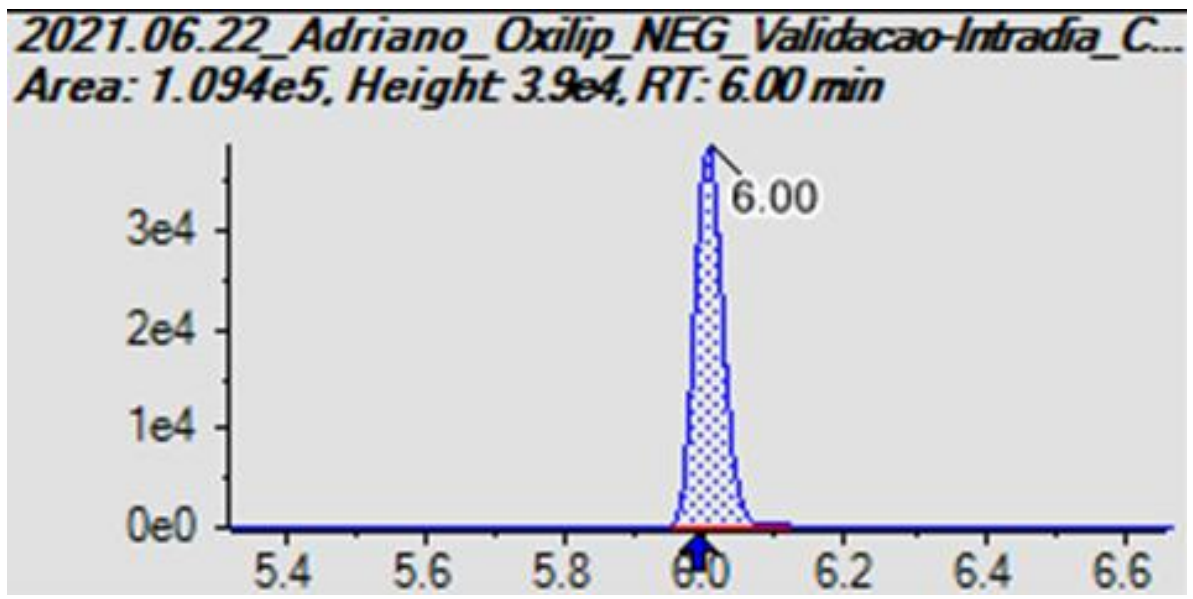

2,3-dinor-8-iso-Prostaglandin F2α

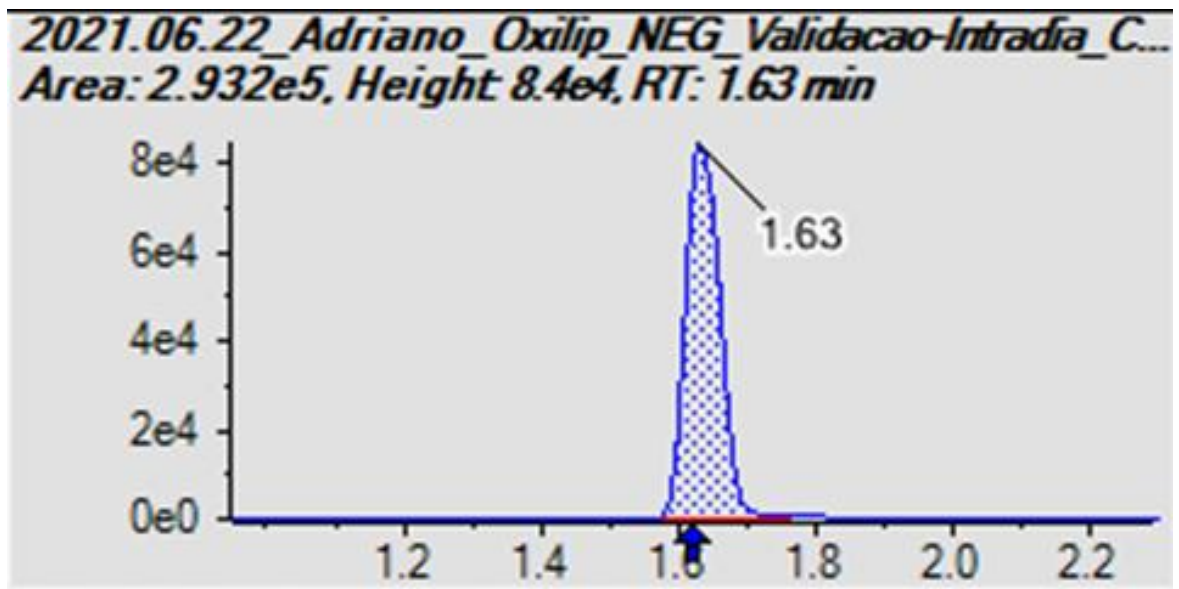

10-Nitrooleate

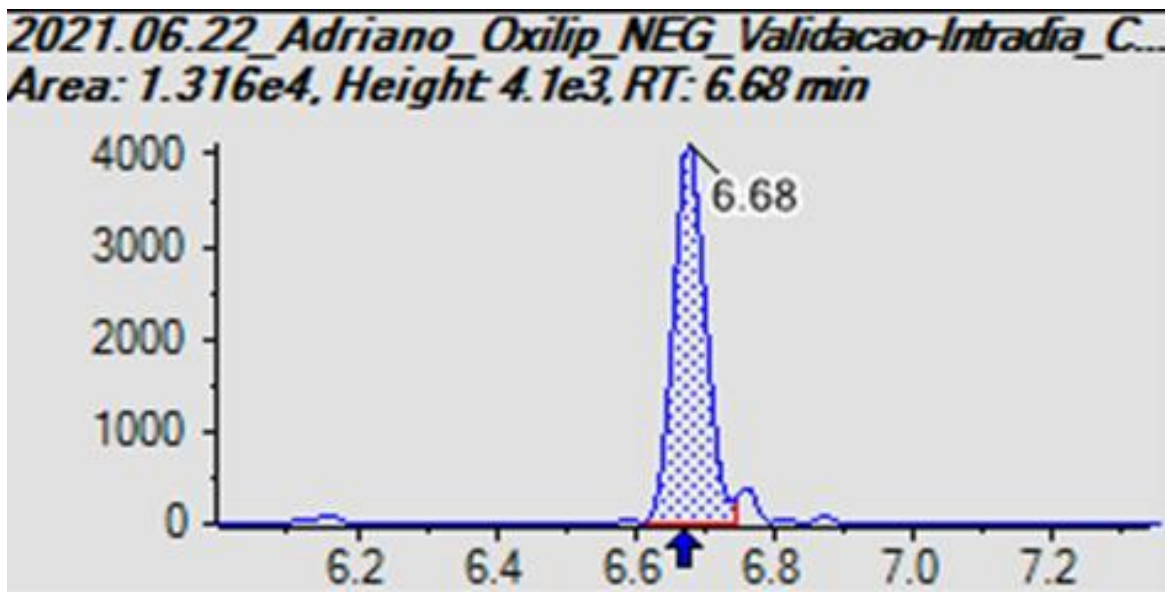

9-Nitrooleate

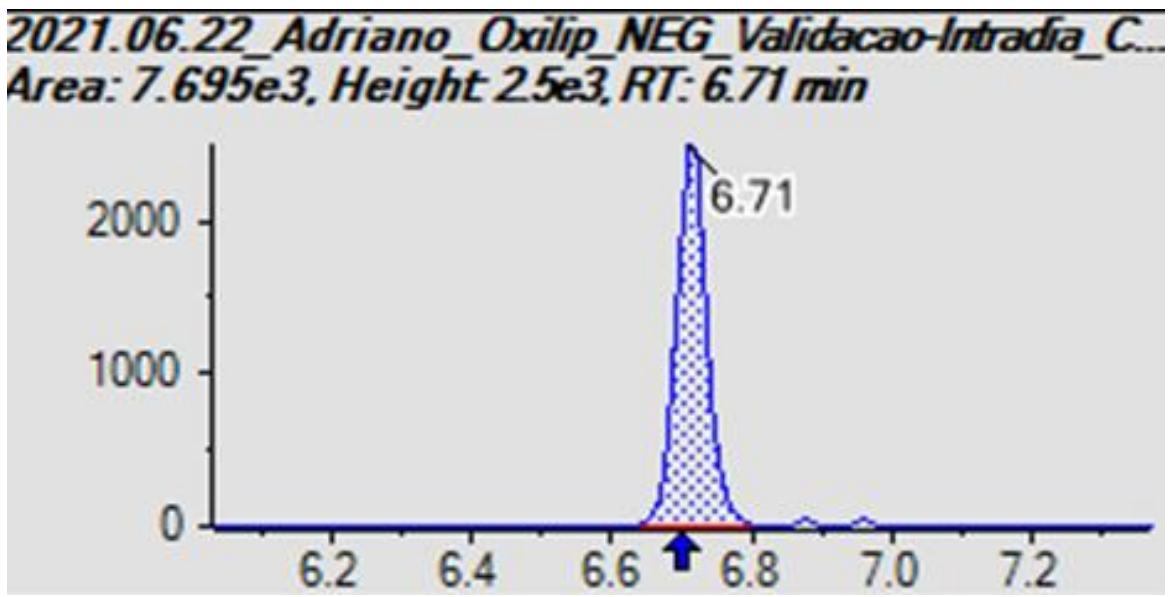

Tetranor-PGDM

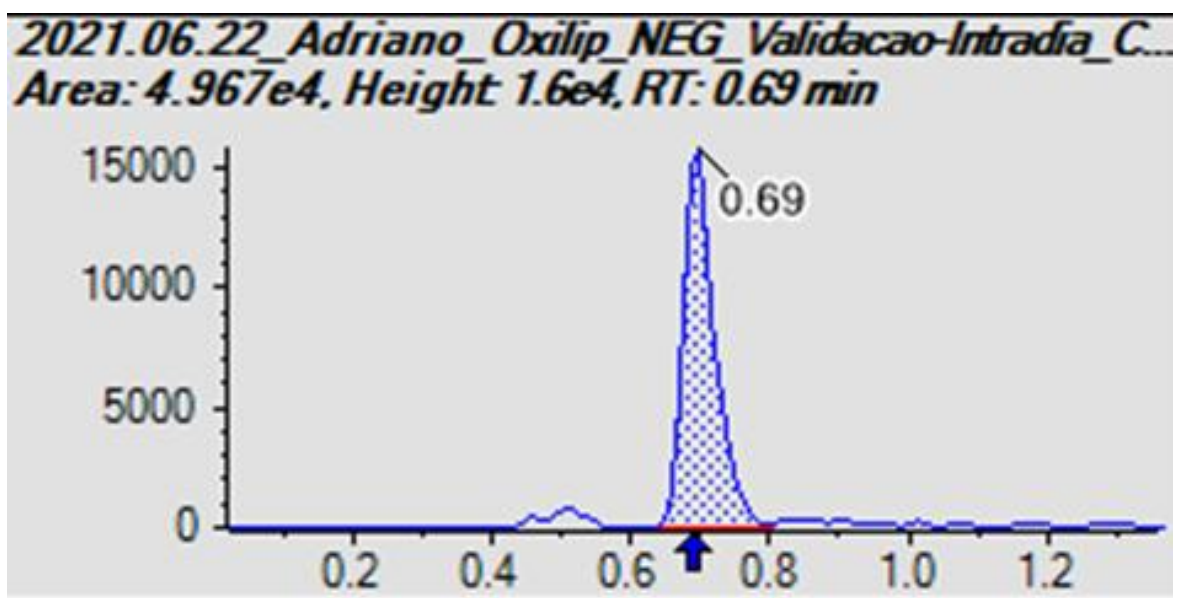

12-oxo Leukotriene B4

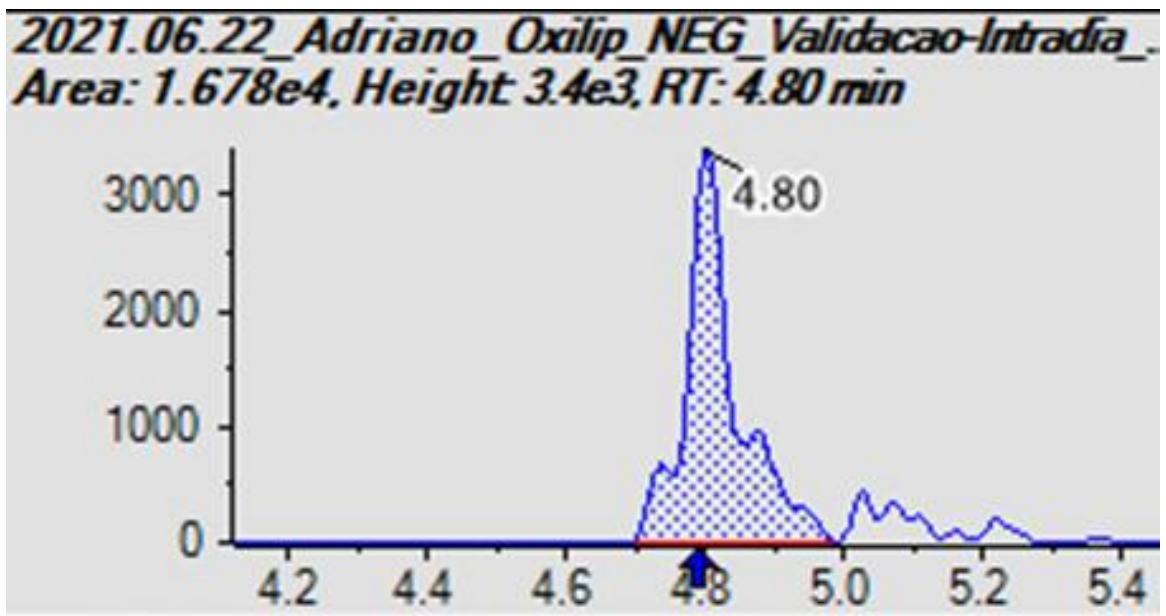

Prostaglandin J2

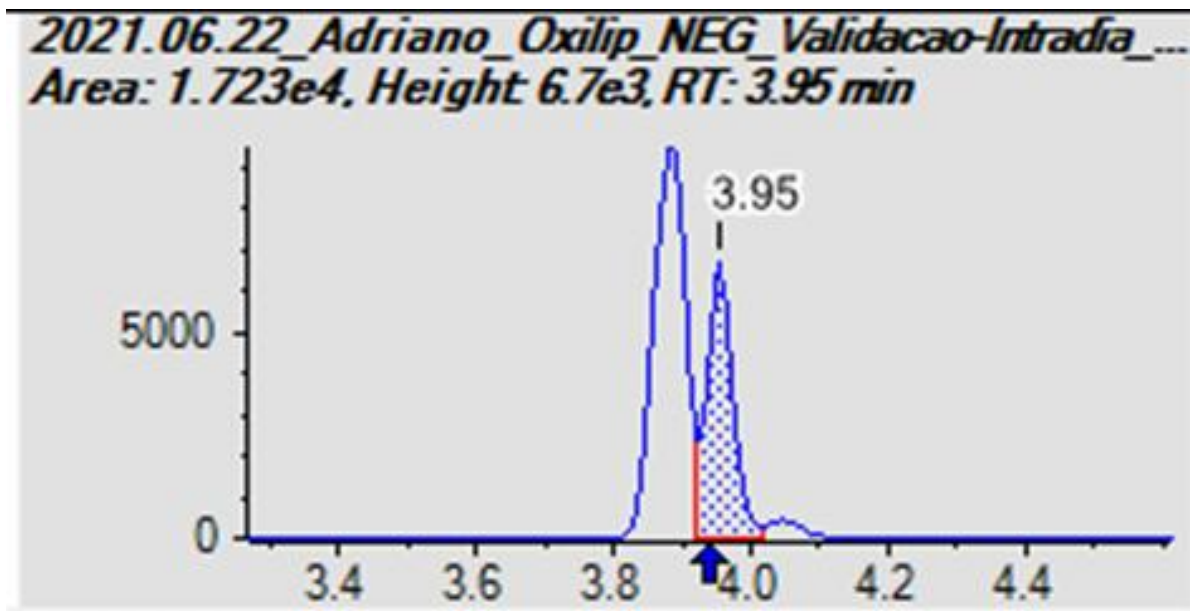

Prostaglandin B2

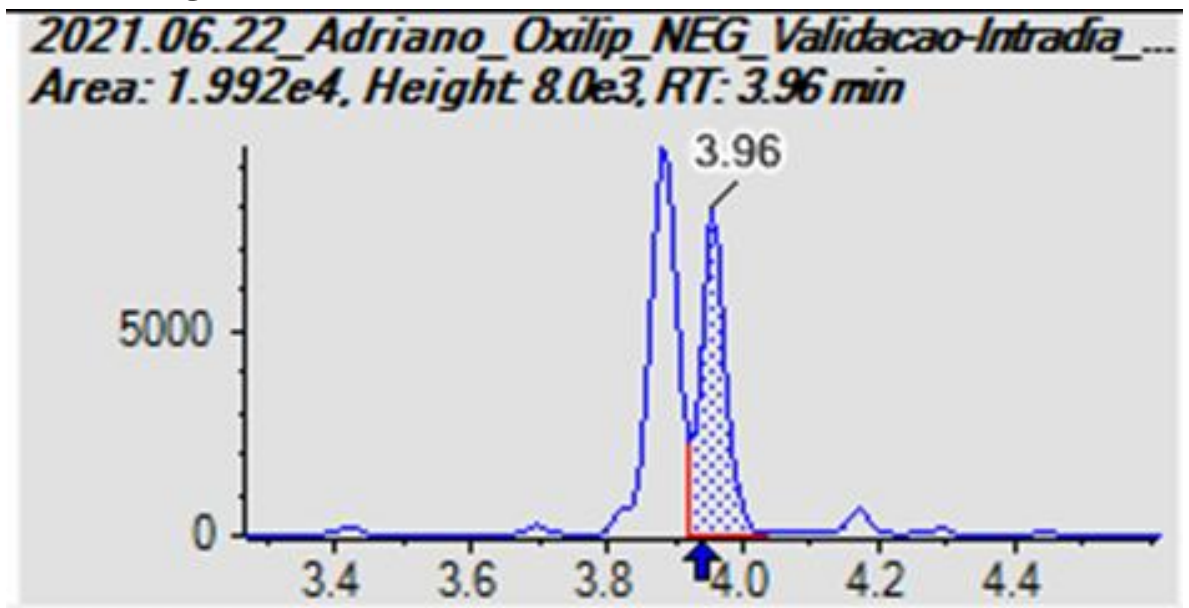

15-deoxy-Δ12,14-Prostaglandin D2

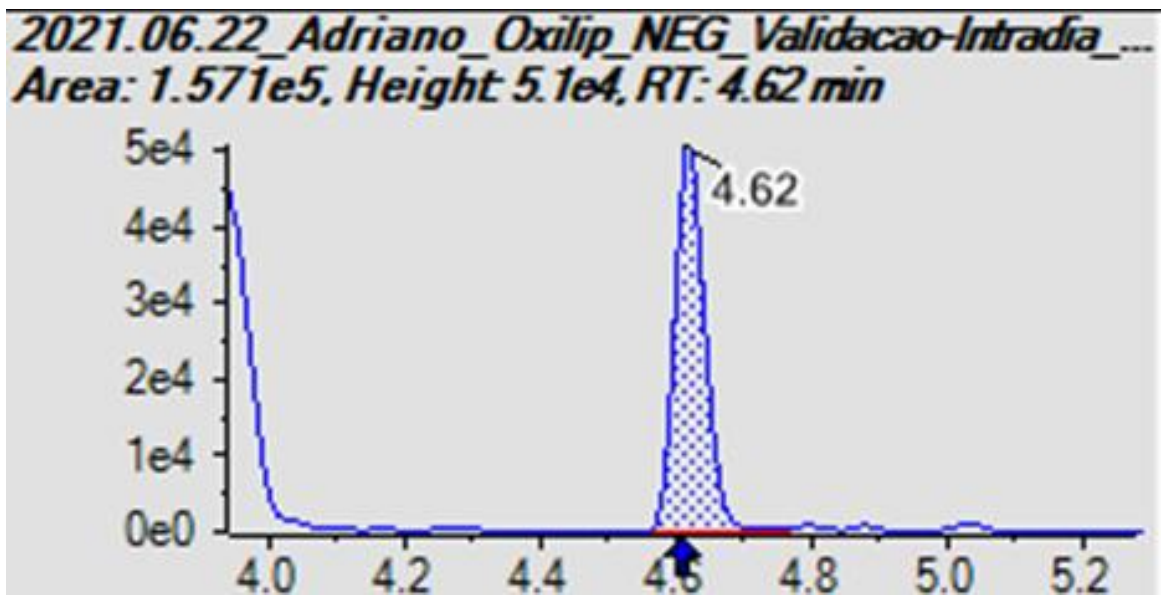

Prostaglandin A2

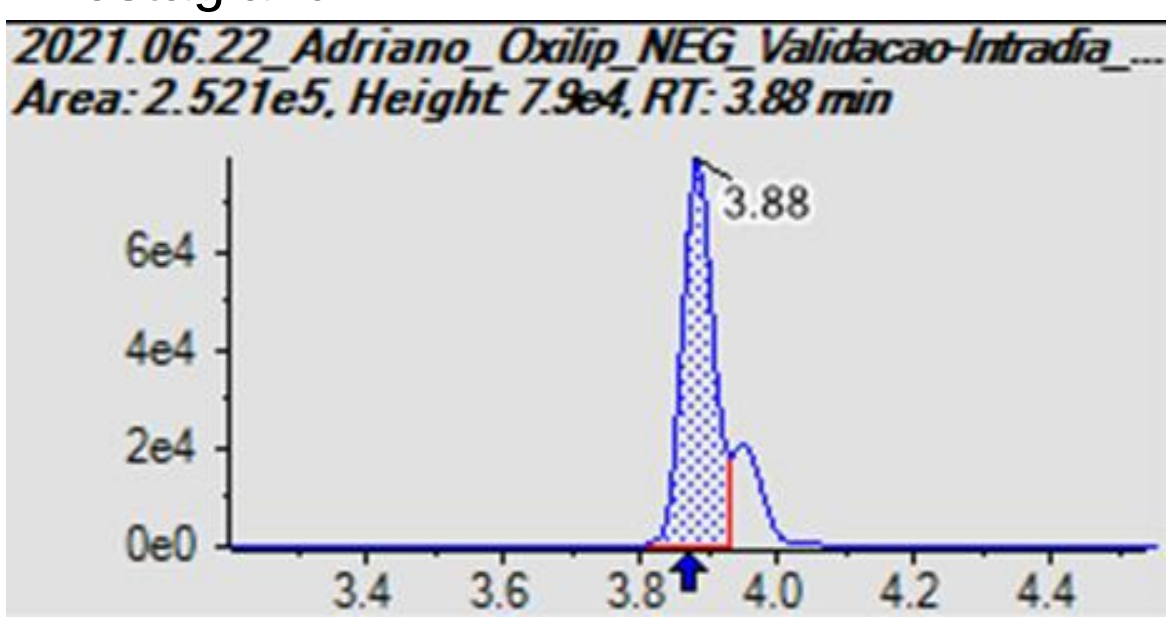

6-trans Leukotriene B4

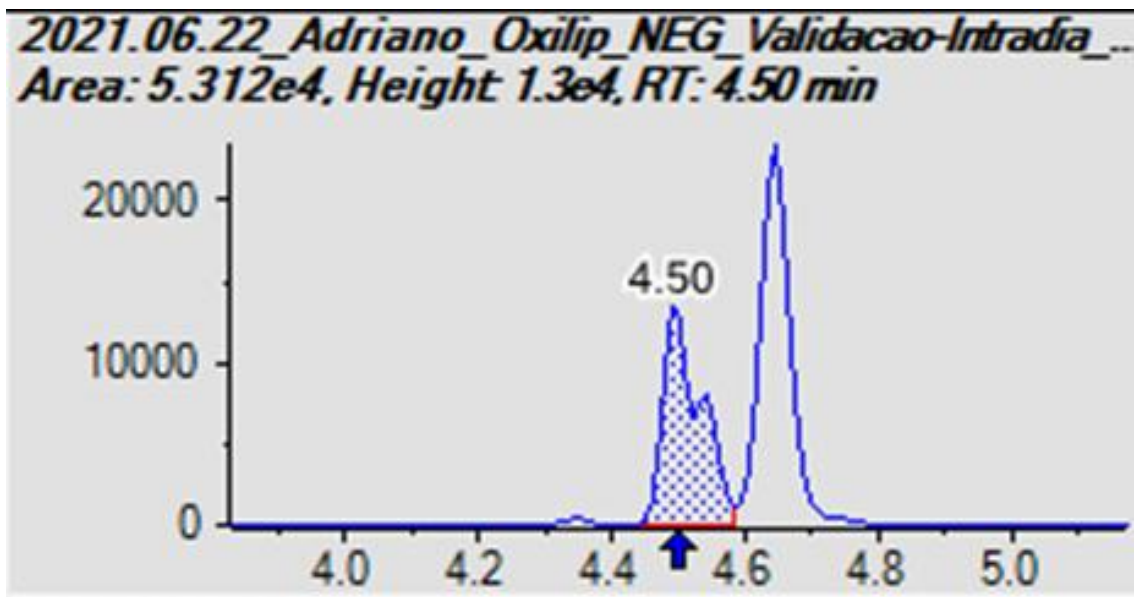

6-trans-12-epi Leukotriene B4

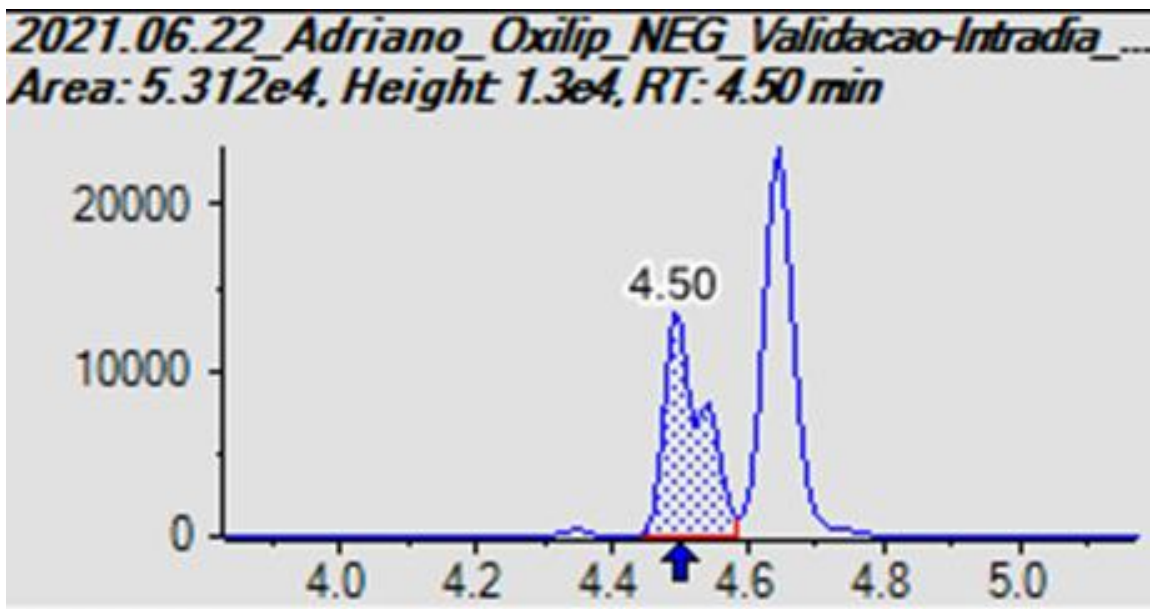

12-epi Leukotriene B4

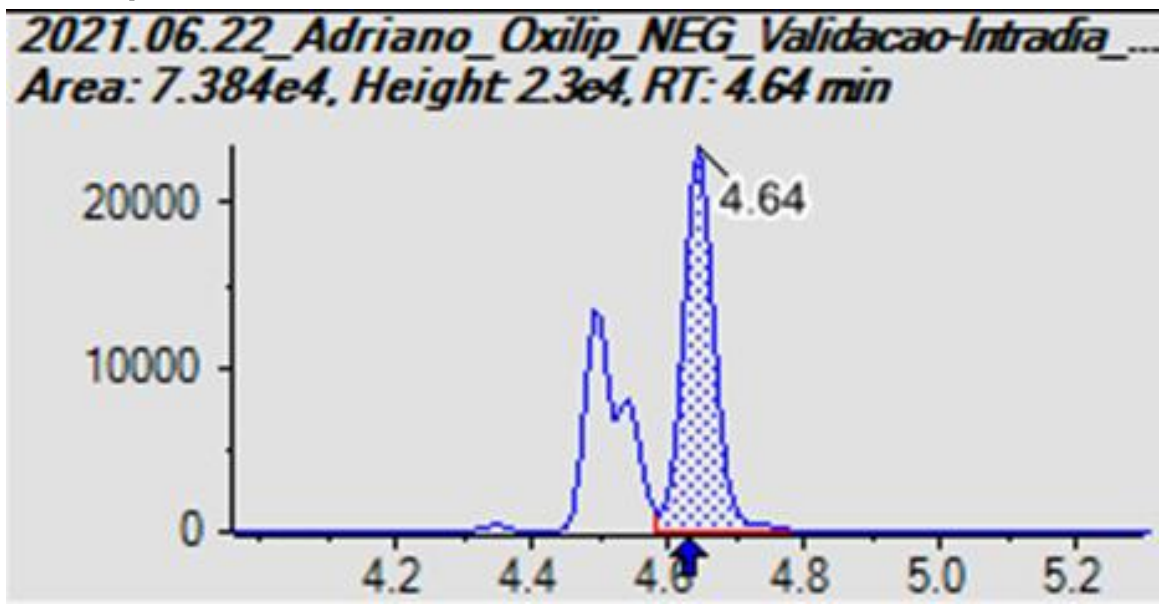

Leukotriene B4

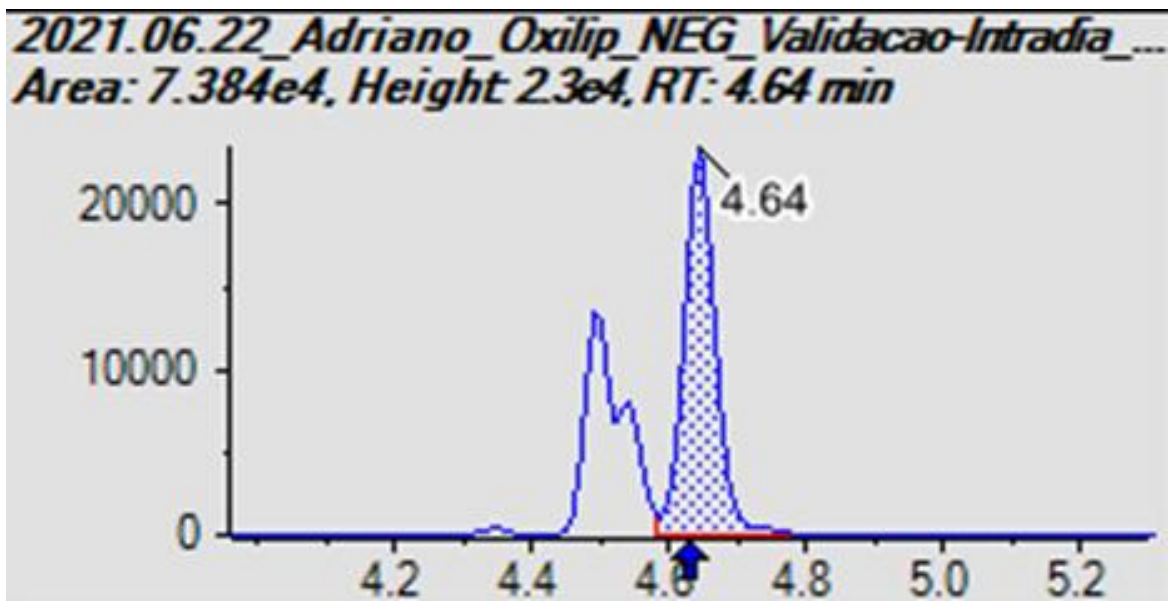

(±)14(15)DiHETE

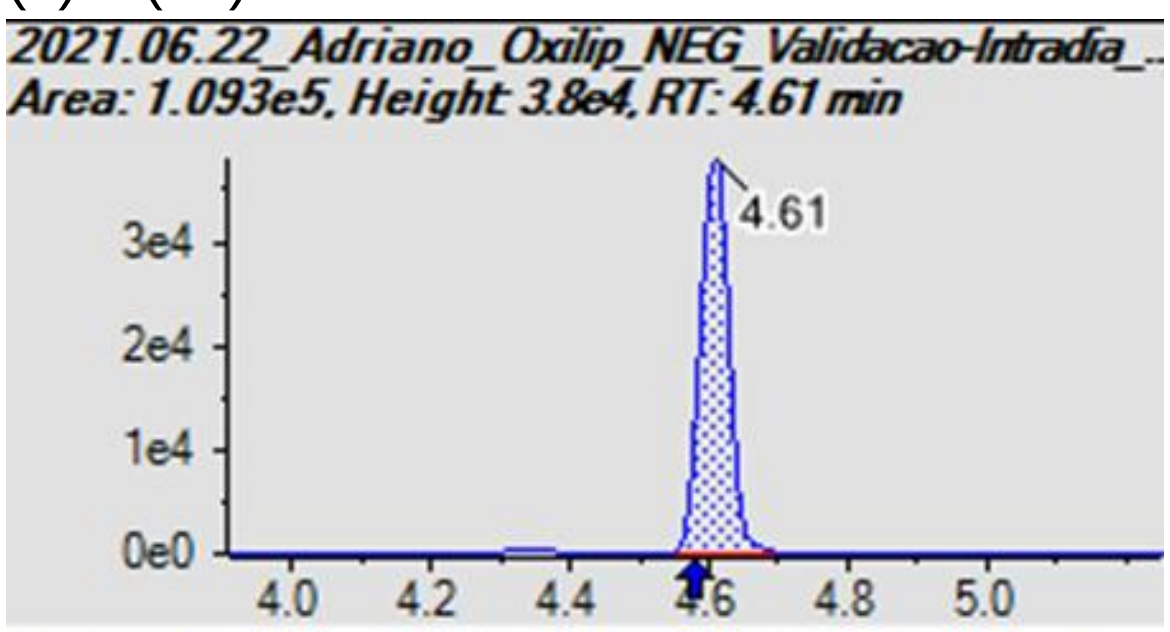

8(S),15(S)-DiHETE

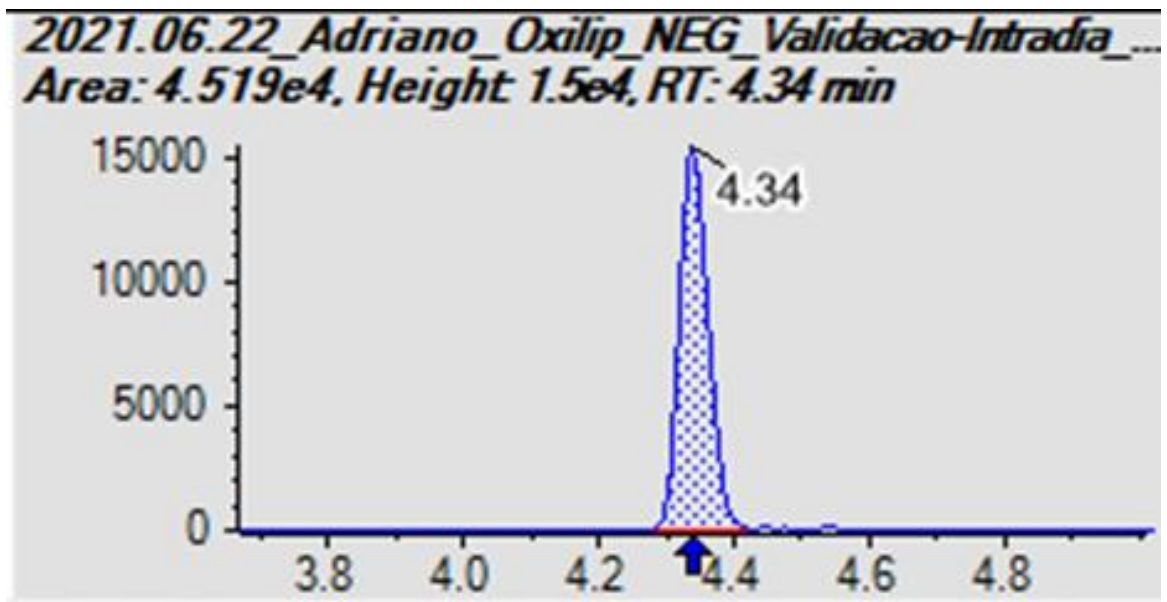

(±)8(9)-DiHET

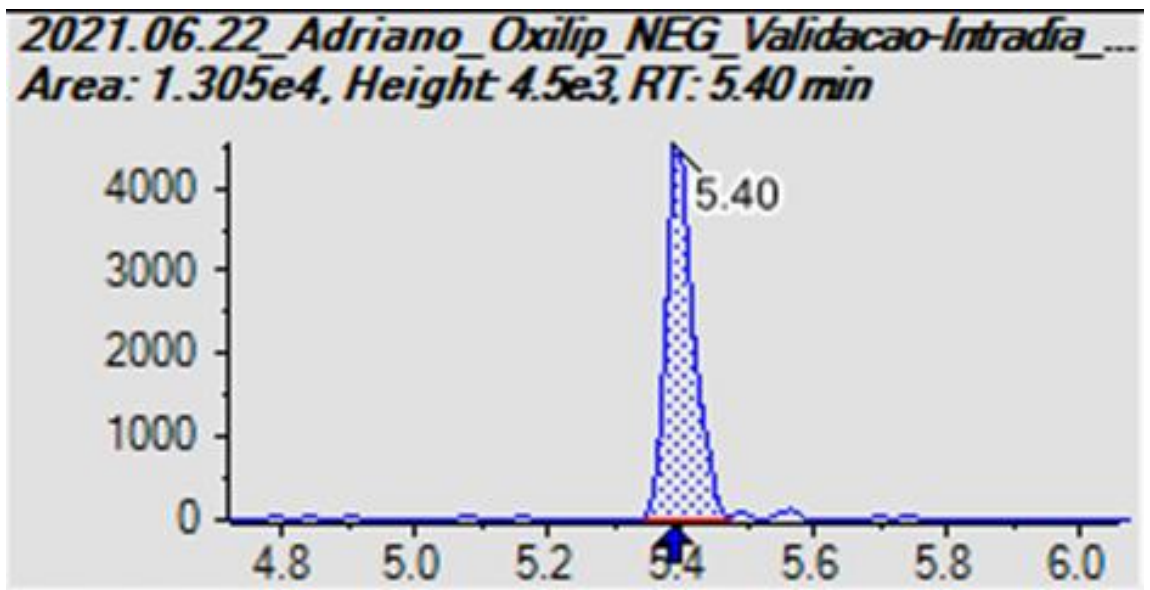

17-oxo-7(Z),10(Z),13(Z),15(E),19(Z)-Docosahexaenoic Acid

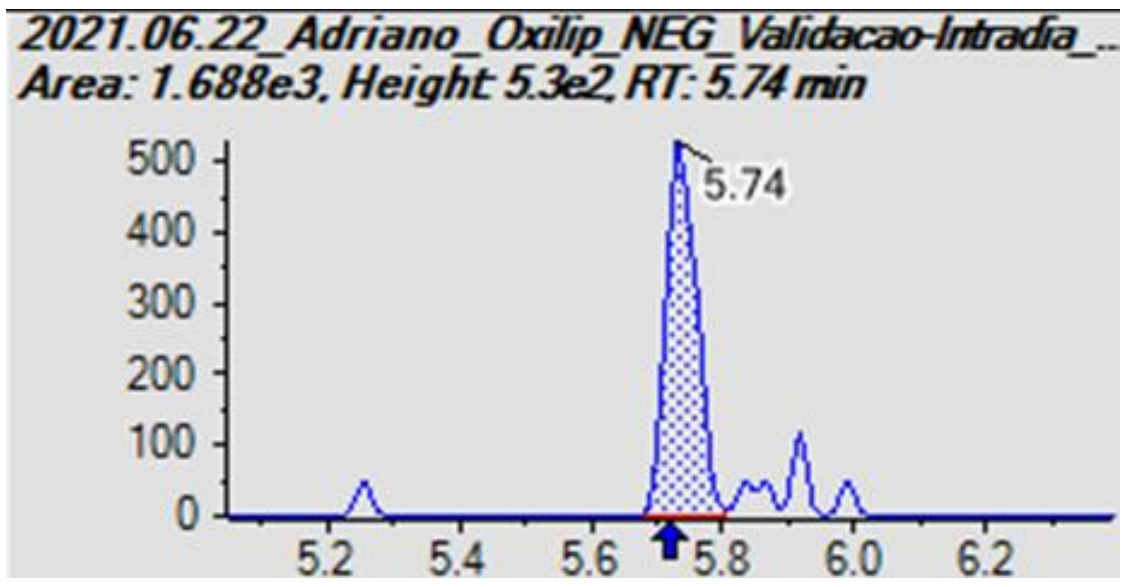

2,3dinor Thromboxane B2

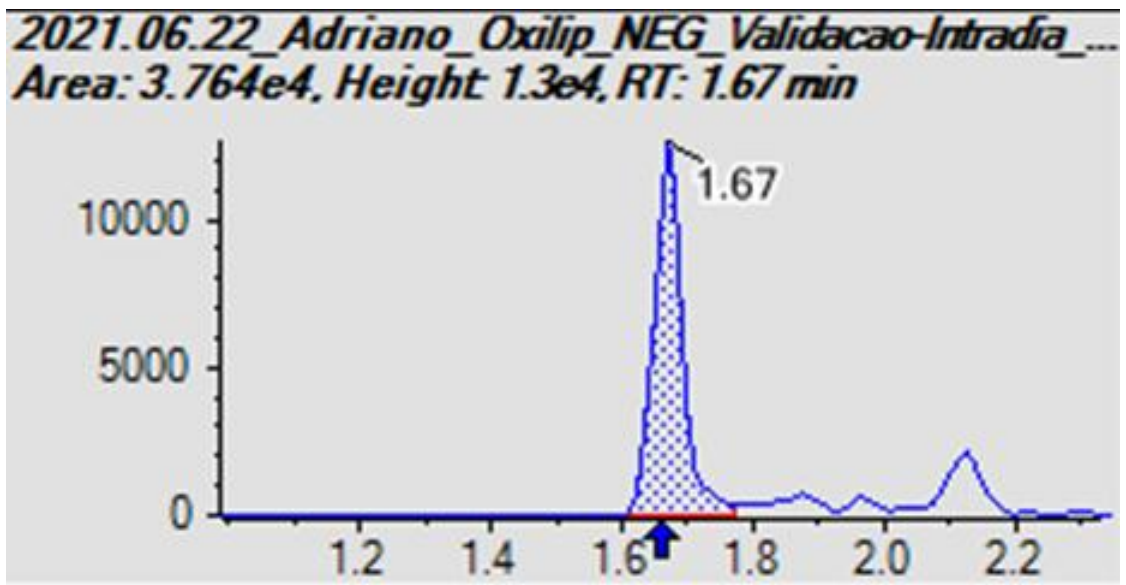

(±)4-HDHA

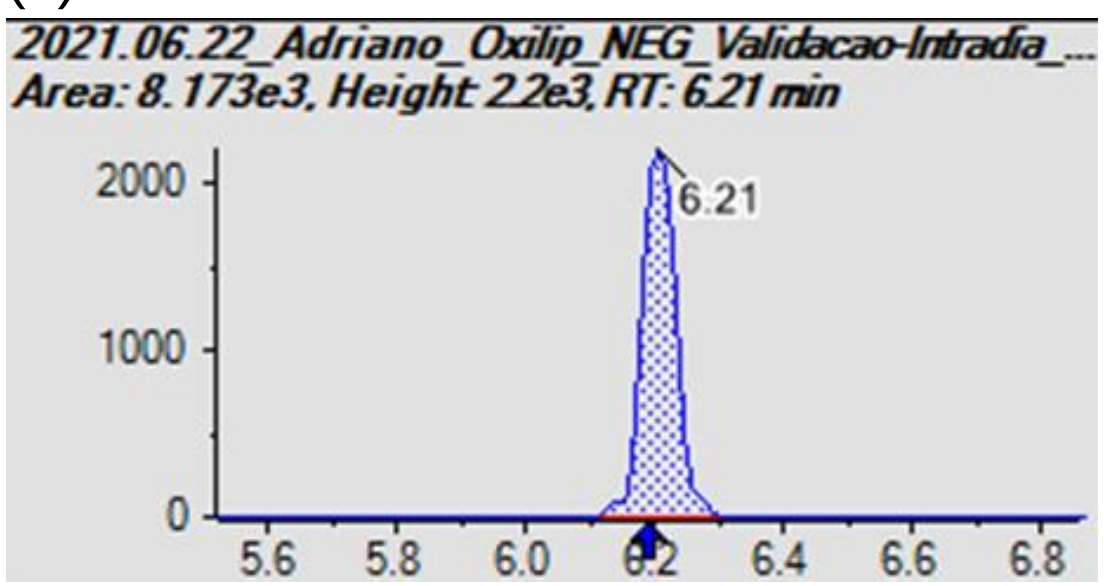

(±)8-HDHA

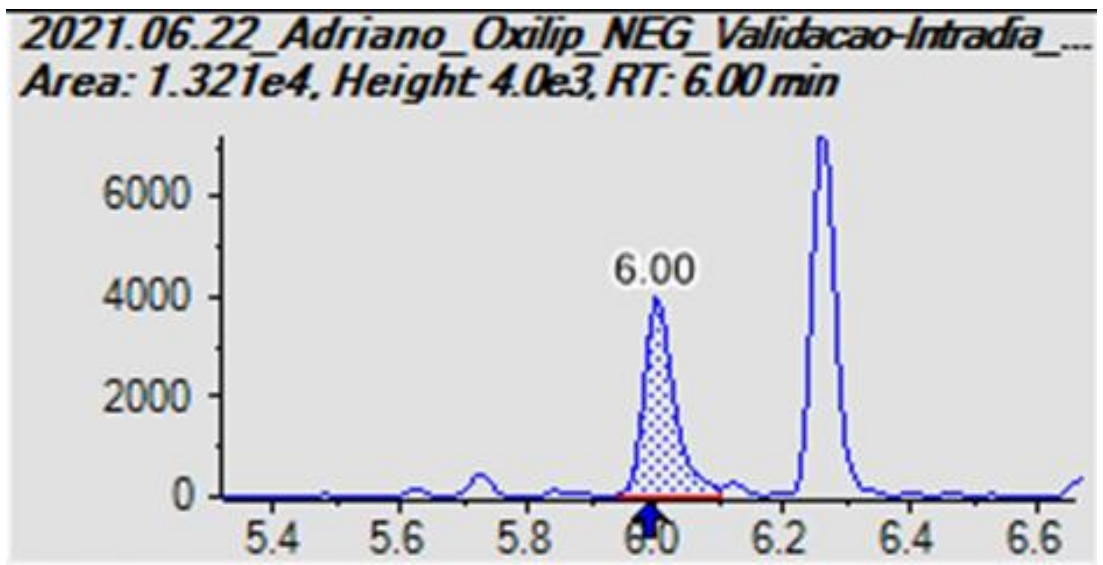

(±)7(8)-EpDPA

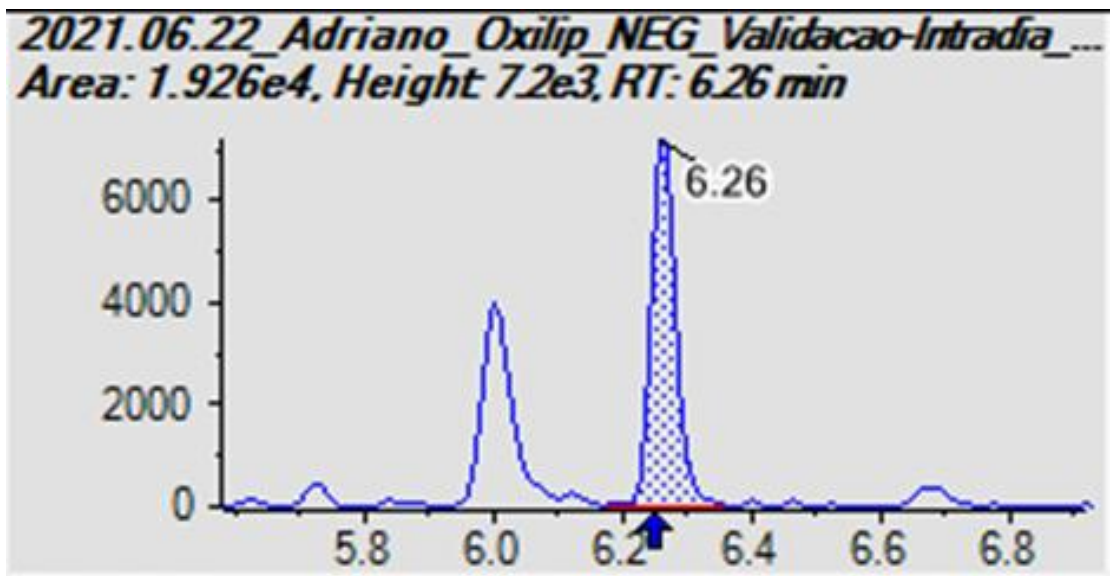

(±)7-HDHA

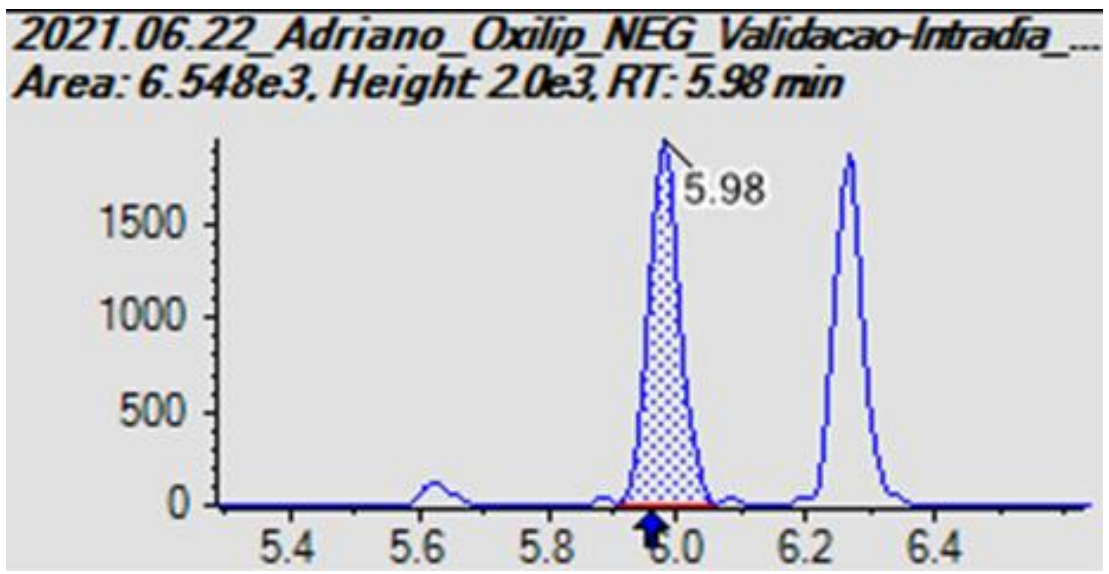

10-HDHA

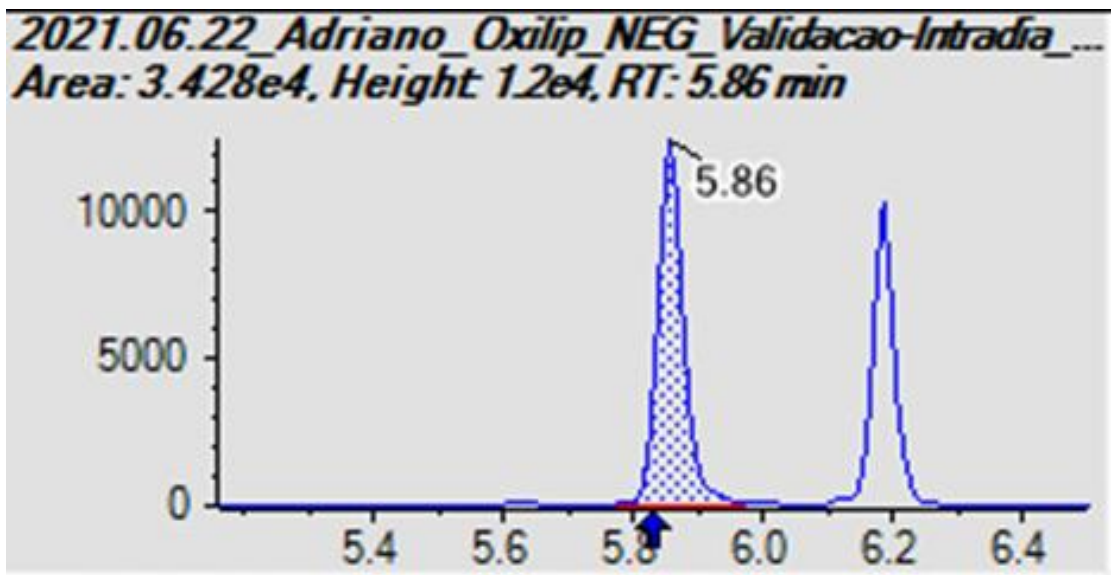

(±)10(11)-EpDPA

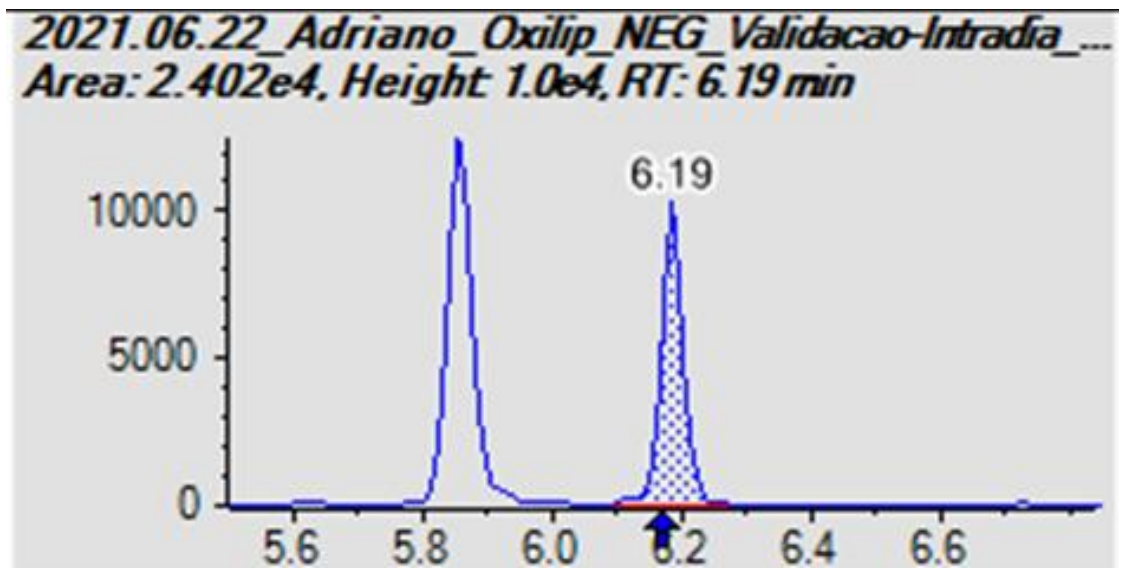

(±)11-HDHA

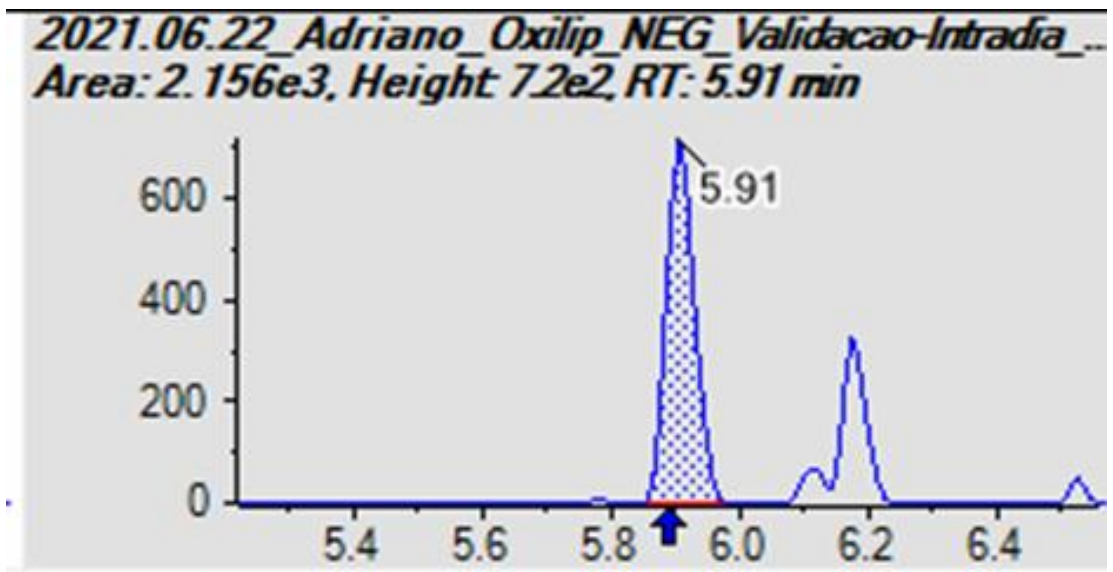

(±)13-HDHA

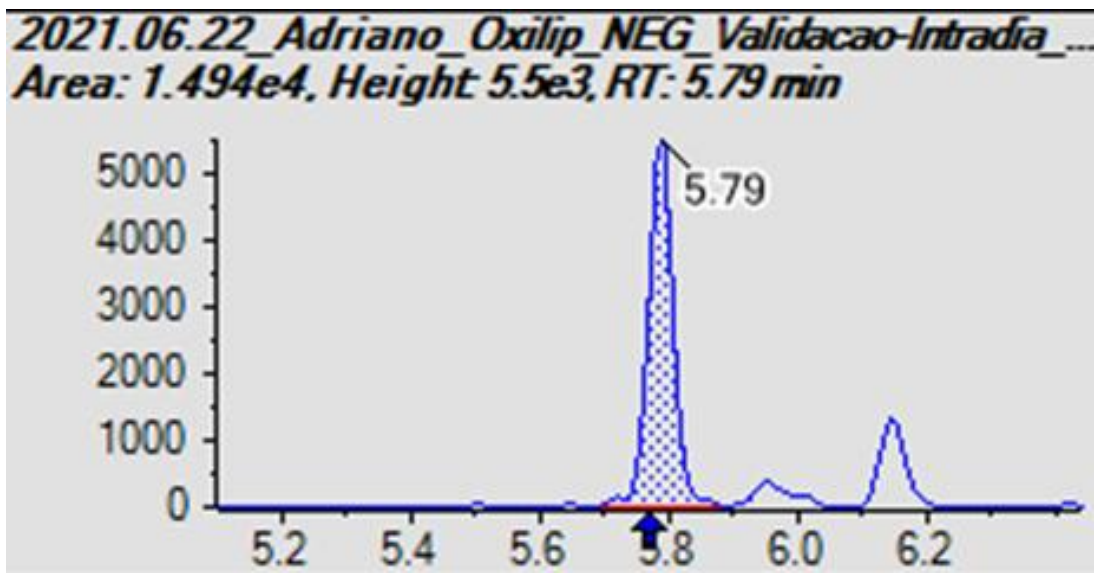

(±)13(14)-EpDPA

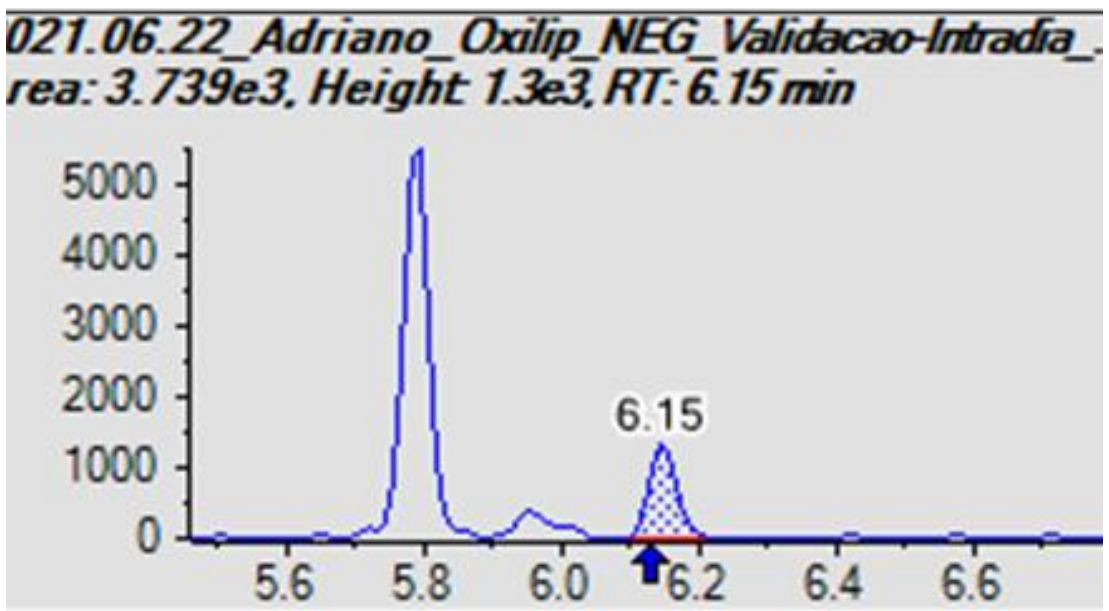

(±)16(17)-EpDPA

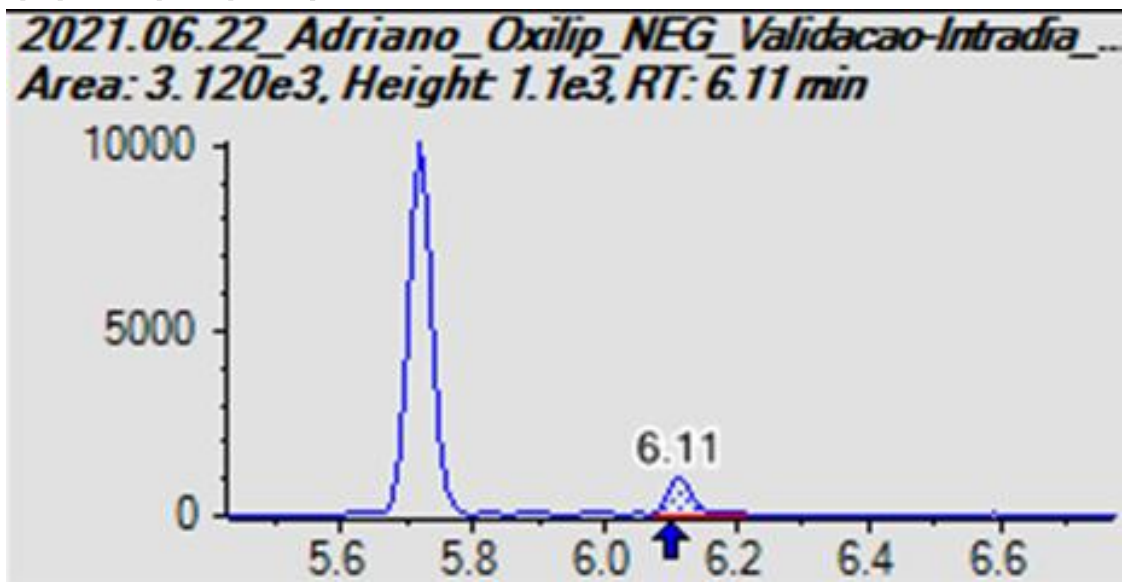

(±)14-HDHA

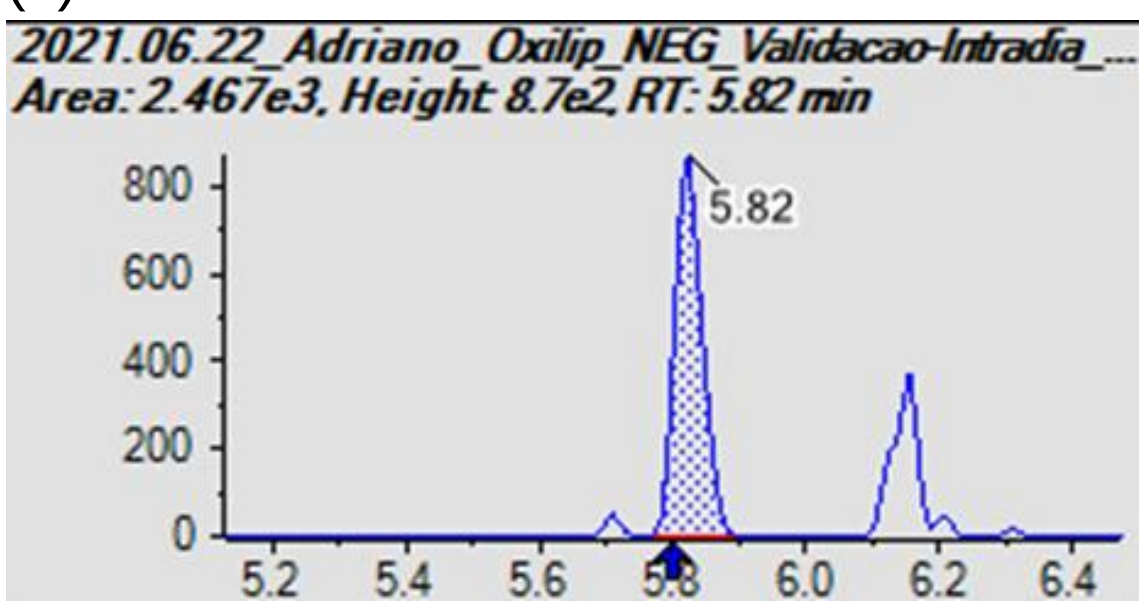

(±)20-HDHA

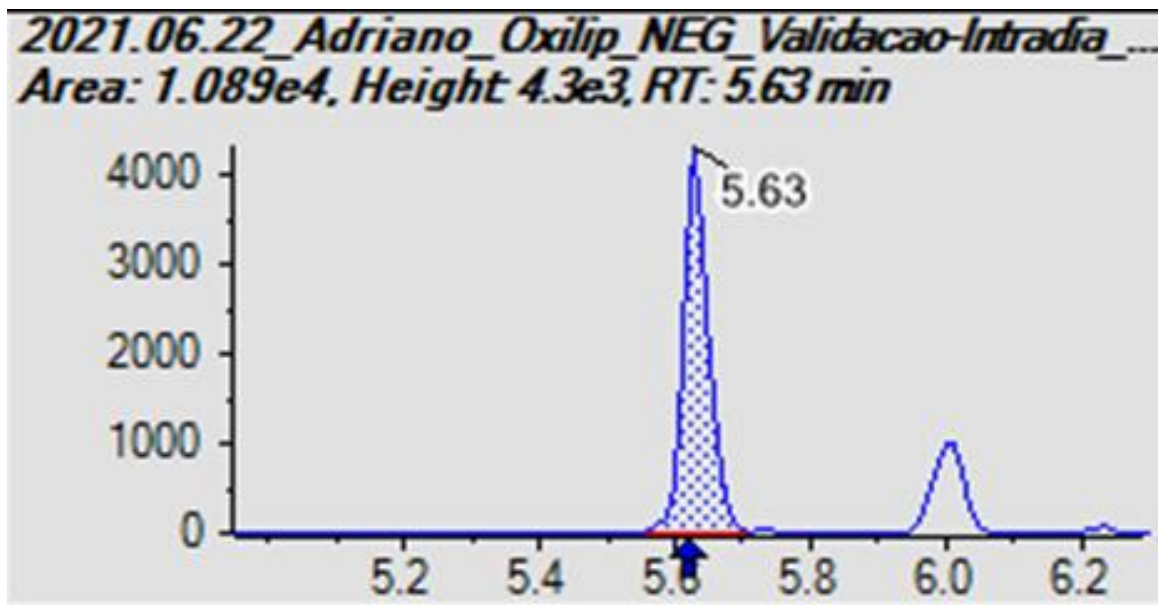

(±)19(20)-EpDPA

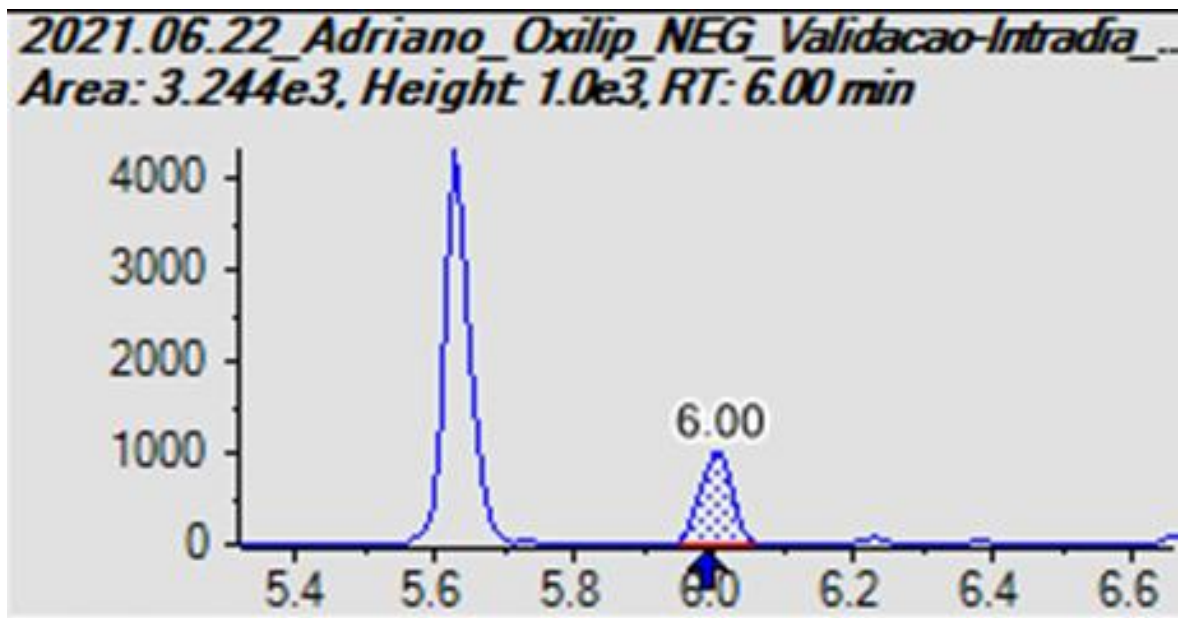

17-oxo-7(Z),10(Z),13(Z),15(E),19(Z)-Docosapentaenoic Acid

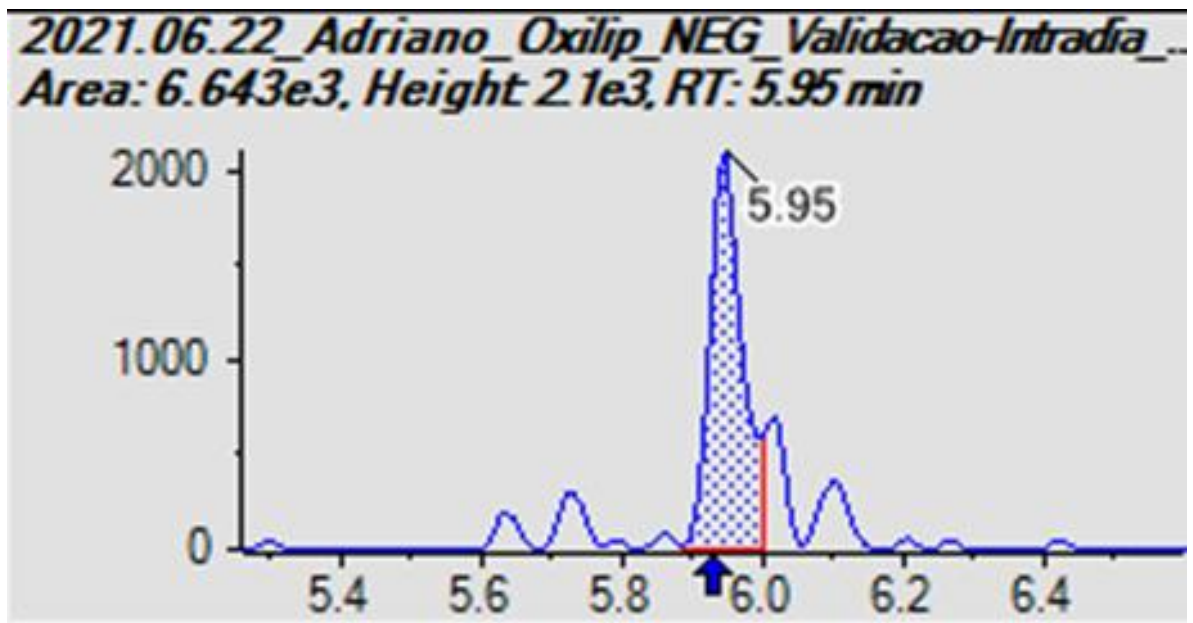

19-HDoHE

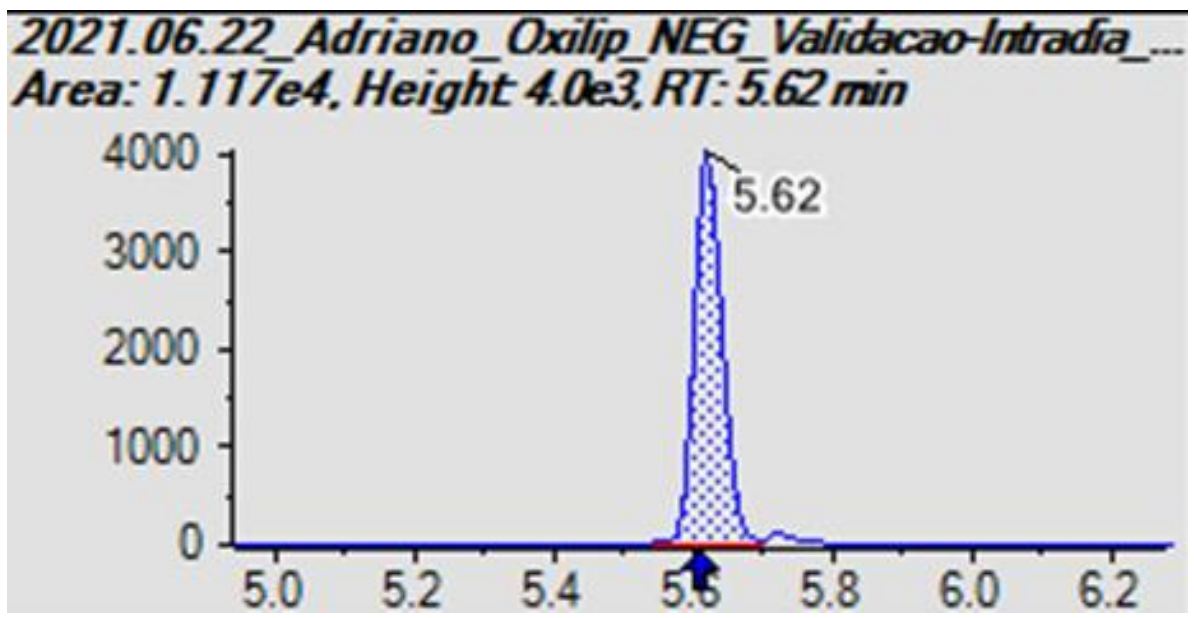

16 and 17-HDoHE

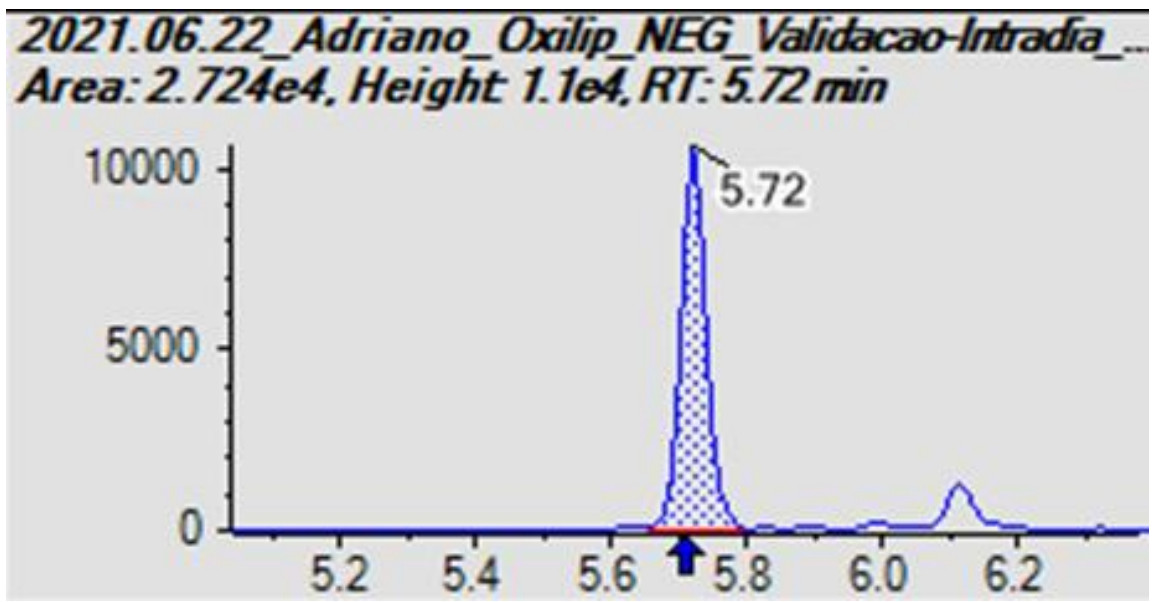

15(R)-Lipoxin A4

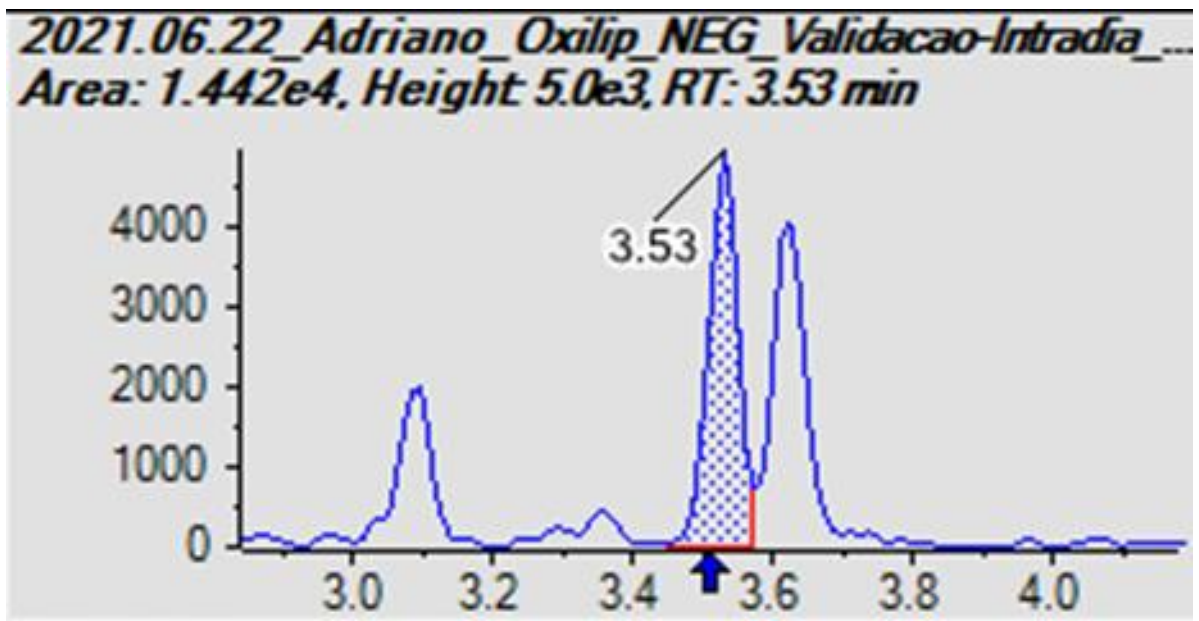

Lipoxin A5

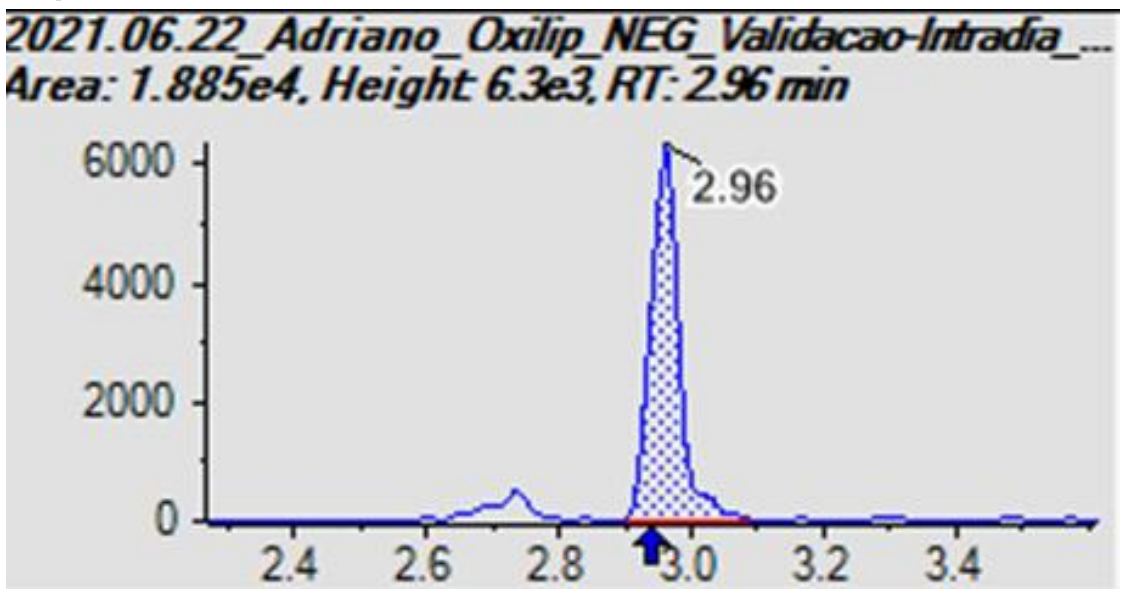

6(S)-Lipoxin A4

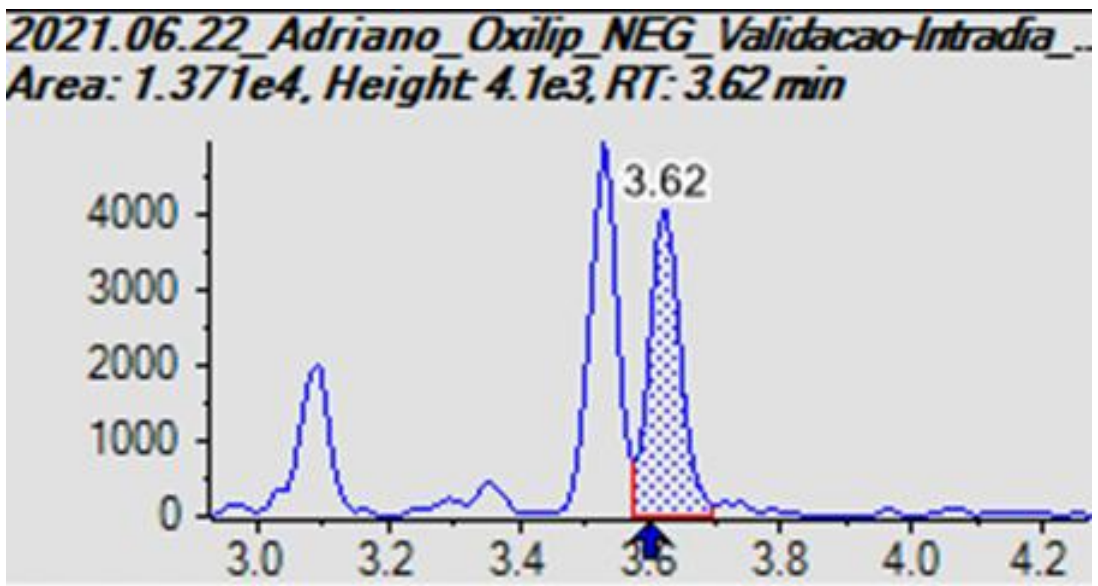

Resolvin E1

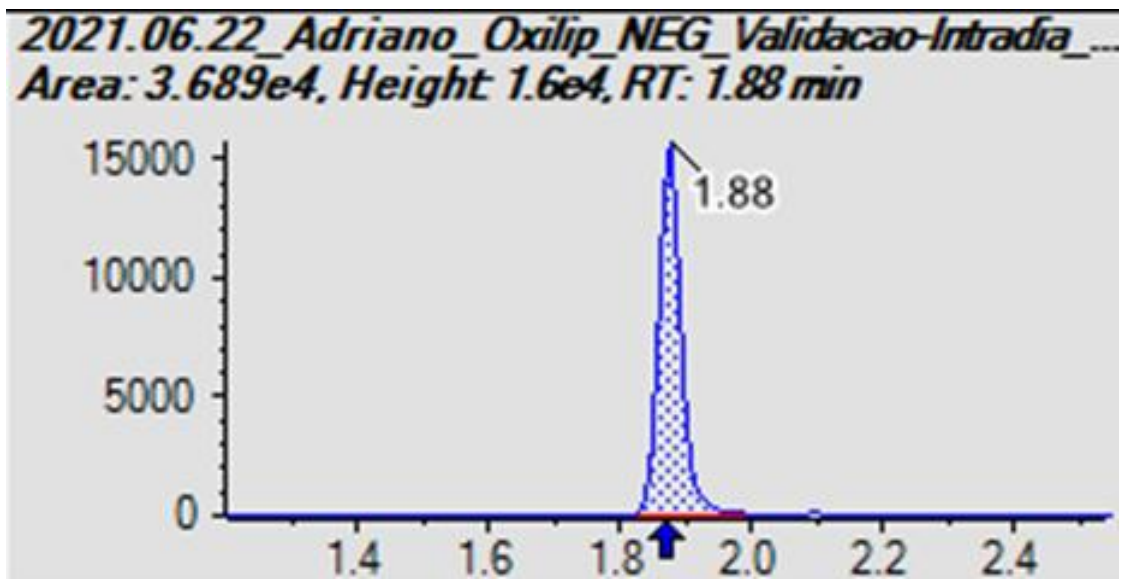

13,14-dihydro15keto Prostaglandin E2

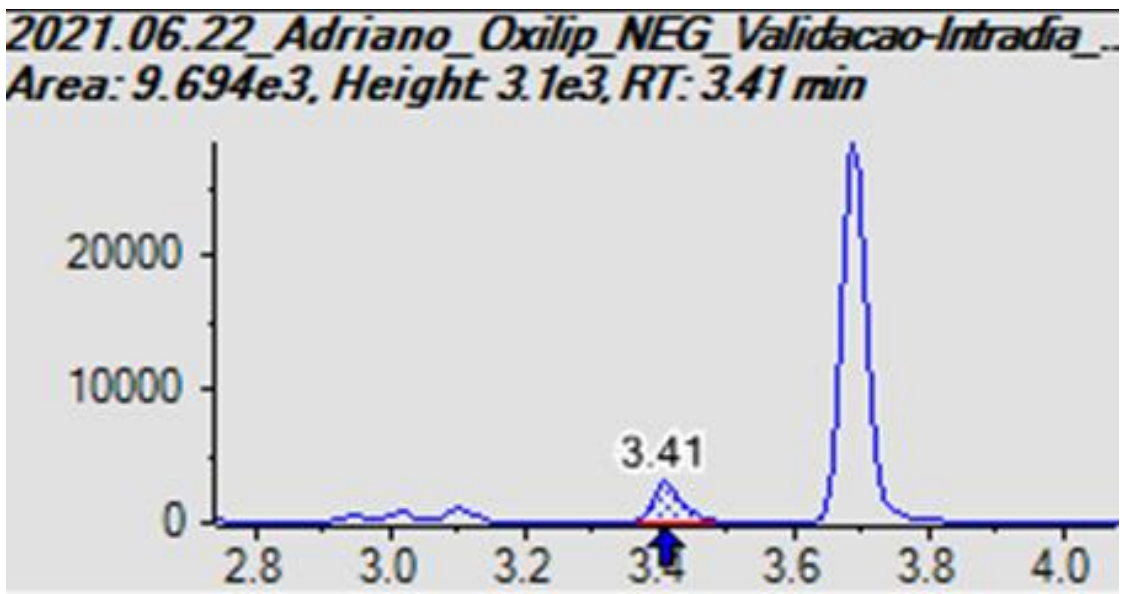

15-keto Prostaglandin E2

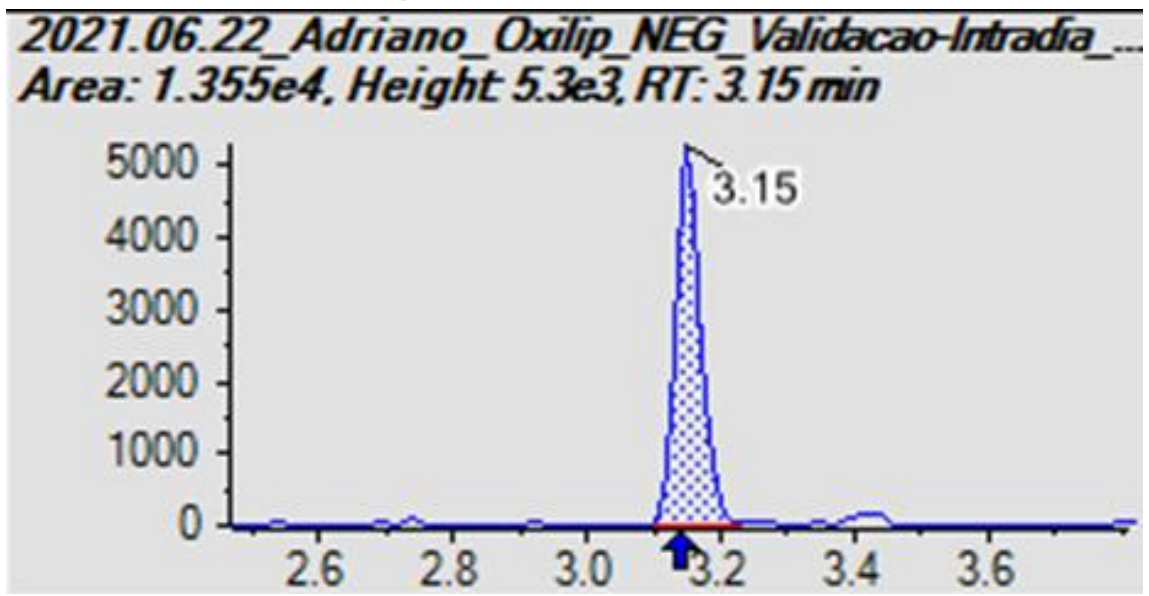

13,14-dihydro-15-keto Prostaglandin D2

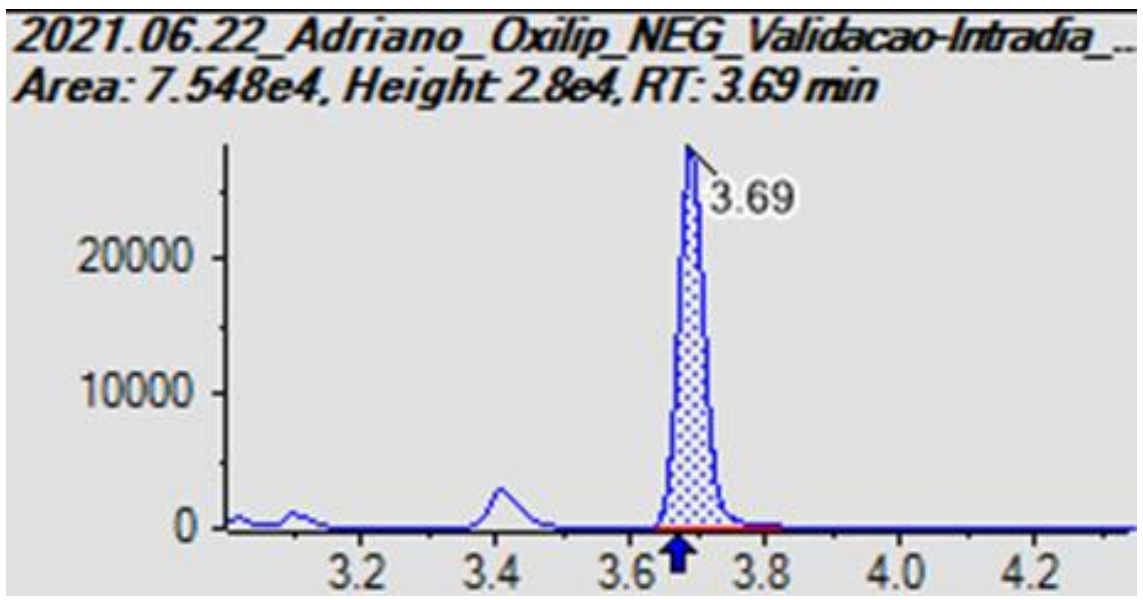

Prostaglandin E3

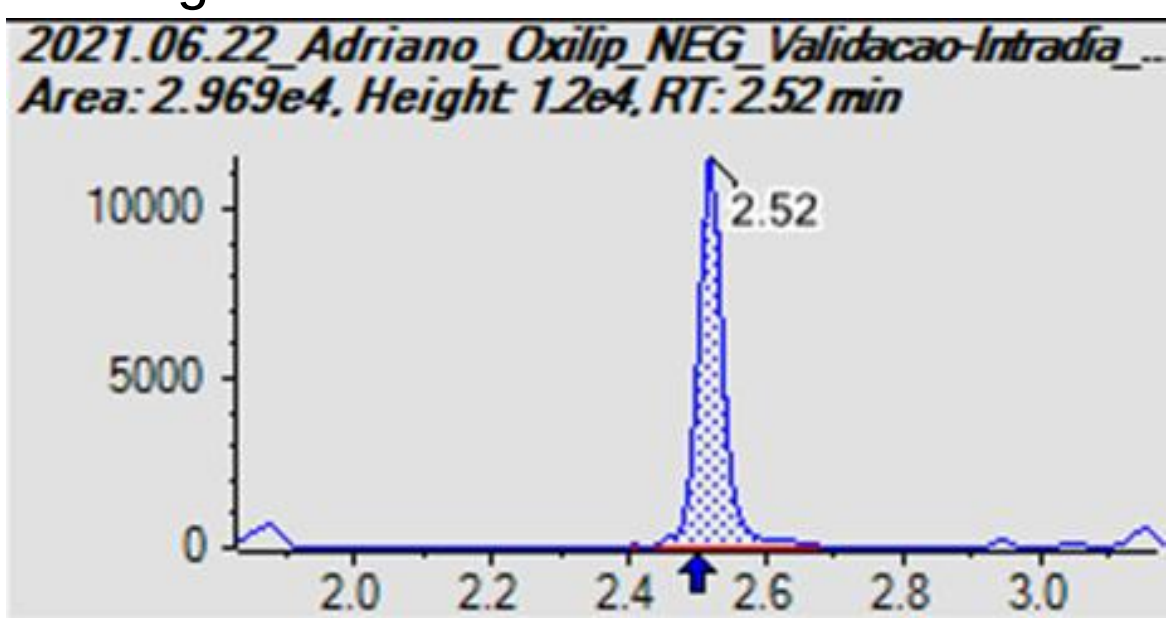

8-iso Prostaglandin F3α

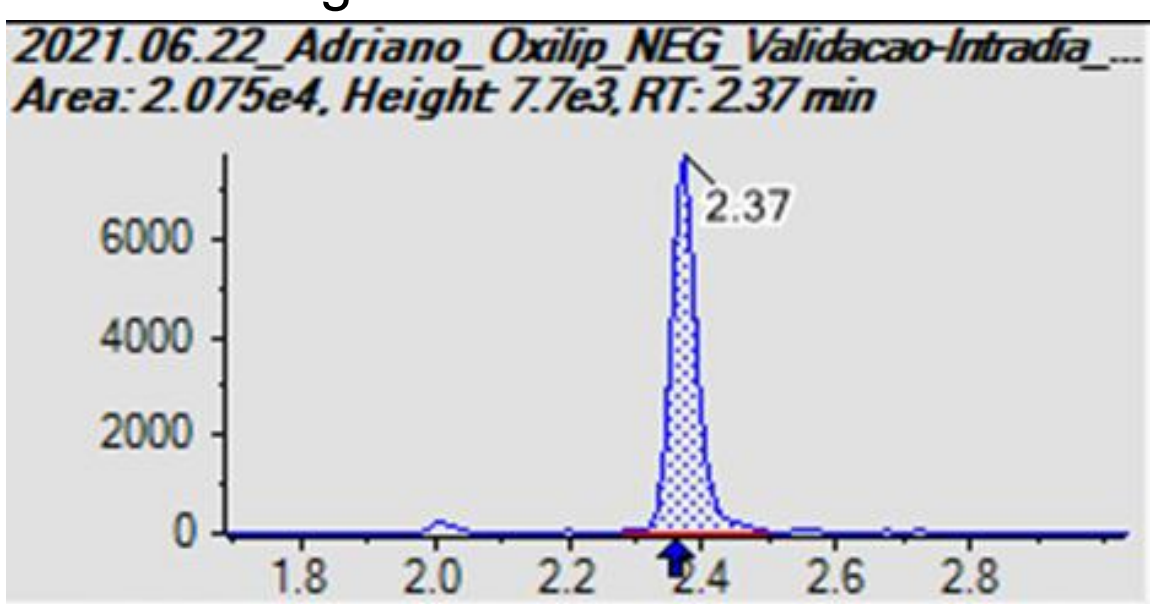

Prostaglandin F3α

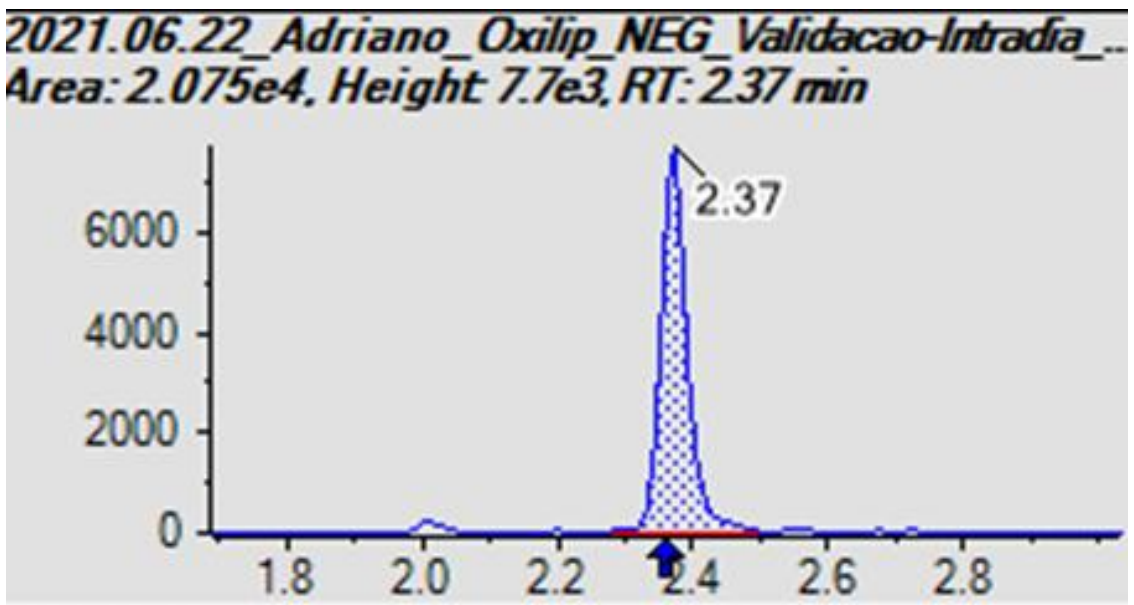

20-carboxy Leukotriene B4

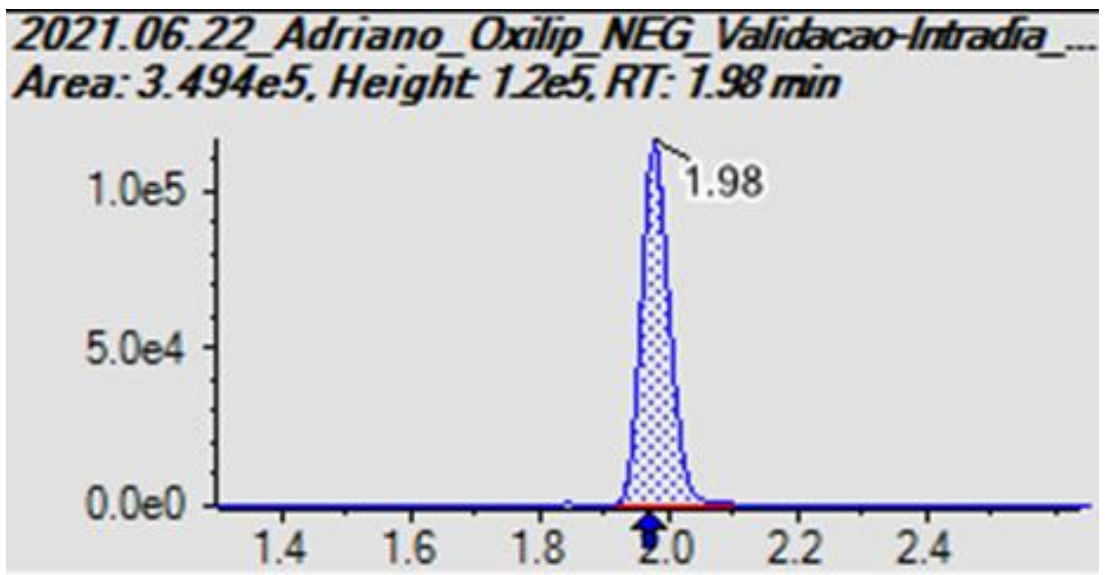

8-iso-15-keto Prostaglandin F2β

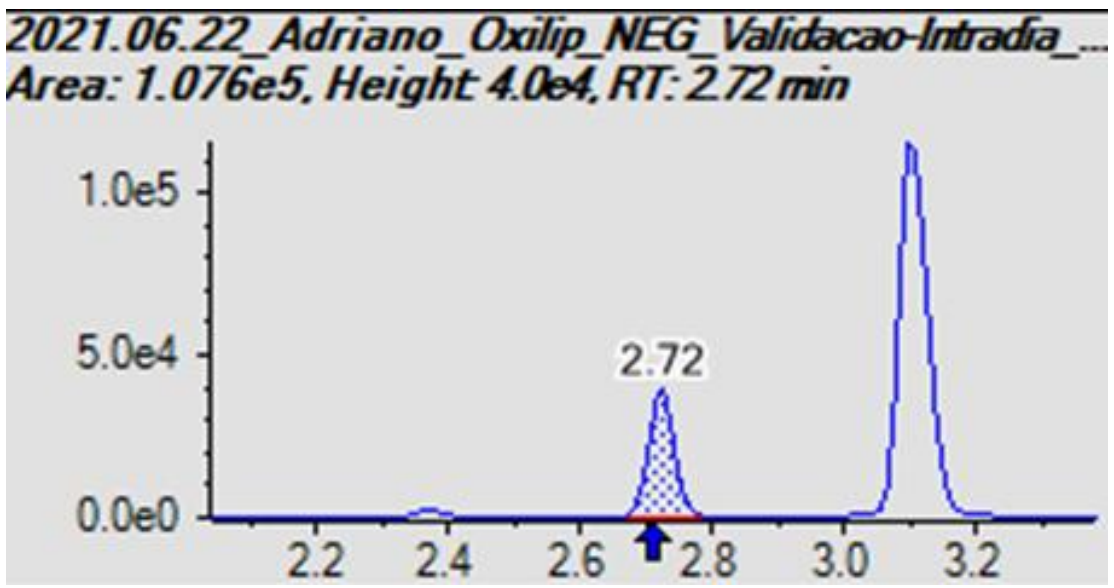

15-keto Prostaglandin F2α

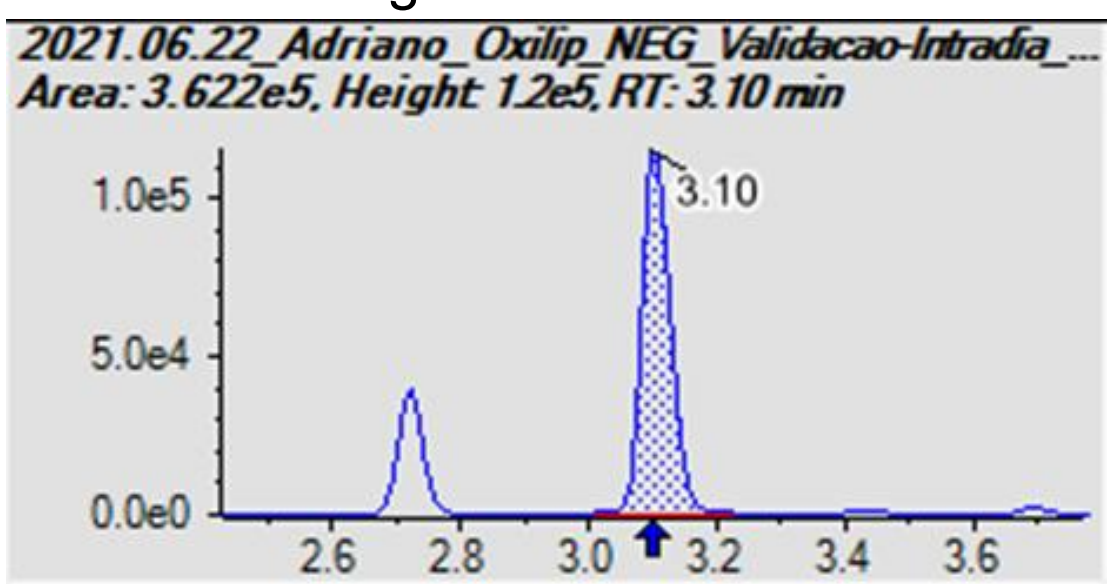

Lipoxin B4

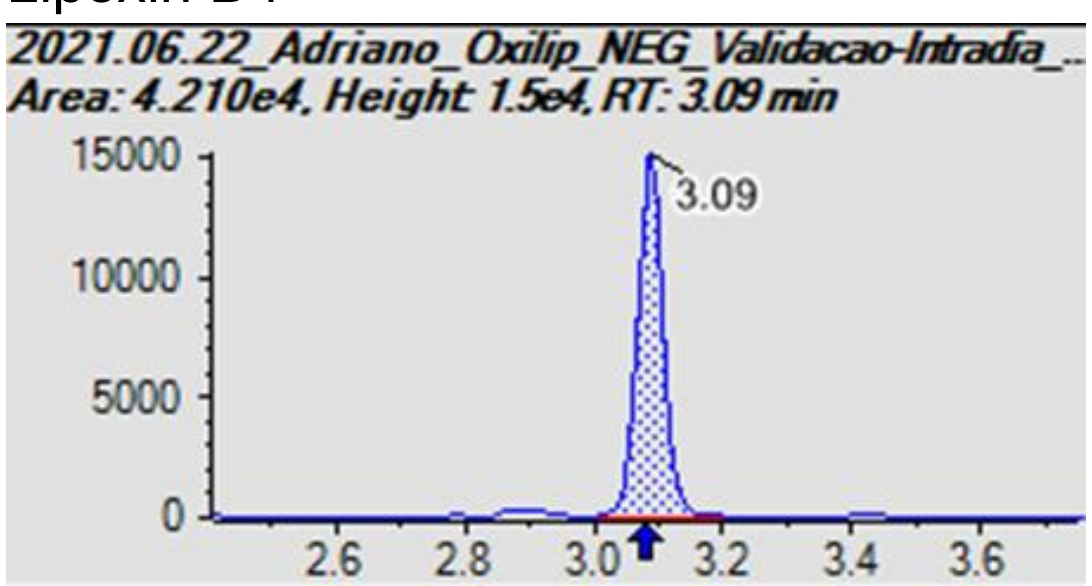

Prostaglandin E2

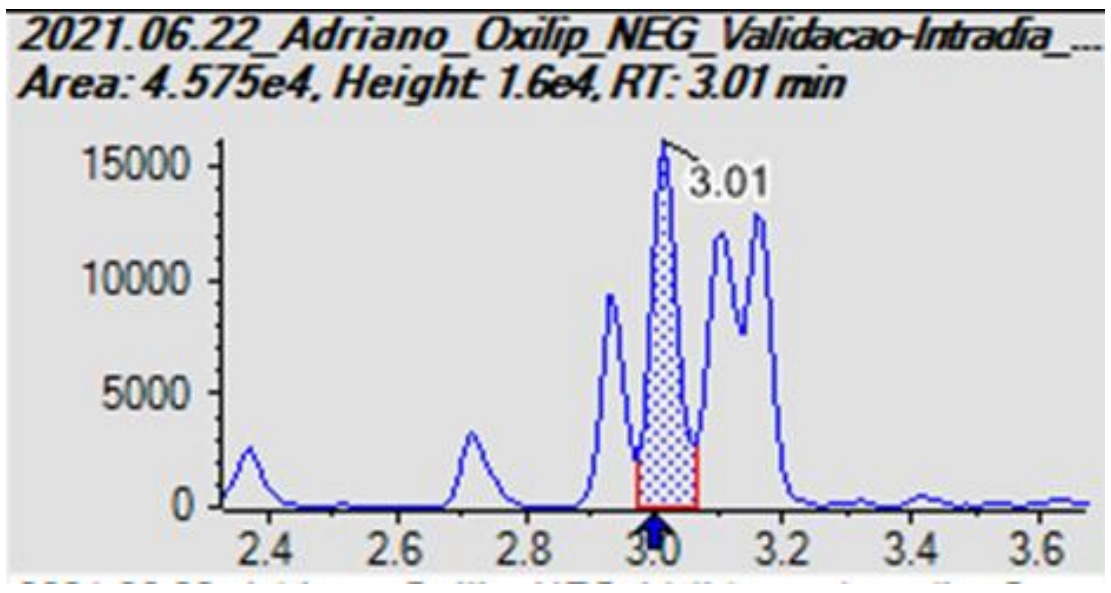

Prostaglandin D2

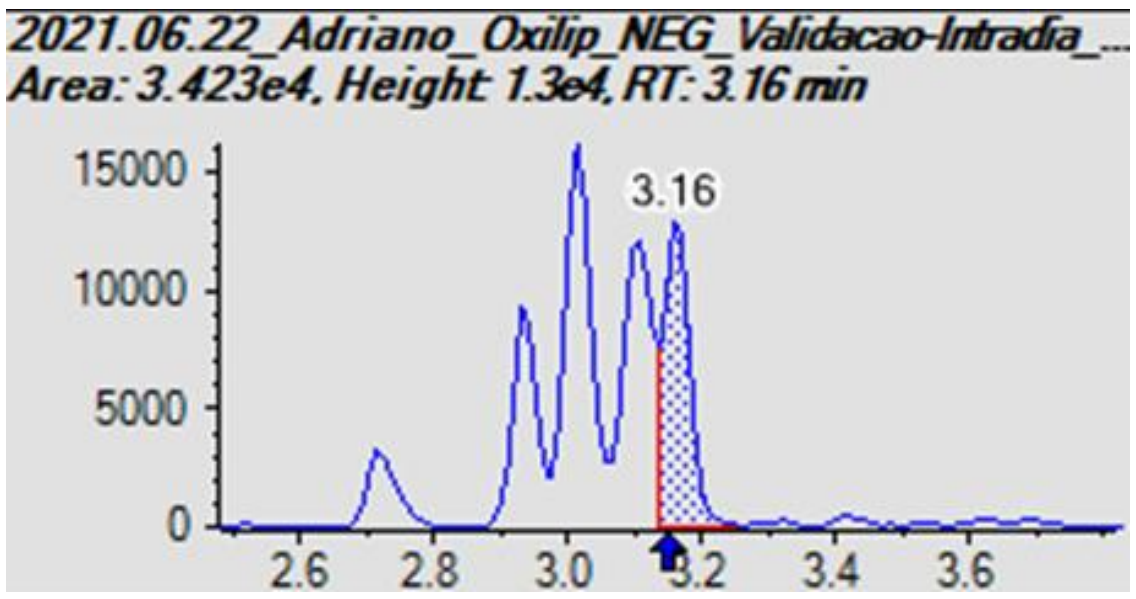

8-iso-PGF2α

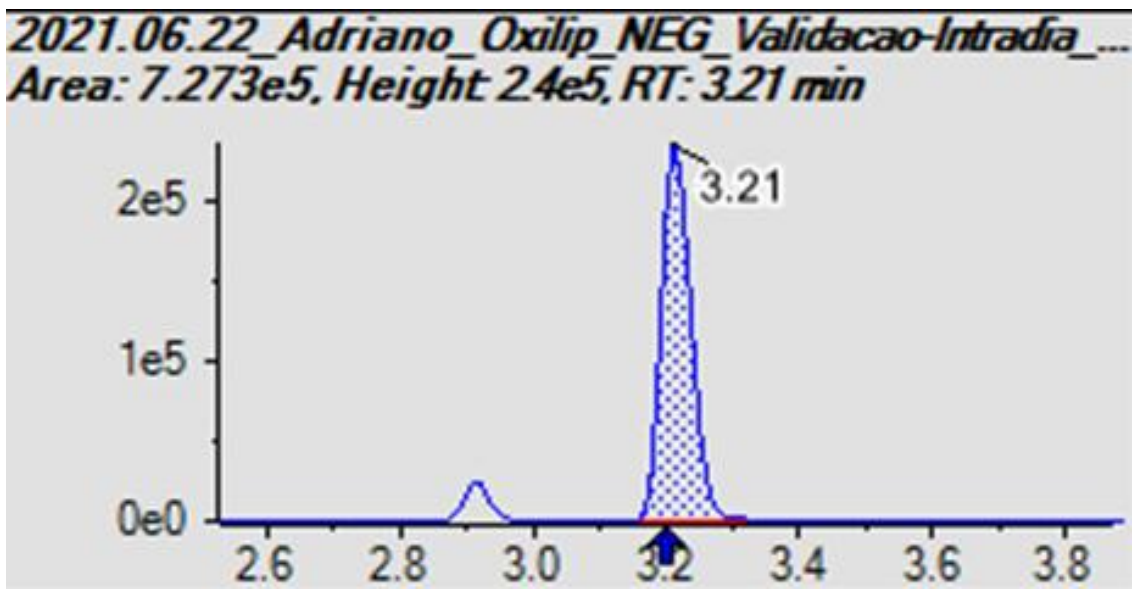

11β-13,14-dihydro-15-keto Prostaglandin F2α

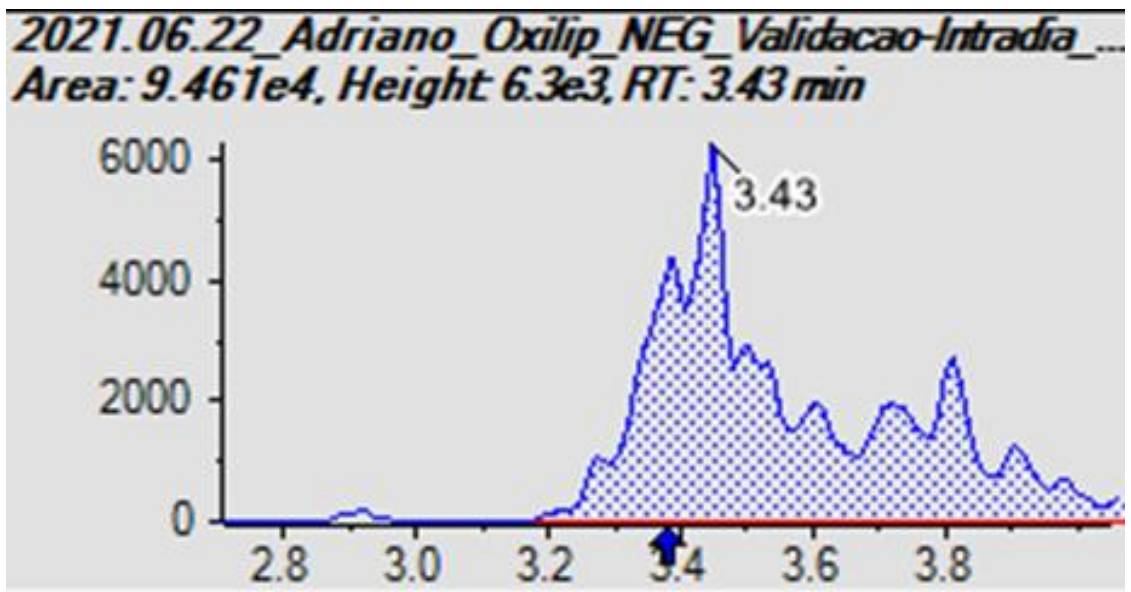

15-keto Prostaglandin F1α

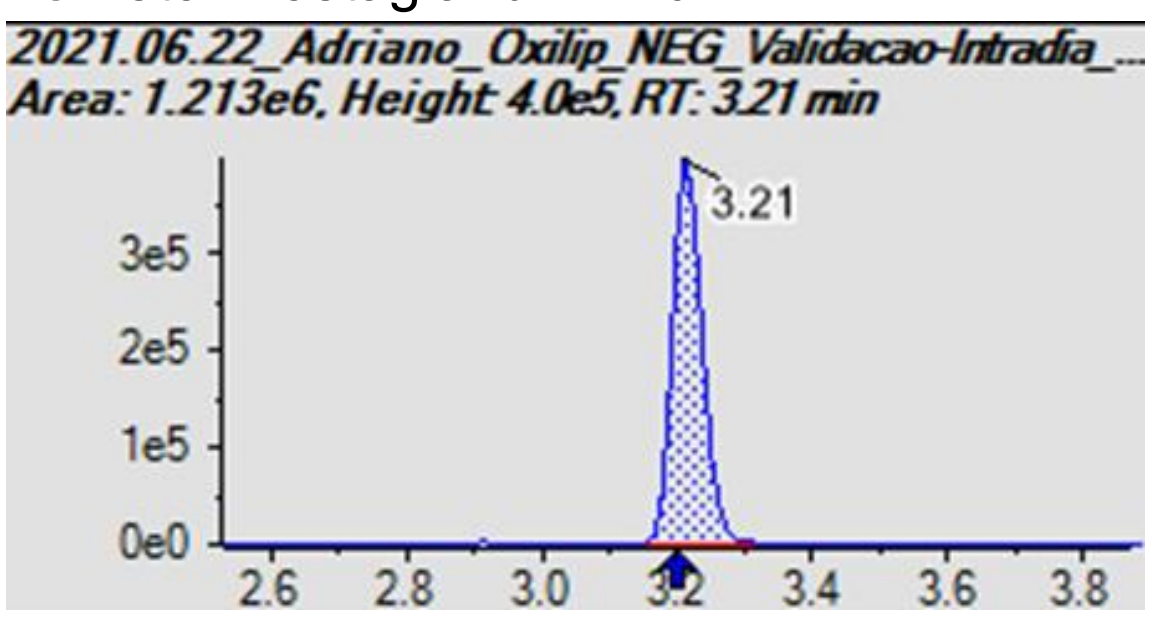

8-iso Prostaglandin E1

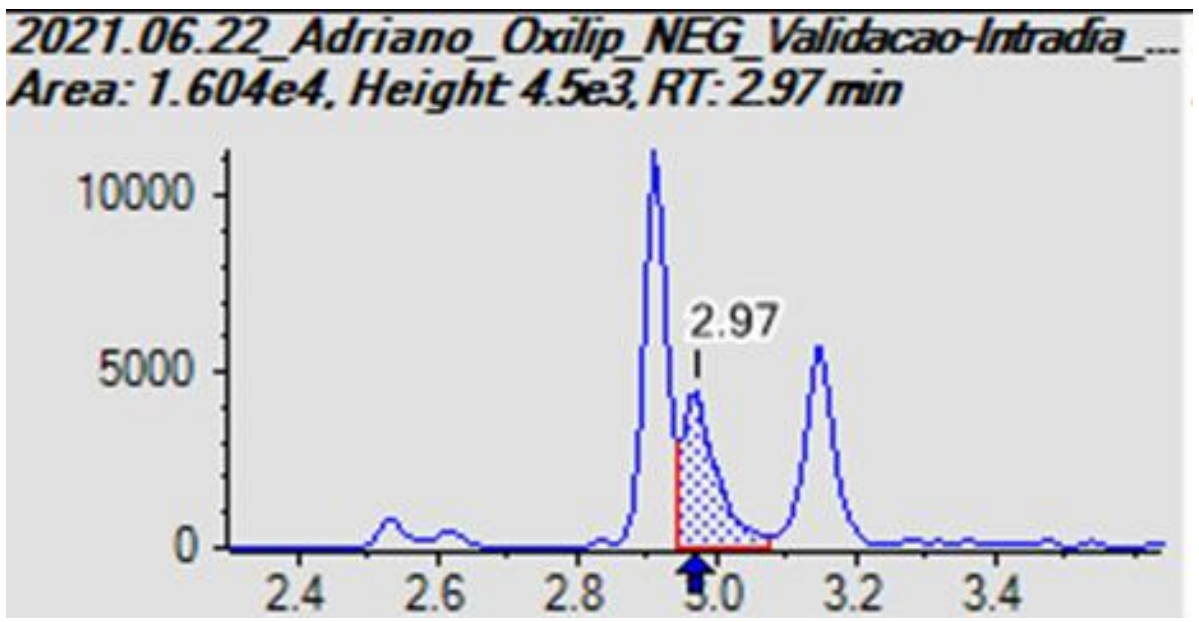

Prostaglandin D1

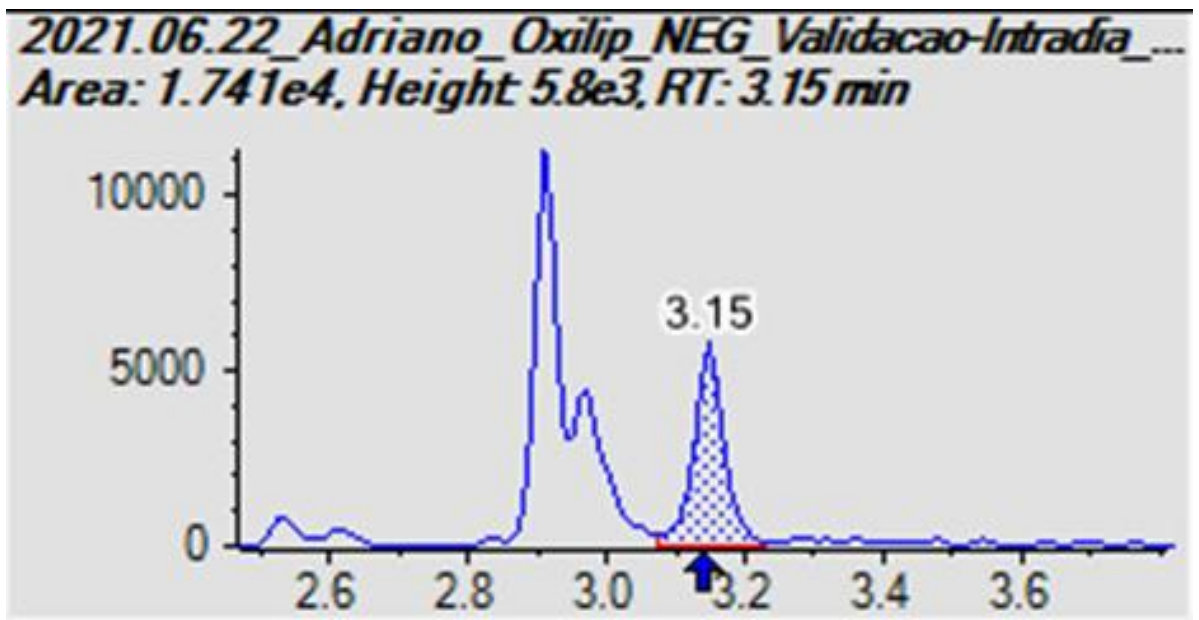

11β-Prostaglandin F2α

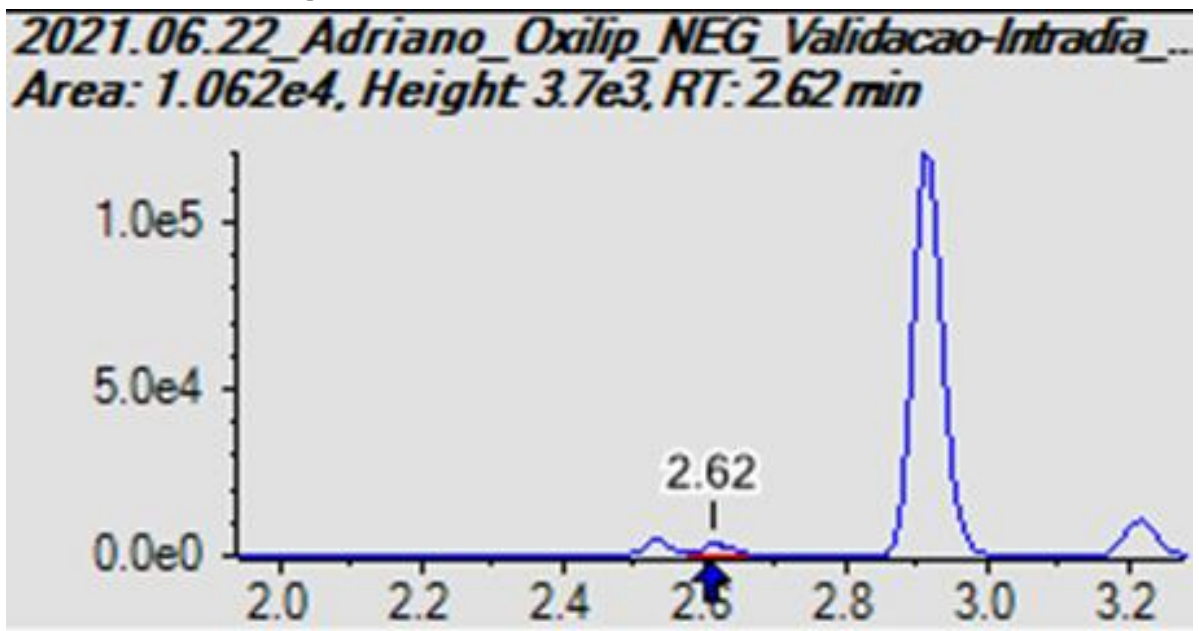

Prostaglandin F2α

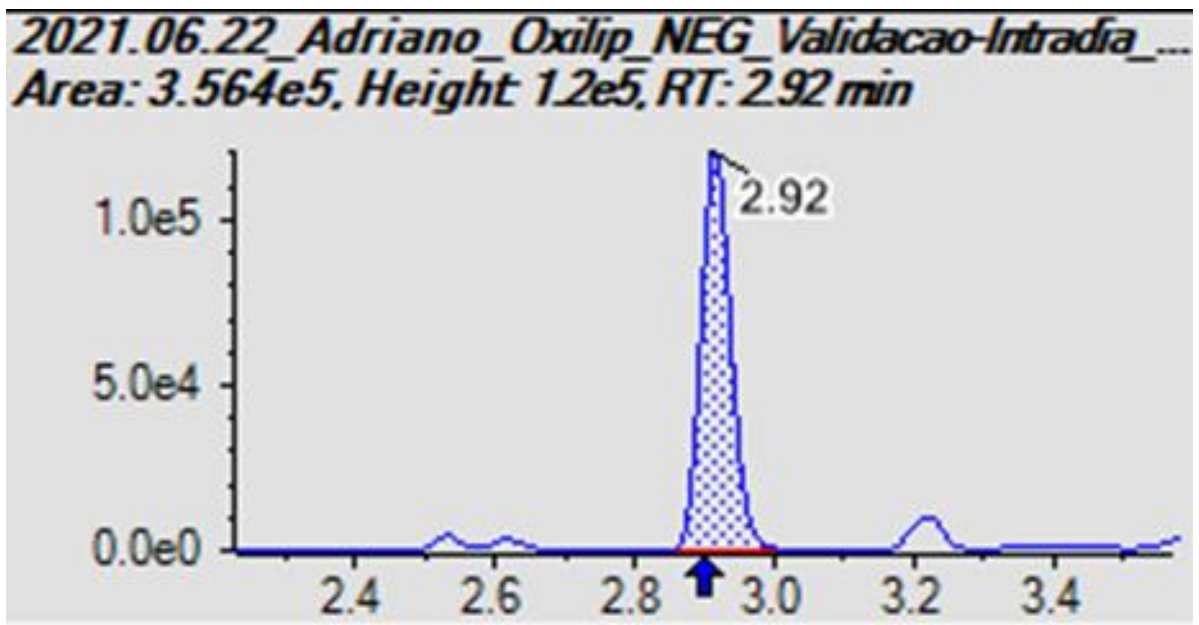

8,12-iso-iPF2α-VI

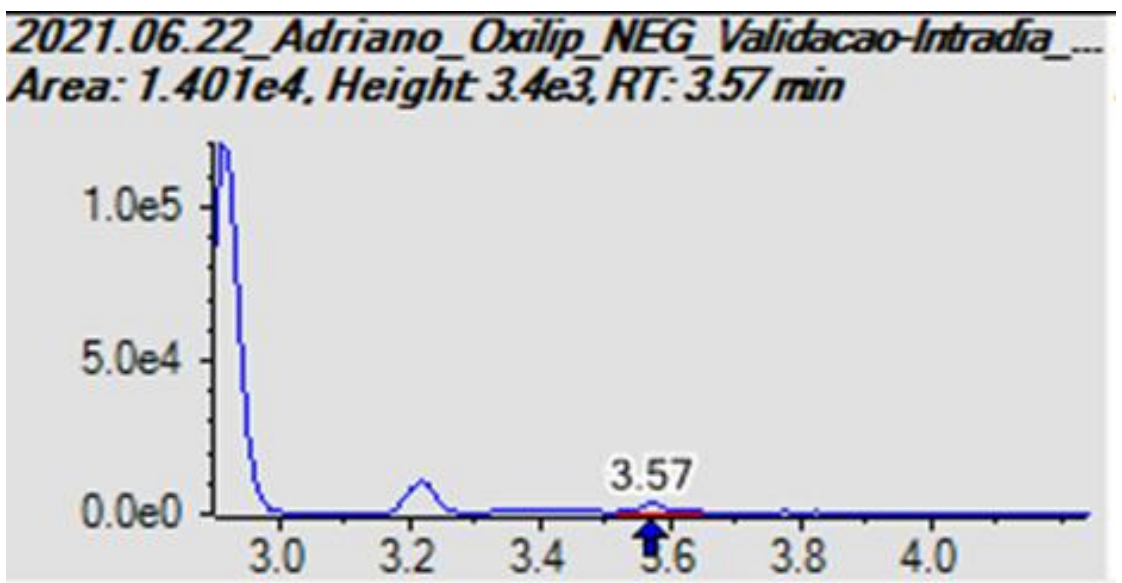

13,14-dihydro Prostaglandin F2α

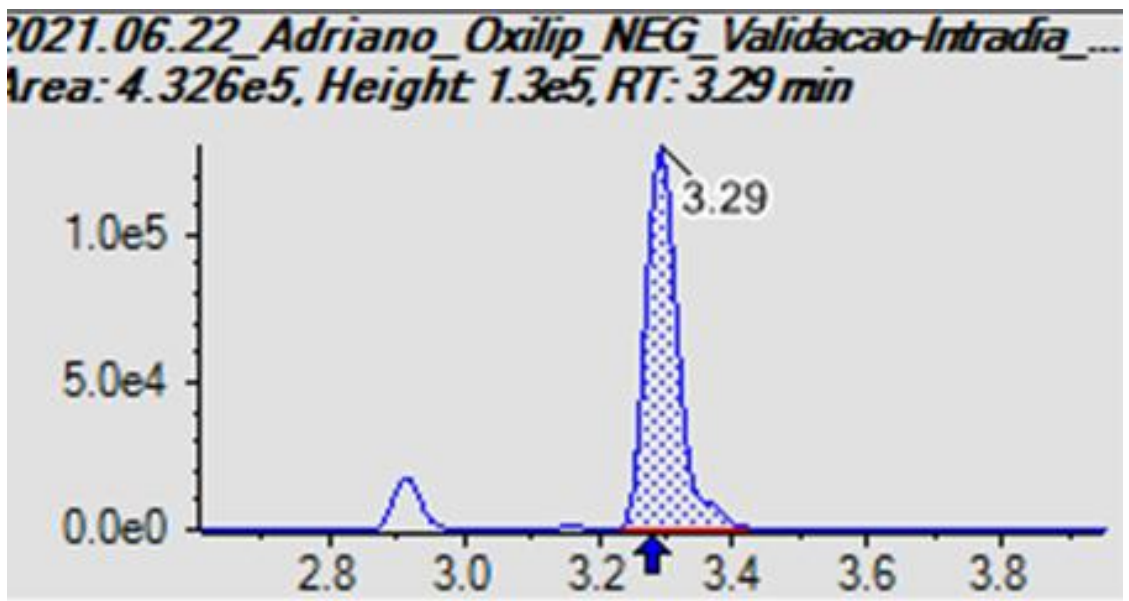

Prostaglandin F1α

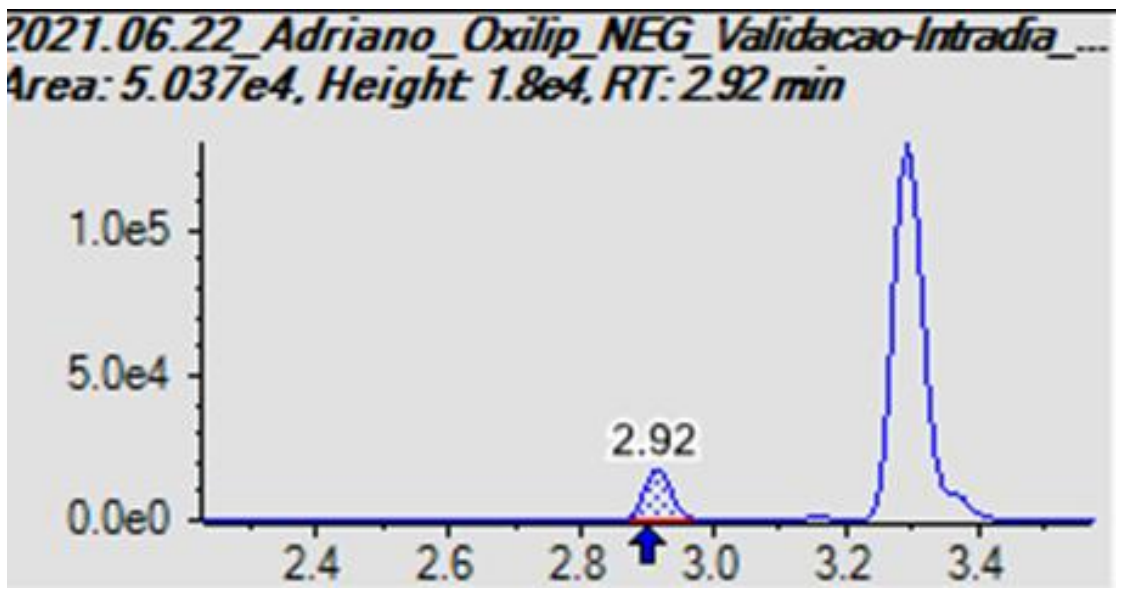

10(S),17(S)-DiHDHA

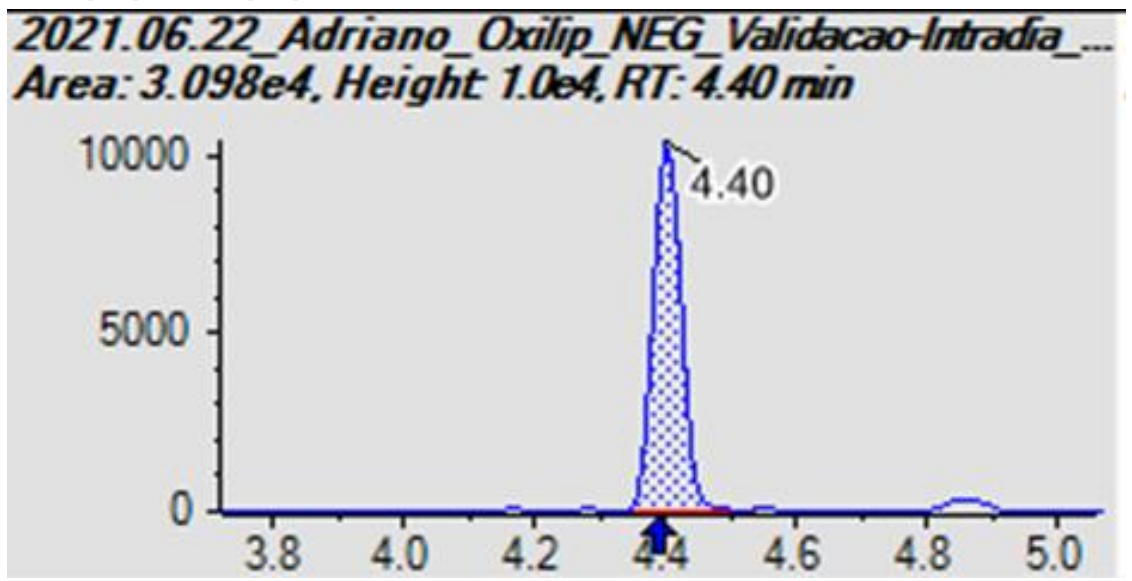

Resolvin D5

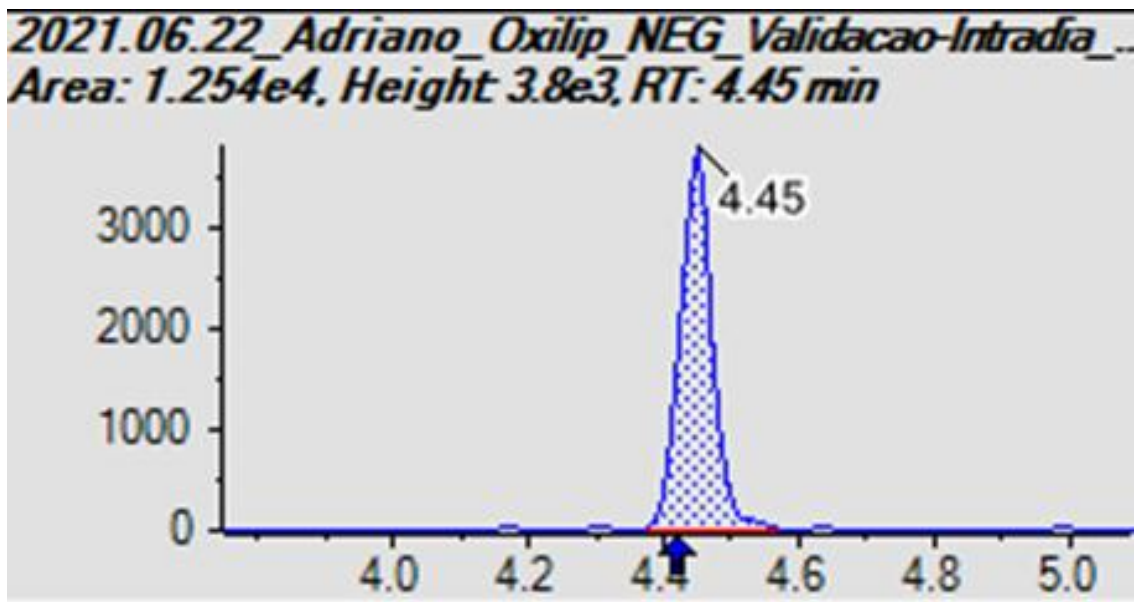

Maresin 2

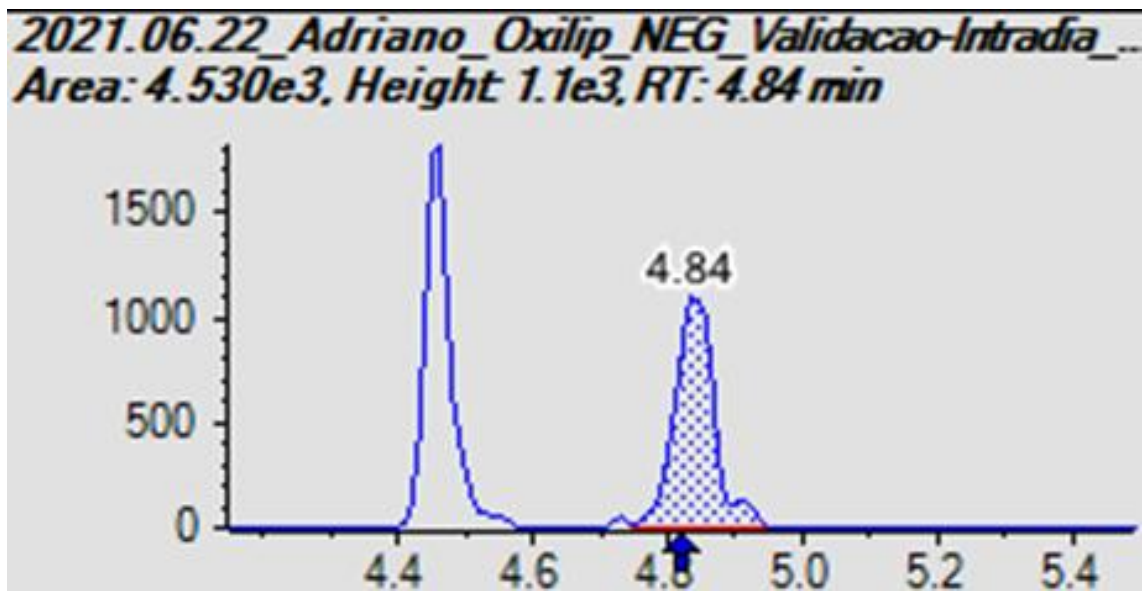

Maresin 1

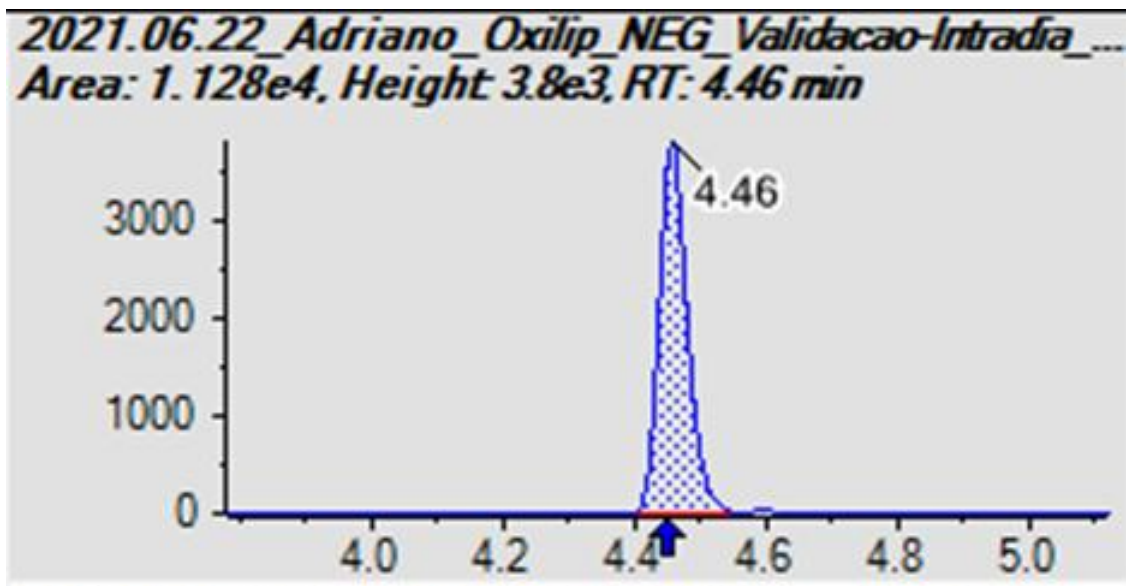

(±)19(20)-DiHDPA

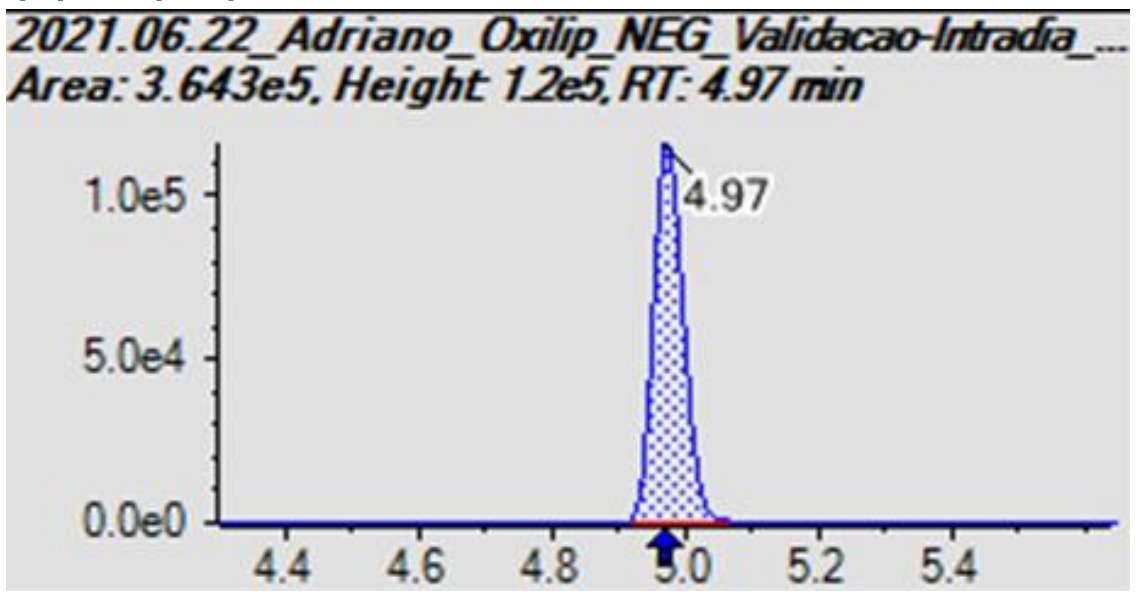

20-carboxy Leukotriene B4

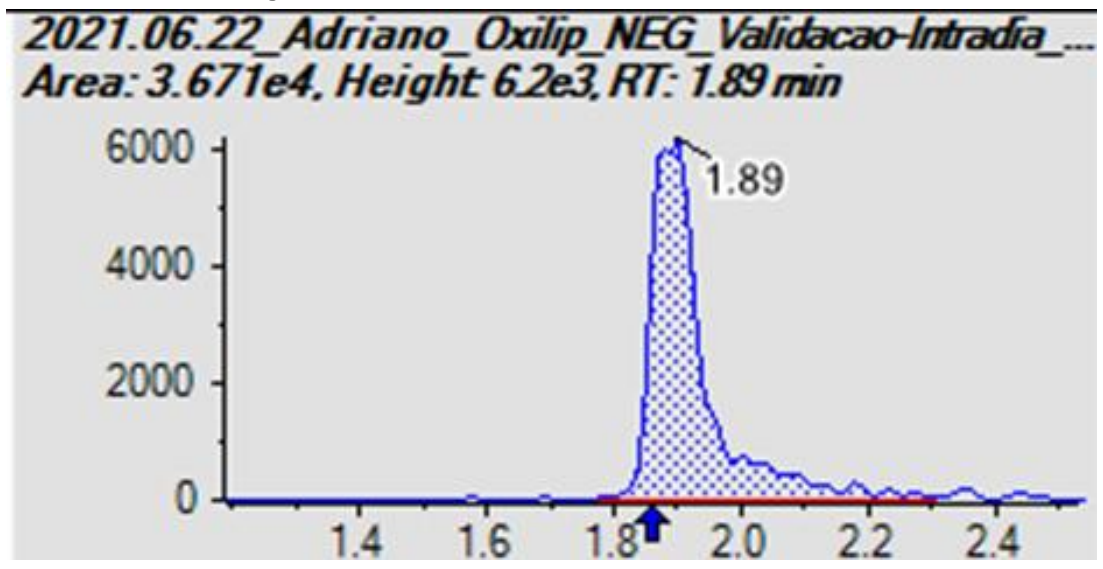

Thromboxane B3

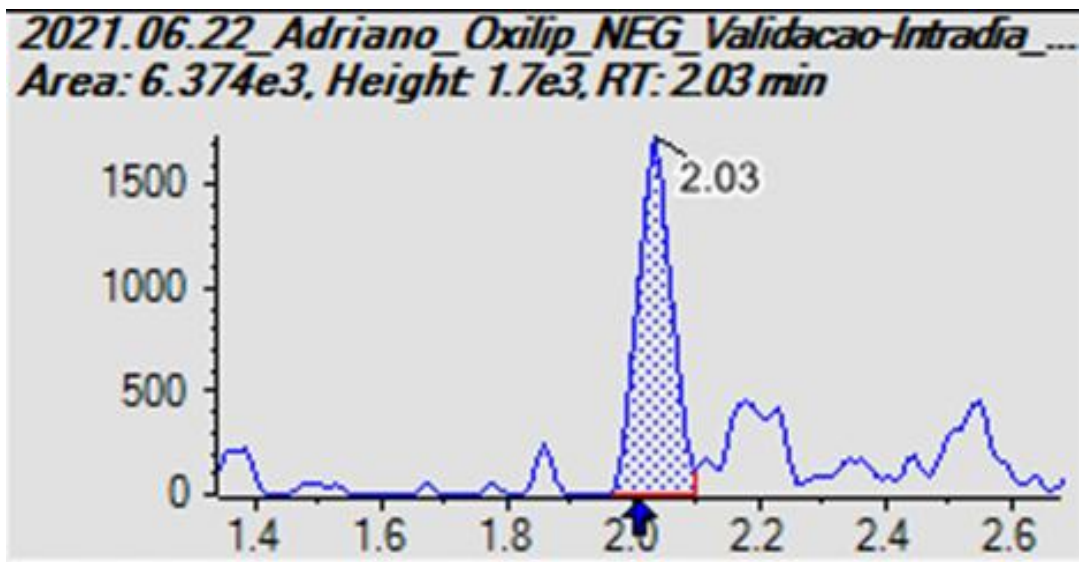

20-hydroxy Prostaglandin E2

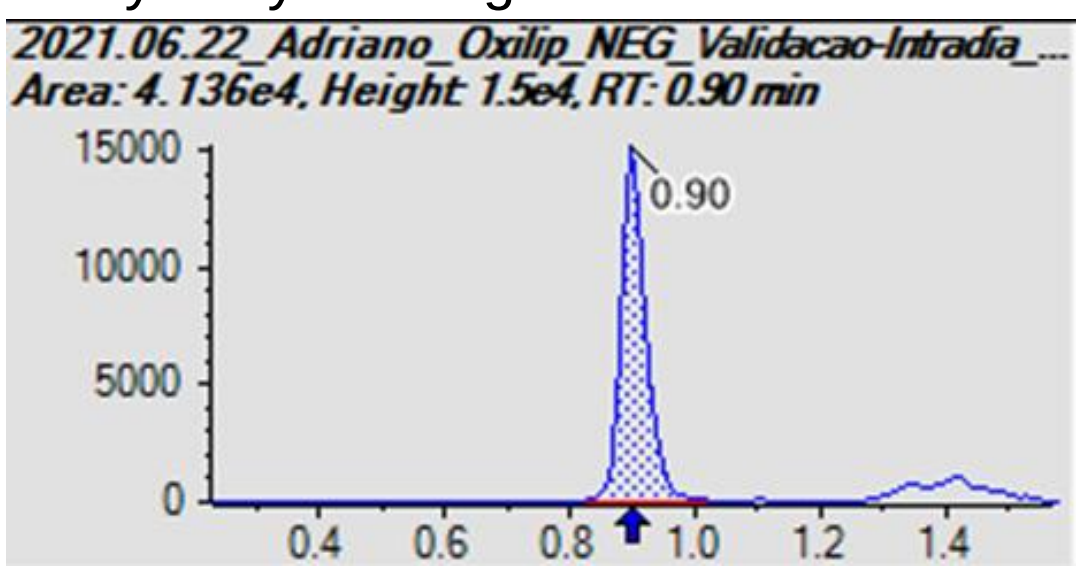

6-keto Prostaglandin E1

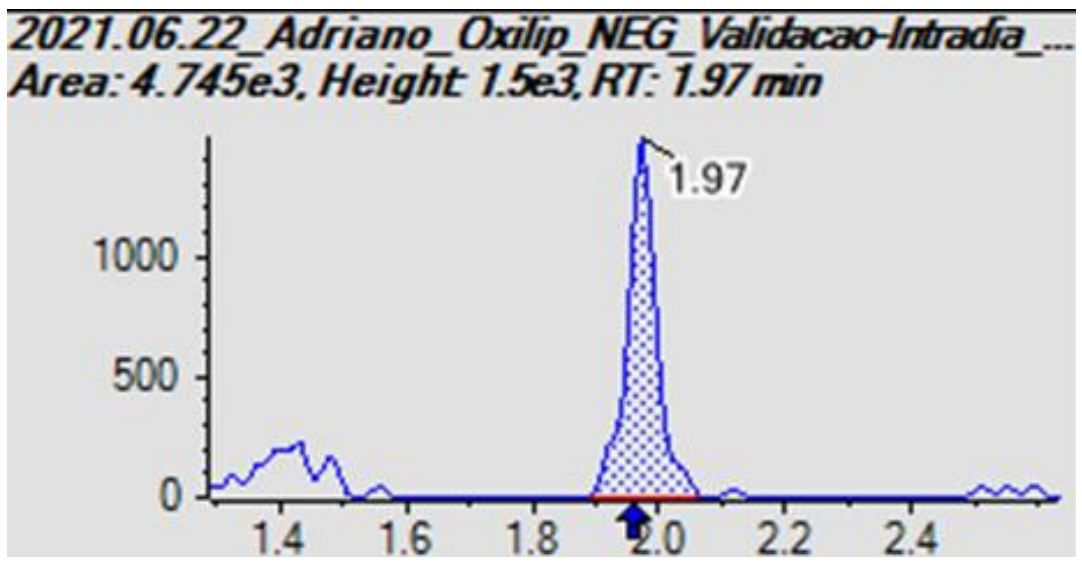

Δ17-6-keto Prostaglandin F1α

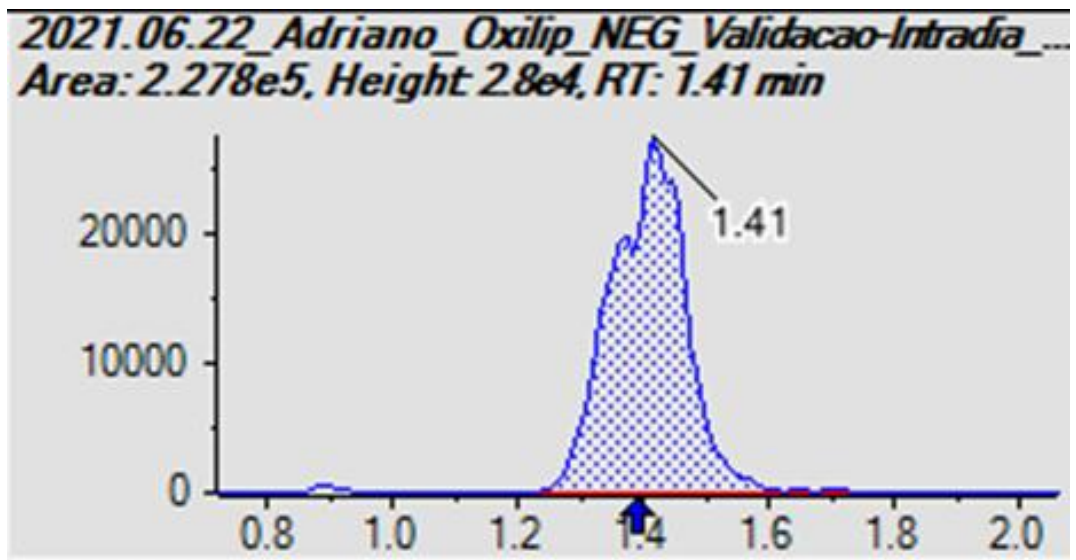

Thromboxane B2

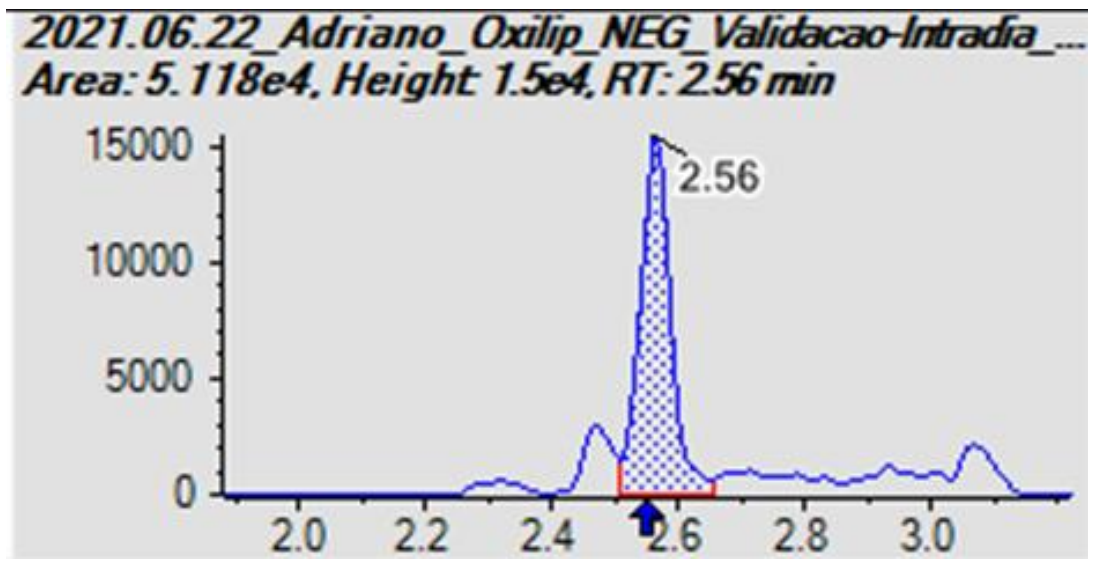

6-keto Prostaglandin F1α

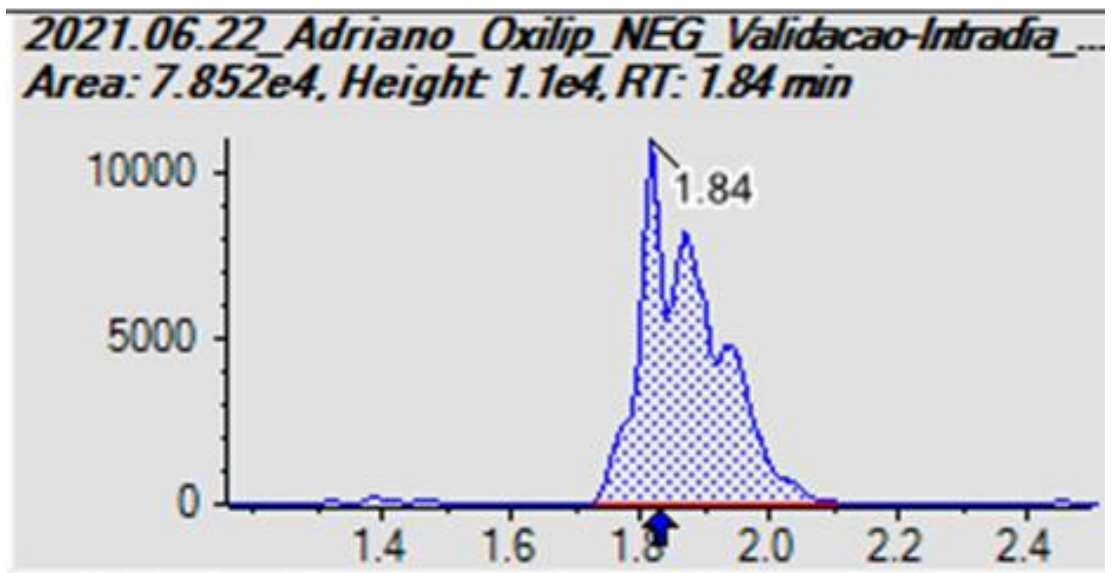

6,15-diketo-13,14-dihydro Prostaglandin F1α

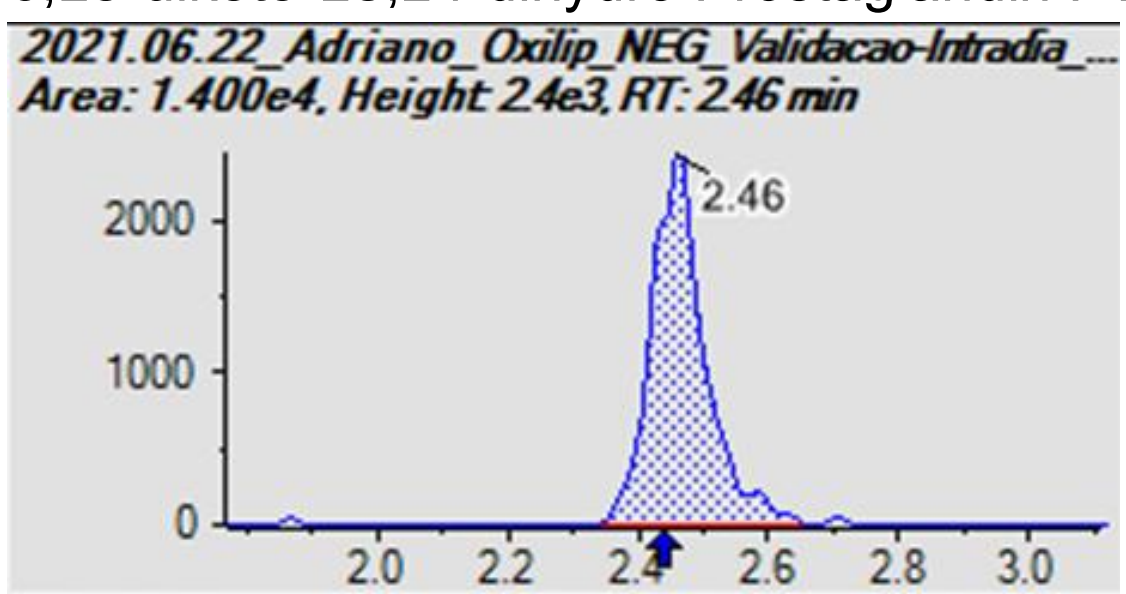

Thromboxane B1

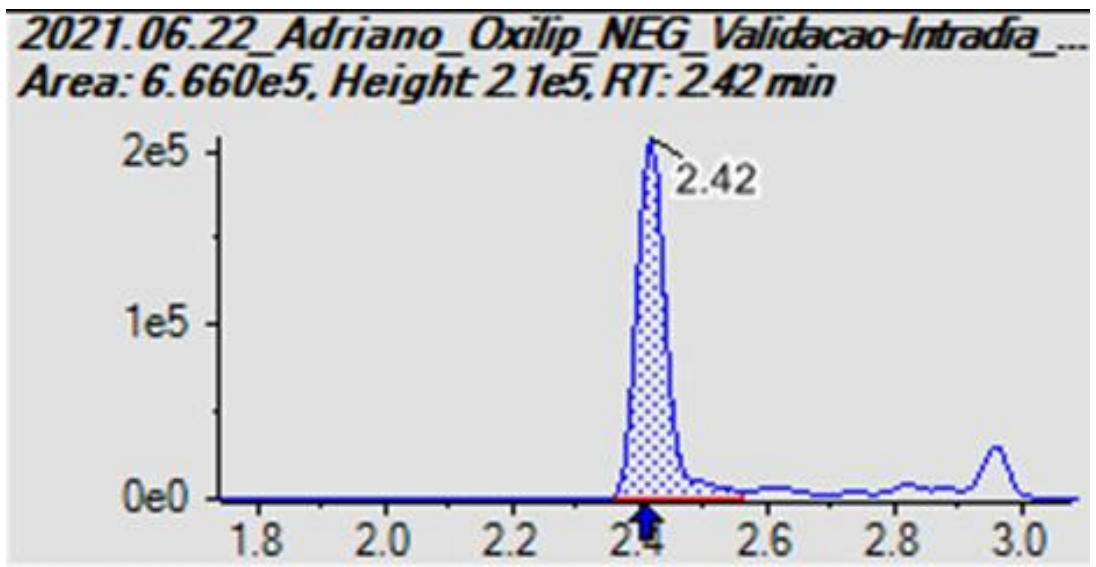

Resolvin D1

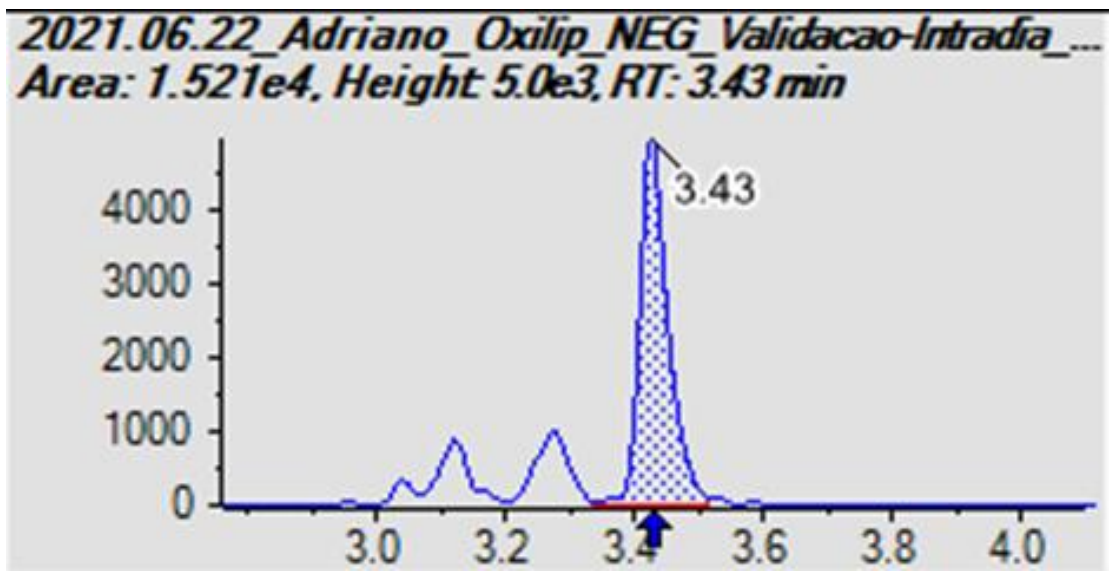

Resolvin D3

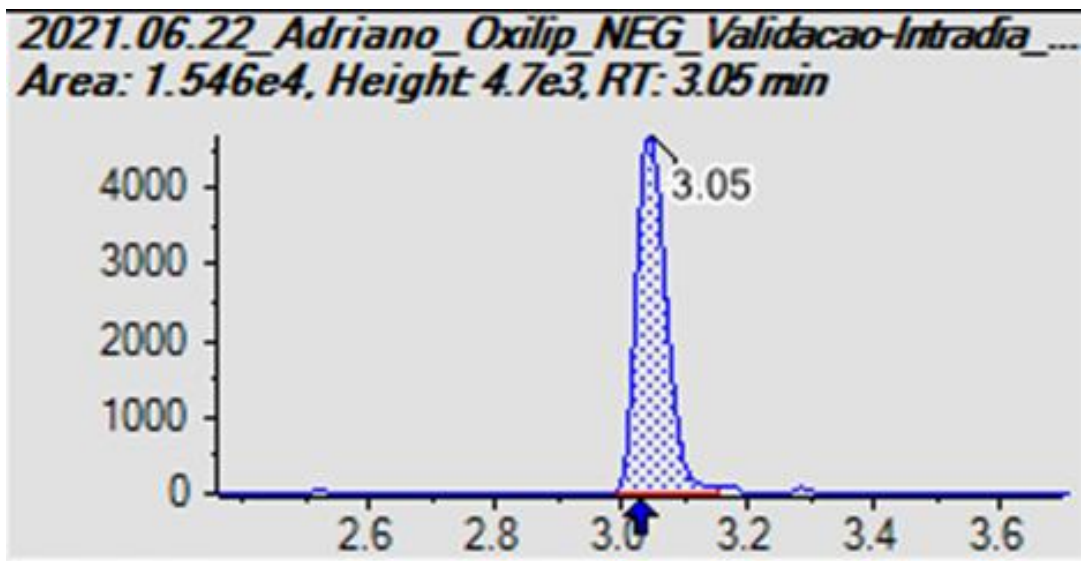

16,16-dimethyl Prostaglandin D2

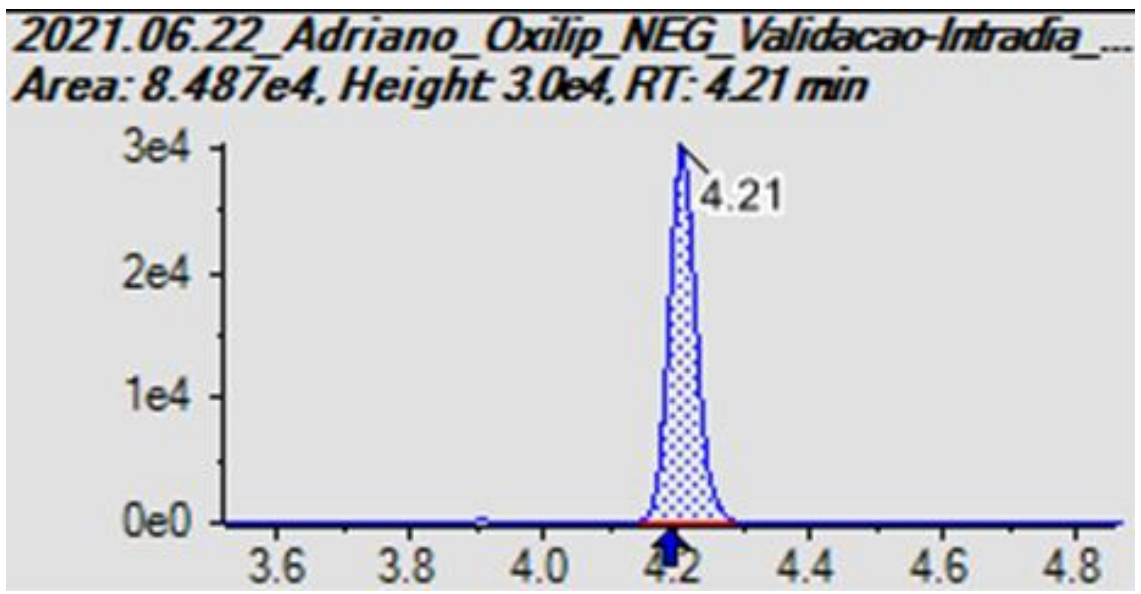

Resolvin D2

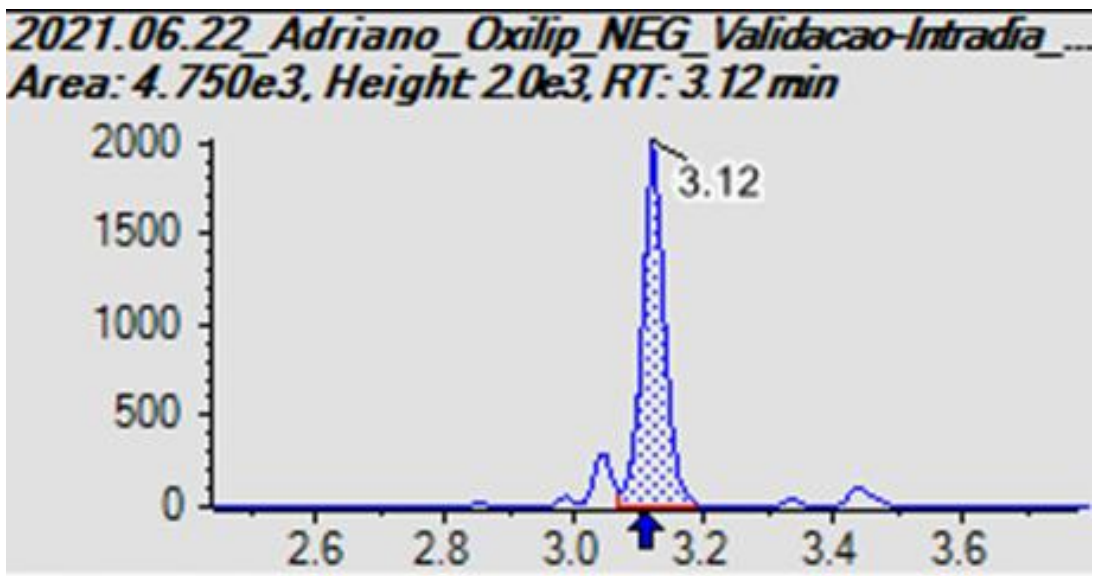

1a,1b-dihomo Prostaglandin E2

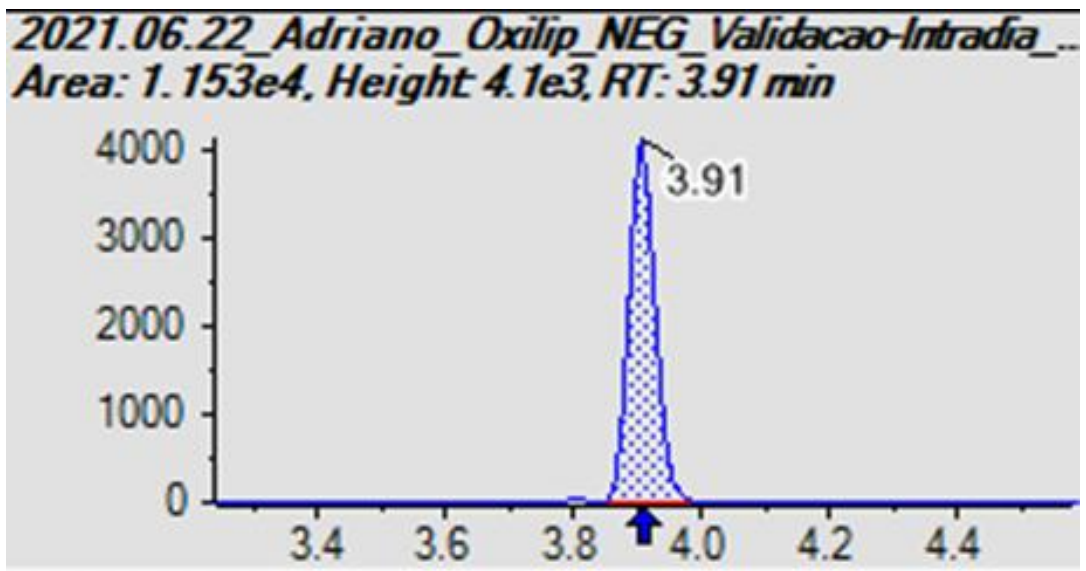

13-HODE-d4

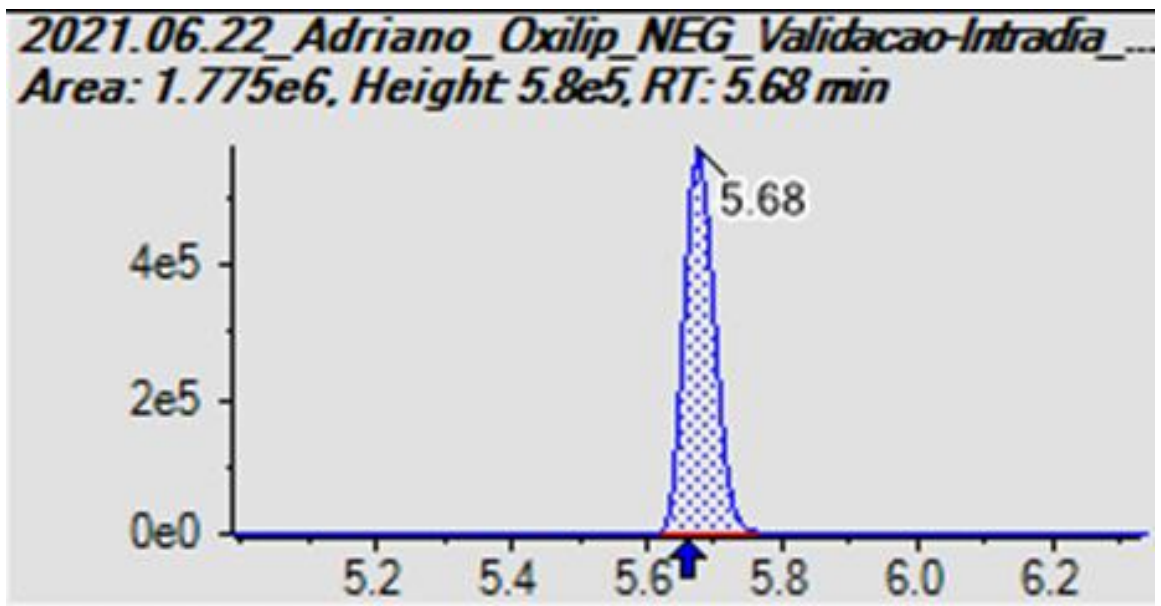

8-iso-PGF2a-d4

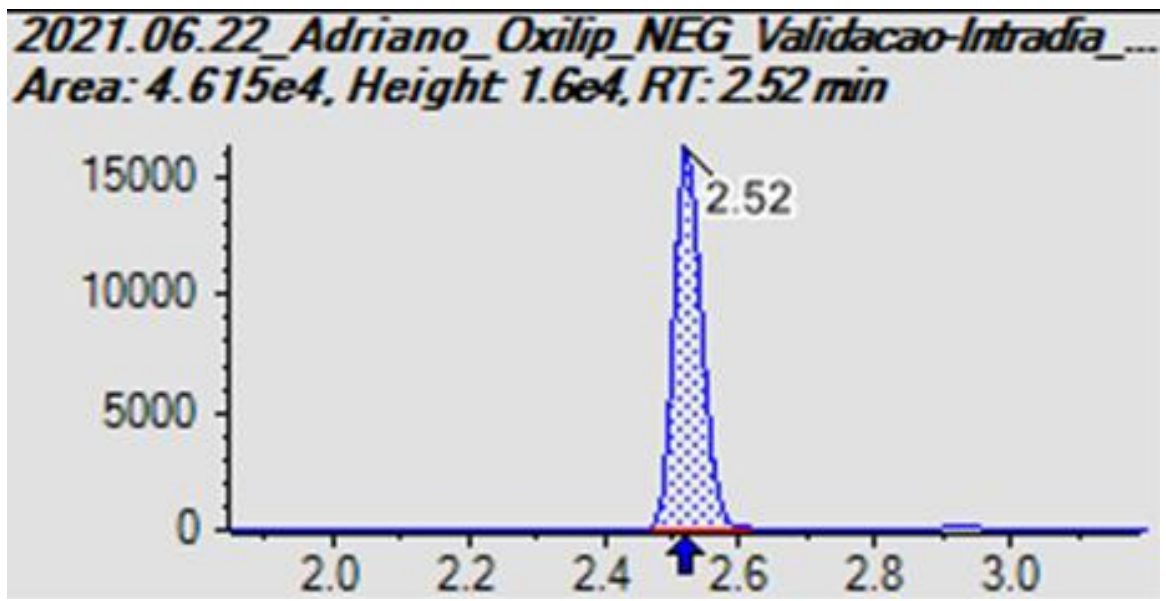

12(13)-DiHOME-d4

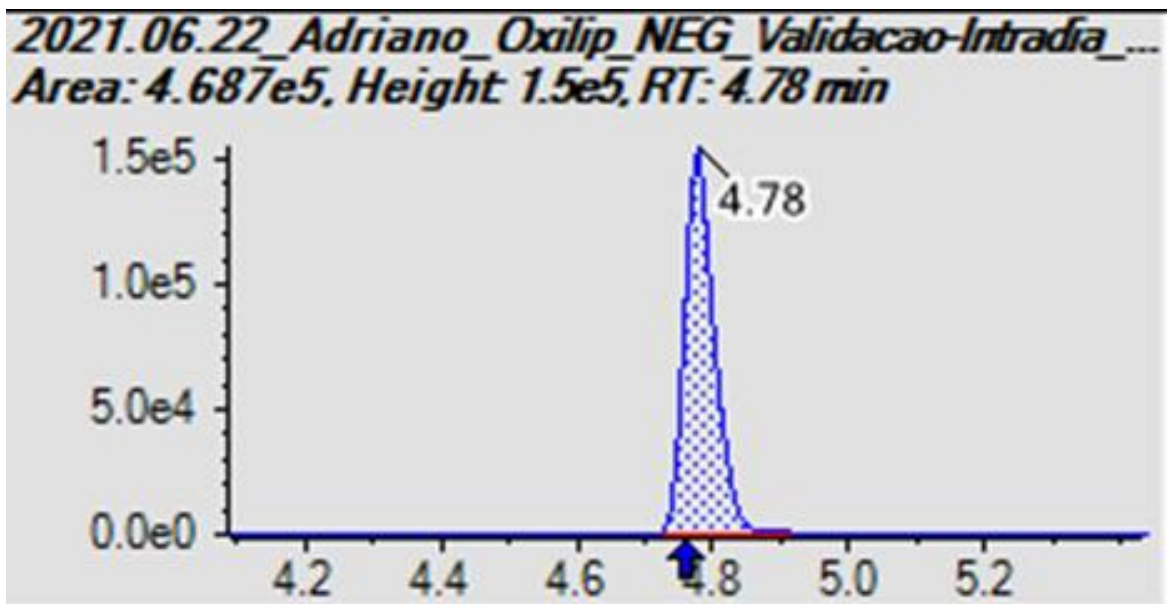

11(12)-EET-d11

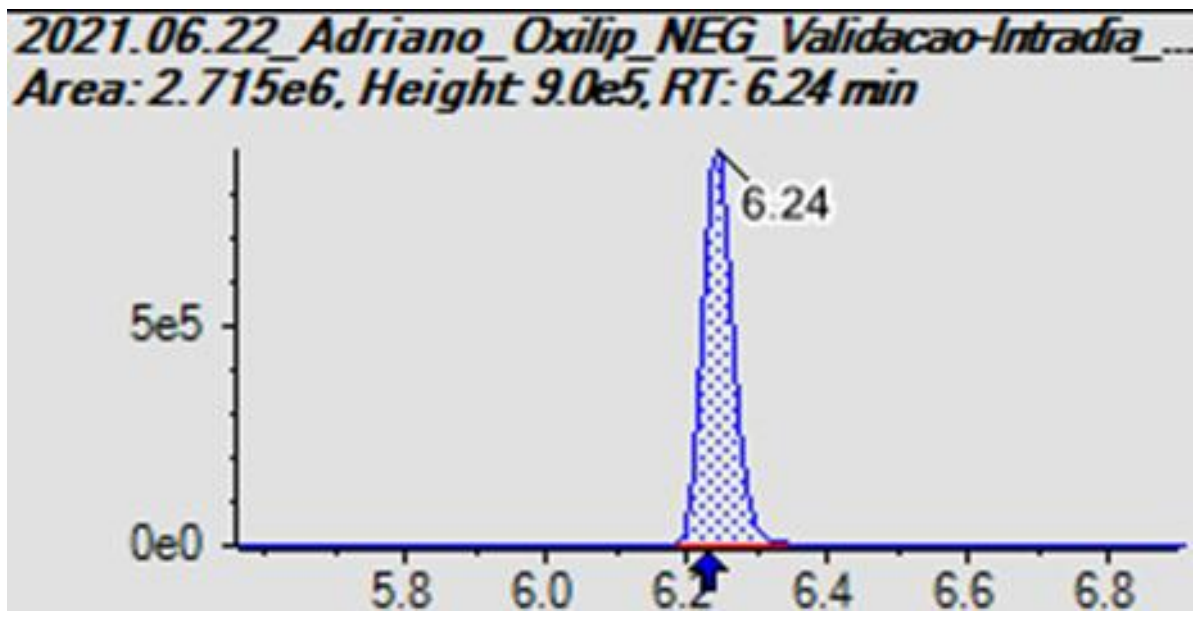

15-deoxy-Δ12,14-Prostaglandin J2-d4

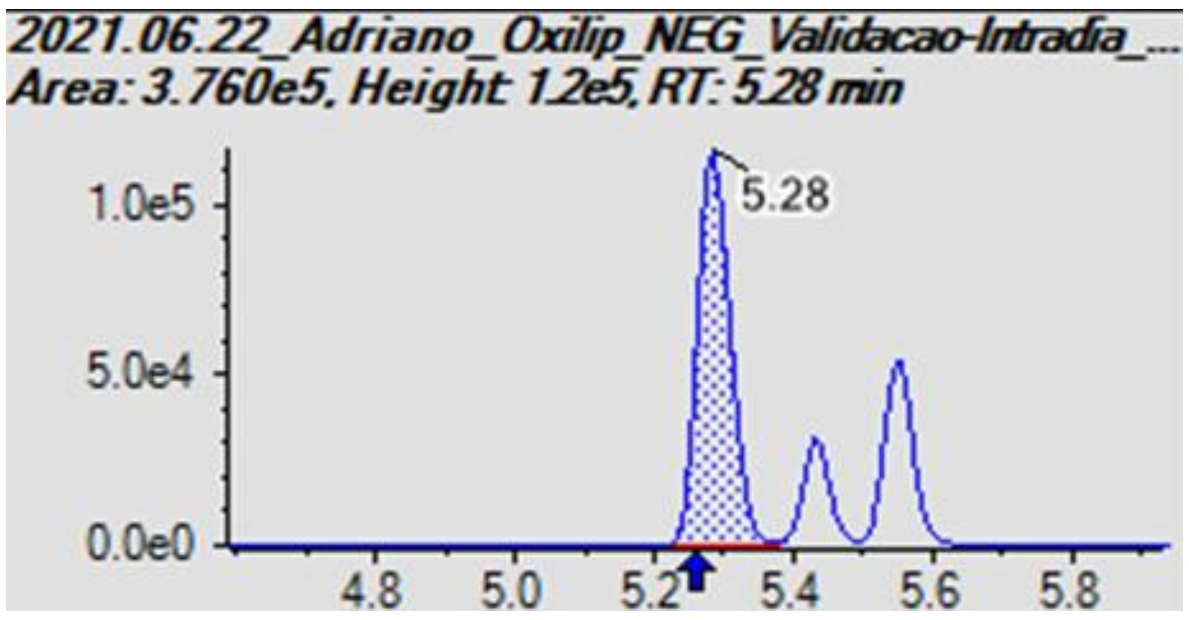

Prostaglandin A2-d4

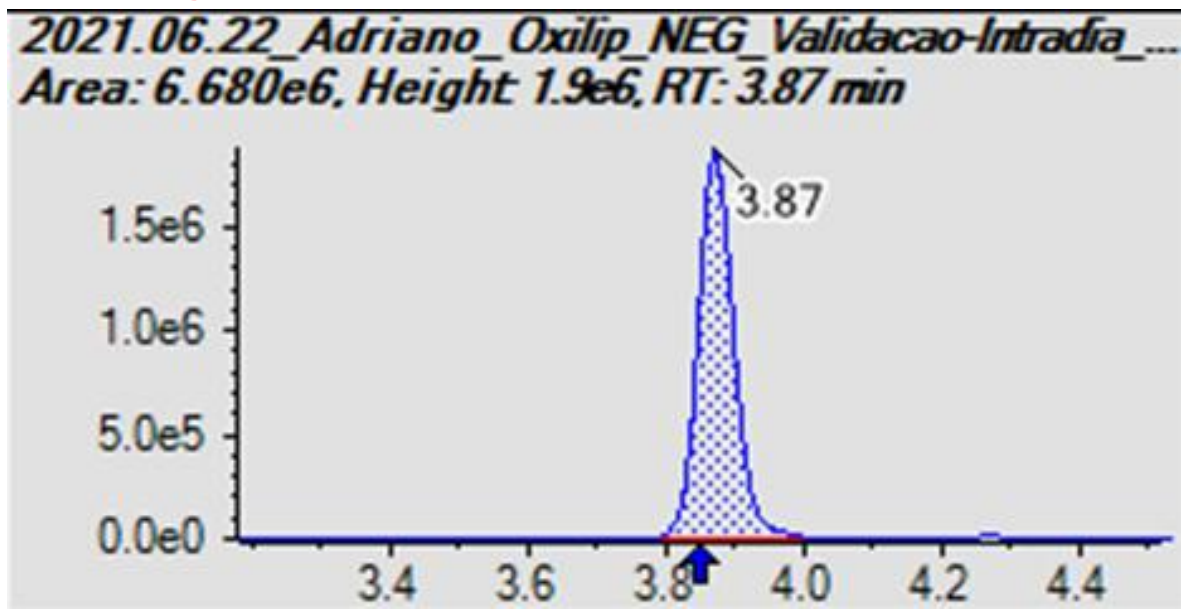

5-oxo-ETE-d7

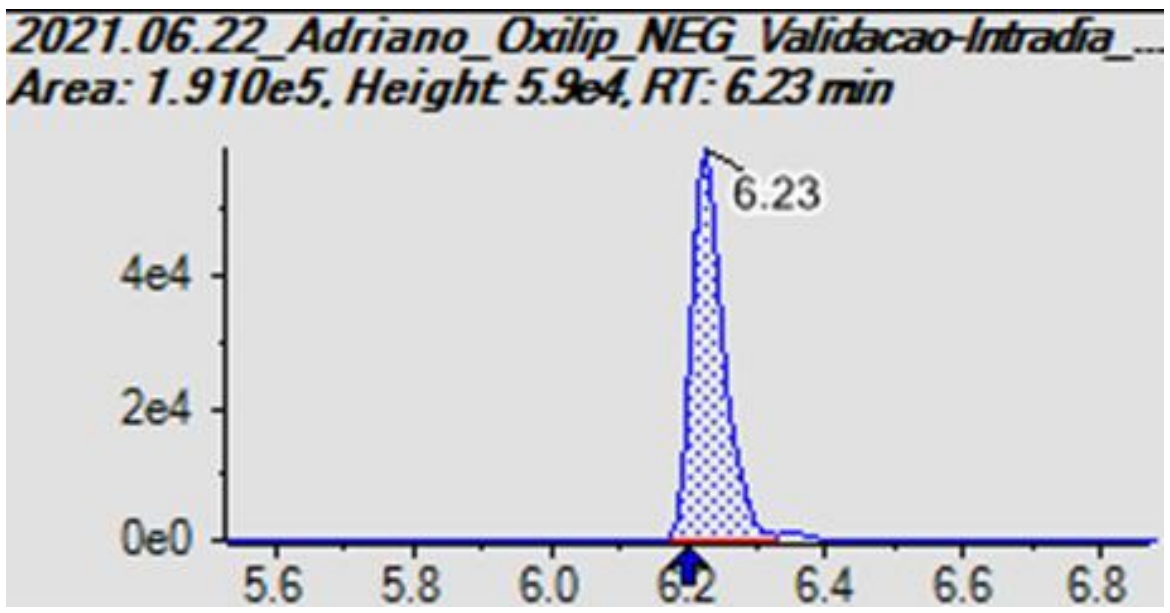

9-Nitrooleate-d17

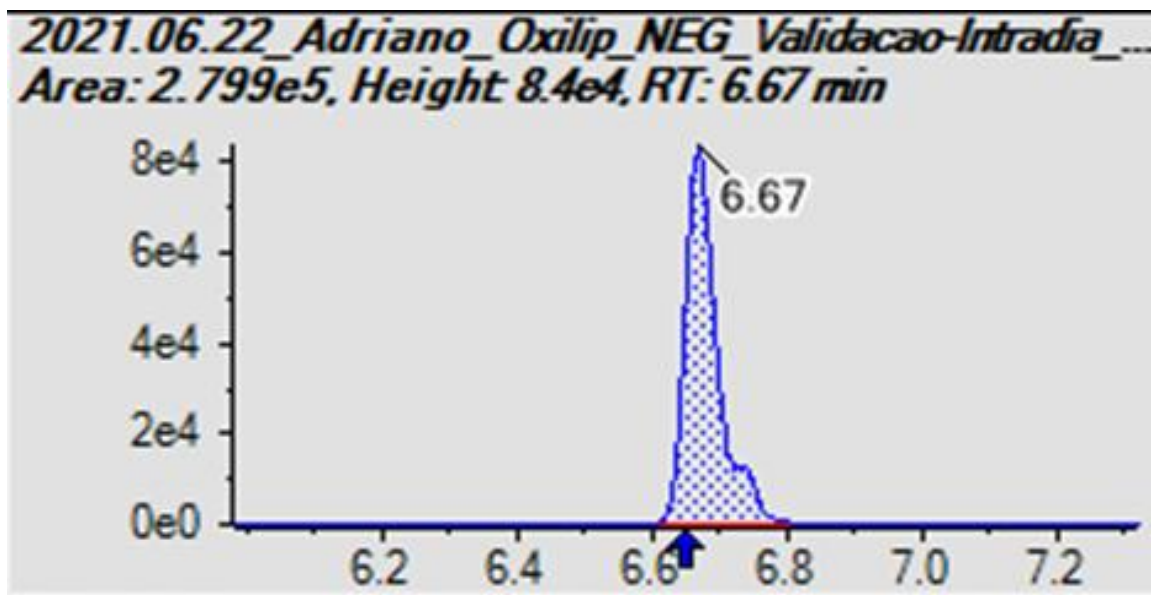

5-HETE-d8

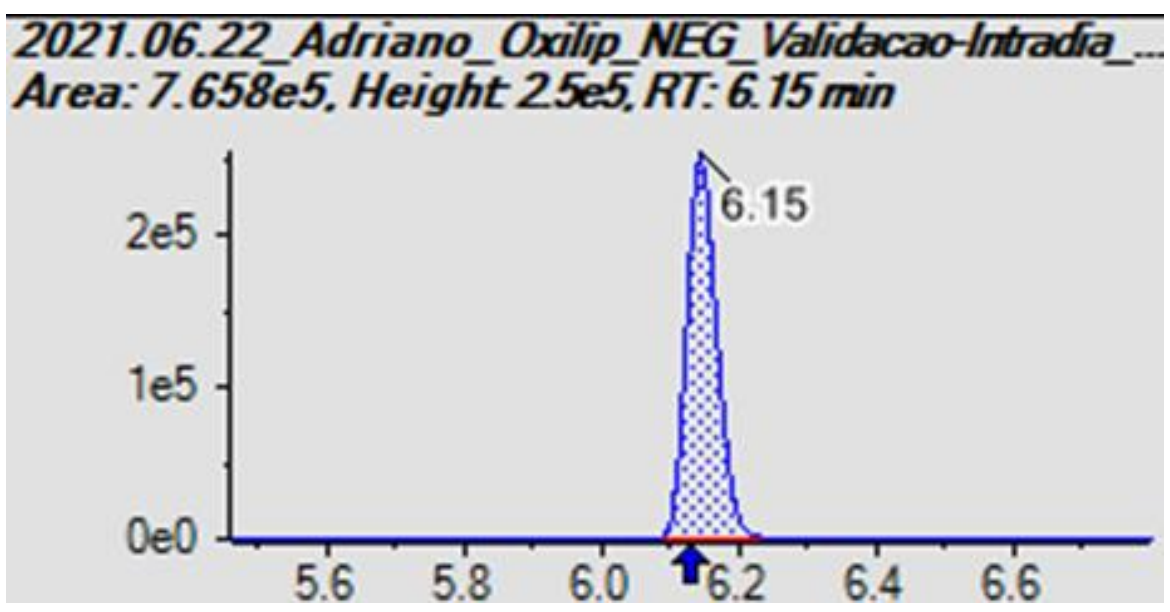

Prostaglandin E2-d4

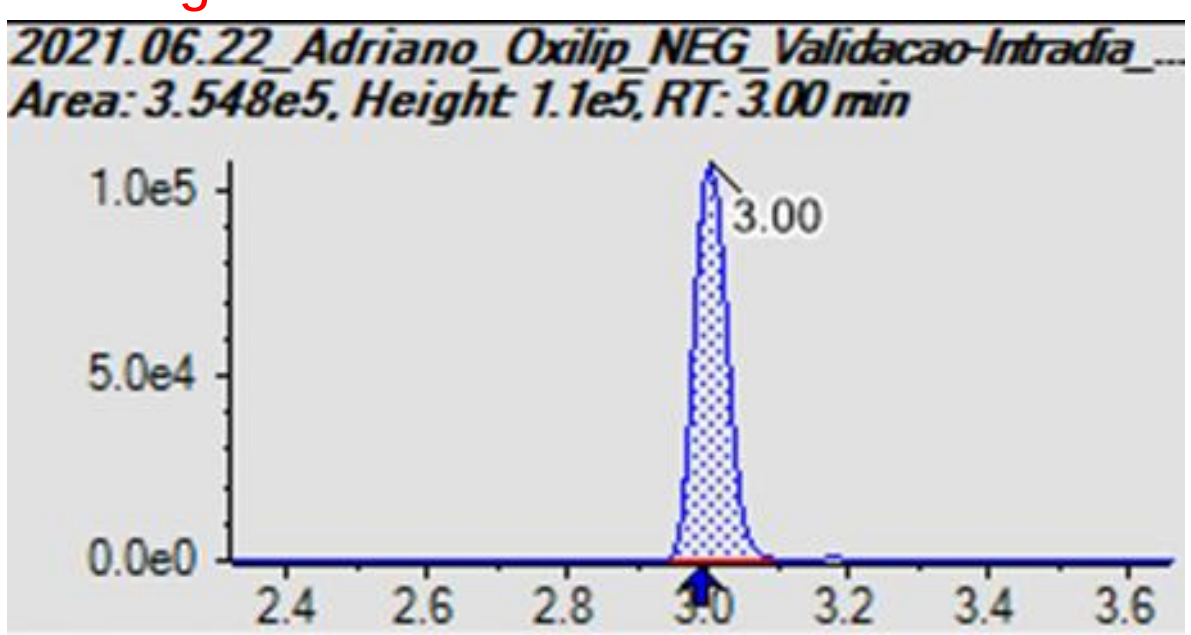

Lipoxin A4-d5

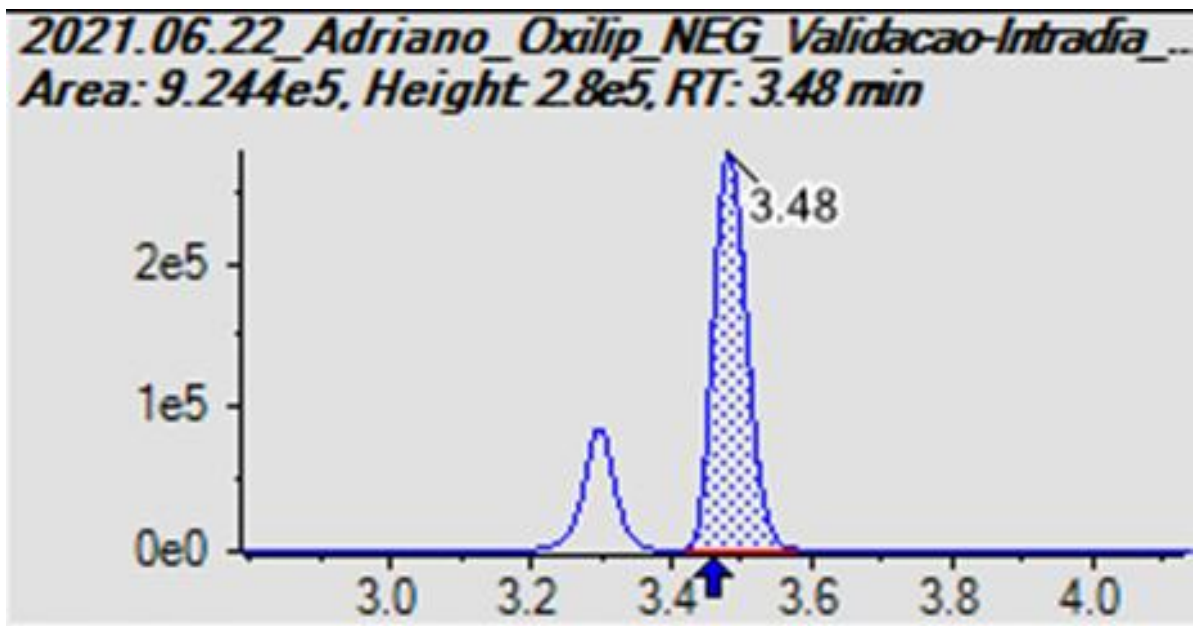

Resolvin D1-d5

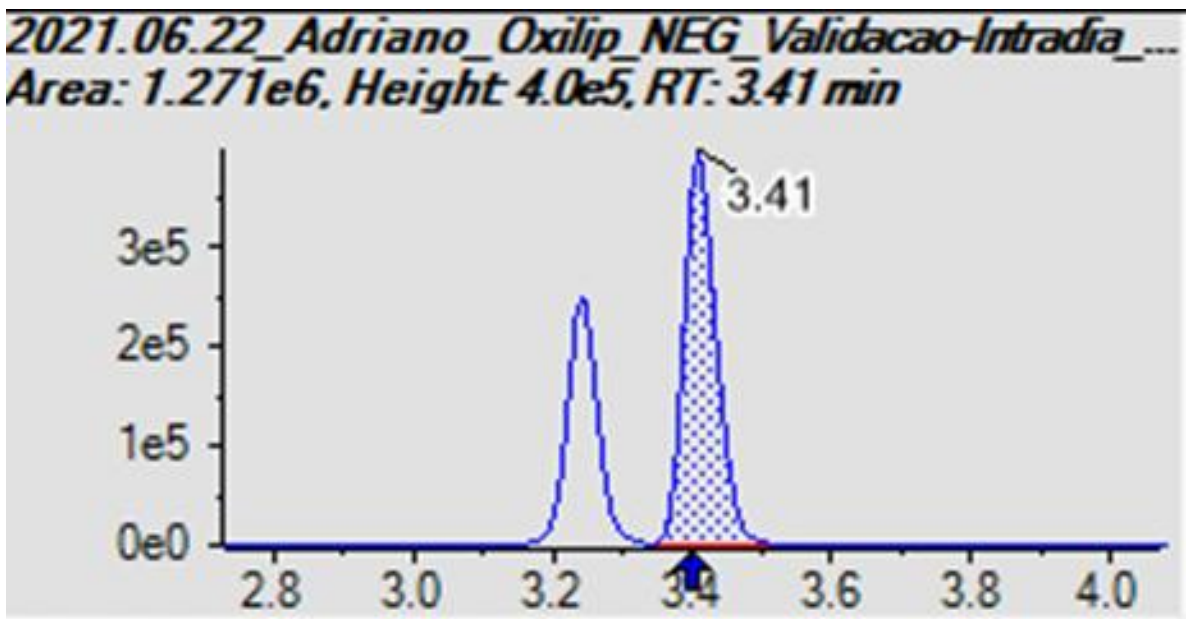

Thromboxane B2-d4

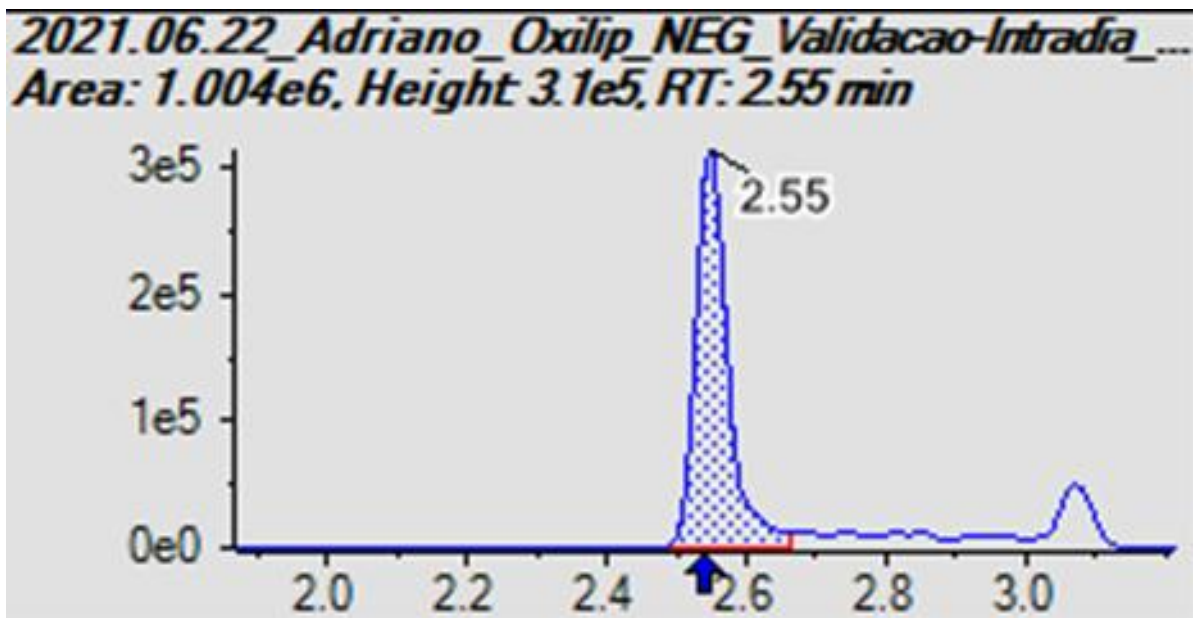

Figure S1. Comparative analysis of oxylipins detected in RAW 264.7 macrophages, related to Expected Outcomes.

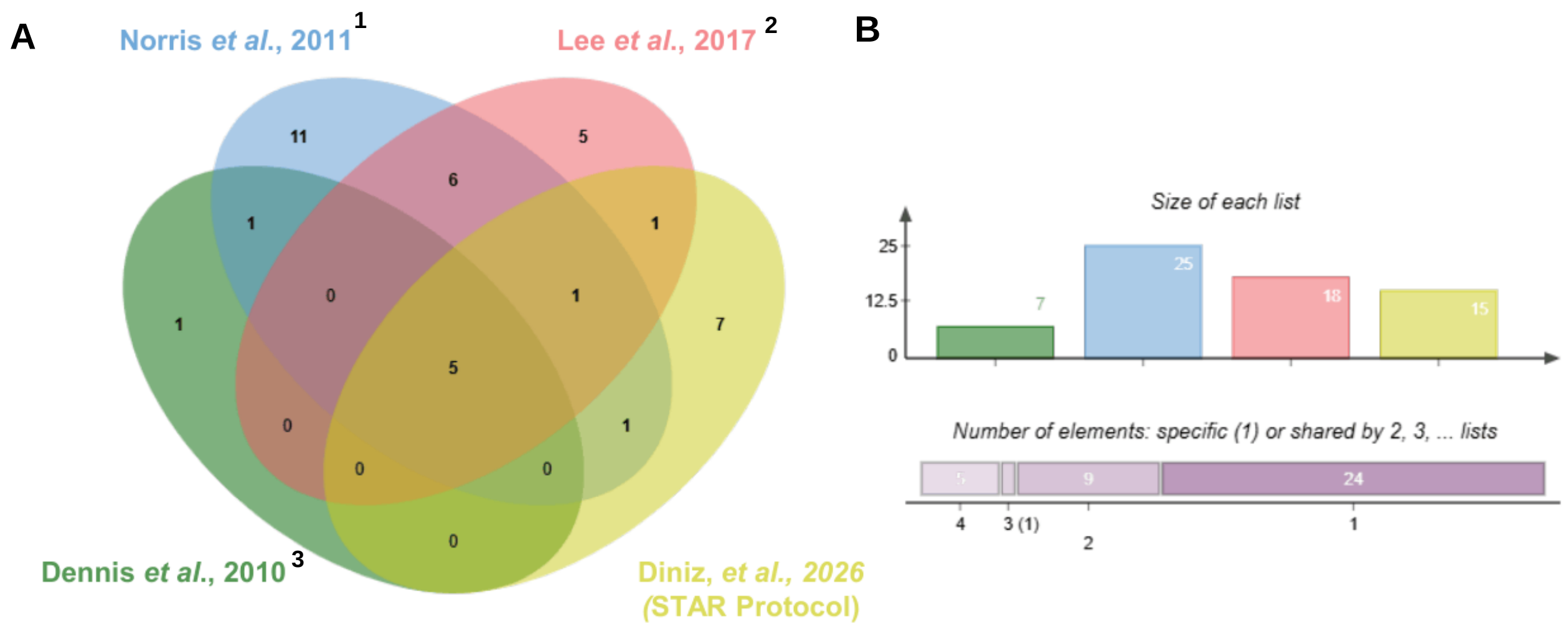

A) Venn diagram showing the overlap between oxylipins identified in the present study (yellow set, n = 15; including both cell pellet and supernatant samples) and those previously reported in the literature for RAW 264.7 macrophages under LPS or KLA stimulation. Of the 15 detected oxylipins, 8 overlapped with previously reported metabolites, including 5 oxylipins ($PGF_{2\alpha}$, PGD_2, PGE_2, 15-deoxy-PGD_2, and 11-HETE) that were consistently detected across all four studies. The Venn diagram was generated using *jvenn*[4]. **B)** The bar plots show the amount of oxylipins reported in each article.

1. Norris, P.C., Reichart, D., Dumlao, D.S., Glass, C.K., and Dennis, E.A. (2011). Specificity of eicosanoid production depends on the TLR-4-stimulated macrophage phenotype. J. Leukoc. Biol. *90*, 563–574. https://doi.org/10.1189/jlb.0311153.

2. Dennis, E.A., Deems, R.A., Harkewicz, R., Quehenberger, O., Brown, H.A., Milne, S.B., Myers, D.S., Glass, C.K., Hardiman, G., Reichart, D., et al. (2010). A mouse macrophage lipidome. Journal of Biological Chemistry *285*, 39976–39985. https://doi.org/10.1074/jbc.M110.182915.

3. Lee, J.W., Choi, Y.R., Mok, H.J., Seong, H.A., Lee, D.Y., Kim, G.S., Yoon, J.H., Kim, K.P., and Kim, H.D. (2017). Characterization of the changes in eicosanoid profiles of activated macrophages treated with 20(S)-ginsenoside Rg3. J. Chromatogr. B Analyt. Technol. Biomed. Life Sci. *1065–1066*, 14–19. https://doi.org/10.1016/j.jchromb.2017.09.002.

4. Bardou, P., Mariette, J., Escudié, F., Djemiel, C., and Klopp, C. (2014). jvenn: An interactive Venn diagram viewer. BMC Bioinformatics 15, 293. https://doi.org/10.1186/1471-2105-15-293.
